# A Common Feature of Pesticides: Oxidative Stress—The Role of Oxidative Stress in Pesticide-Induced Toxicity

**DOI:** 10.1155/2022/5563759

**Published:** 2022-01-19

**Authors:** Rasheed O. Sule, Liam Condon, Aldrin V. Gomes

**Affiliations:** ^1^Department of Neurobiology, Physiology and Behavior, University of California, Davis, Davis, CA 95616, USA; ^2^Department of Physiology and Membrane Biology, University of California, Davis, Davis, CA 95616, USA

## Abstract

Pesticides are important chemicals or biological agents that deter or kill pests. The use of pesticides has continued to increase as it is still considered the most effective method to reduce pests and increase crop growth. However, pesticides have other consequences, including potential toxicity to humans and wildlife. Pesticides have been associated with increased risk of cardiovascular disease, cancer, and birth defects. Labels on pesticides also suggest limiting exposure to these hazardous chemicals. Based on experimental evidence, various types of pesticides all seem to have a common effect, the induction of oxidative stress in different cell types and animal models. Pesticide-induced oxidative stress is caused by both reactive oxygen species (ROS) and reactive nitrogen species (RNS), which are associated with several diseases including cancer, inflammation, and cardiovascular and neurodegenerative diseases. ROS and RNS can activate at least five independent signaling pathways including mitochondrial-induced apoptosis. Limited *in vitro* studies also suggest that exogenous antioxidants can reduce or prevent the deleterious effects of pesticides.

## 1. Introduction

The term pesticide is generally used to identify agrochemicals such as bactericides, fungicides, herbicides, insecticides, or rodenticides [1]. Pesticides are a group of chemicals, and sometimes microorganisms (e.g., viruses), that are used for the eradication of insects, weeds, fungi, and bacteria [1, 2]. Pesticides can be grouped into different chemical families, such as organochlorines, organophosphates, organofluorines, carbamates, pyrethroids, bipyridyl herbicides, triazine herbicides, triazoles, and chloroacetanilide herbicides [2]. Globally, about 2 million tons of pesticides are being utilized each year [3]. China is the largest pesticide-producing nation, followed by the United States and Argentina [3]. Due to the continuous rise in the worldwide population, there has been an increase in demand for agricultural products such as pesticides [4].

The World Health Organization has estimated that about 3 million workers in developing countries experience severe poisoning from pesticides each year, of which approximately 18,000 of them eventually die [5]. The broad use of pesticides for agricultural and nonagricultural purposes (e.g., industrial, commercial, and individual households) around the world indicates how important these compounds are, but the adverse risks involved for the environment, wildlife, and human health are not well investigated [6]. Individuals who apply pesticides in agricultural, occupational, or residential settings are at a high risk of direct exposure. However, the general population can also be exposed to pesticides and their degradation products indirectly at low levels through water, air, dust, and food [7]. Pesticide contamination of surface waters has been well documented worldwide and constitutes a major issue that gives rise to concerns at local, regional, national, and global levels [8]. While these indirect exposure routes involve low levels of pesticide, long-term exposure to these routes could be harmful to human health [7]. At low doses of exposure, pesticides do not seem to produce any permanent harmful effects to adult humans [9]. However, individuals who reside close to fields where pesticides are applied and agricultural workers such as mixers, loaders, and applicators, who are in direct contact with pest control agents, exhibit pesticide poisoning [10, 11]. Other epidemiological studies have suggested that high levels of pesticide exposure are associated with increased risk of chronic diseases, including cancers, cardiotoxicity, Parkinson's disease, diabetes mellitus, neurological deficits, birth defects, and reproductive disorders [12, 13]. The increased risk of various diseases may be due to pesticides being absorbed into the body and accumulated in fat, liver, kidneys, and salivary glands [14].

Prometryn, a triazine herbicide, is relatively persistent in waters, soil, and even in air near its production or application sites and was detected at a concentration of 3–6.1 *μ*g/L in different rivers and lakes in Europe [8]. Previous studies have shown that other triazine herbicide was found at levels as high as 21 ppb in groundwater, 42 ppb in surface waters, 102 ppb in river basins in agricultural areas, and up to 224 ppb in Midwestern U.S. streams during the summer of 1996. Triazine concentrations of up to 108 *μ*g/L have been reported in North America rivers [15]. In addition, all triazine herbicides and their degradation products are persistent in the environment, especially in air and water, and these pesticides can damage human cardiac and immune systems and endanger the health of humans, animals, and plants [16].

A large number of hazardous organic chemicals are pesticides, and the Stockholm Convention on Persistent Organic Pollutants states that nine out of the twelve most dangerous and persistent organic chemicals in the environment belong to the organochlorine pesticide group [17]. There have been several calls and campaigns for “less pesticides, safer food” around the world especially in the European Union (EU) where over one million EU citizens from 22 member states supported an initiative to ban a broad-spectrum systemic herbicide called glyphosate due to its possibly carcinogenic effects on humans. However, pest resistance, hygiene control, and the unending demand for agriproducts have resulted in an increase in the formulation of new, more potent pesticides [18]. Long-term exposures to pesticides, due to occupational or environmental exposures, are capable of disrupting the physiology of different organs in the body, including those of the nervous, endocrine, immune, reproductive, renal, cardiovascular, and respiratory systems [19]. Pesticides' effects could be physiological or biological, causing changes at the molecular, cellular, or tissue level. Although the underlying molecular mechanisms of how pesticides induce biochemical changes are not well understood, investigation of previous research on pesticides suggest that pesticides all induce oxidative stress. Oxidative stress is an imbalance between the production of ROS and the ability of the defense system to actively detoxify and neutralize the excess ROS [20]. The present review focuses on oxidative stress induced by dominant pesticide groups (e.g., organochlorines, organophosphates, carbamates, and triazines) with emphasis on oxidative stress biomarkers and ROS generation from genetic and biochemical studies. This review also includes 3 figures and 6 supplementary tables summarizing the signaling pathways and pesticide concentration levels involved in pesticide-induced oxidative stress.

### 1.1. Pesticides and Organ Toxicity

Most pesticides investigated seem to damage many animal organs and tissues (supplemental table 1). Investigation of the damaged tissues suggests a multitarget mechanism, with many different pathways being affected by the pesticides. However, our understanding of the targeting of multiple sites and signaling pathways in cells is limited. While there are very few studies published discussing the toxic effects of pesticides at the systematic level, one of the most widely investigated pesticides, paraquat, is known to cause damage to the kidneys, lungs, heart, gastrointestinal tract, nervous system, and the immune system [21–23]. Some studies have also suggested links between pesticides and cancers. 1,1,1-trichloro-2,2-bis(p-chlorophenyl)ethane (DDT), and its metabolites have been associated with liver, breast, and testicular cancers [24–26]. Due to the lack of specific studies with respect to the many effects of pesticide on various tissues, it is difficult to determine if specific tissue injuries caused by pesticides are unique. The current scientific studies suggest that irrespective of the target tissue, toxicity caused by pesticides is associated with oxidative stress. Some common characteristics of oxidative stress include increased protein oxidation (carbonylation), lipid peroxidation, nucleic acid oxidation (8-OHdG), and changes in the levels of antioxidants such as glutathione and the activities of antioxidant enzymes [27].

### 1.2. Reactive Oxygen Species (ROS) and Pesticides

Reactive oxygen species (ROS) is a term commonly mentioned in biology and medicine. This term can be defined as oxygen-containing reactive species. ROS is a collective term that includes superoxide (O_2_^•−^), hydrogen peroxide (H_2_O_2_), hydroxyl radical (_●_OH), singlet oxygen (^1^O_2_), peroxyl radical (ROO^•^), alkoxyl radical (RO^•^), lipid hydroperoxide (LOOH), peroxynitrite (ONOO^−^), hypochlorous acid (HOCl), and ozone (O_3_) [28]. ROS are byproducts of normal cellular metabolic processes that are required to generate energy for life processes [29]. They are produced in the reactions catalyzed by the electron transport chain, NAD(P)H oxidase, and some other specialized oxidases and are an inevitable by-product of many redox reactions. However, the amount of ROS produced in a cell under normal conditions is relatively small. As such, ROS are able to serve as signaling molecules to regulate biological and physiological processes [30]. Recent evidence also suggests that ROS function as important physiological regulators of intracellular signaling pathways [31].

**Figure 1:**
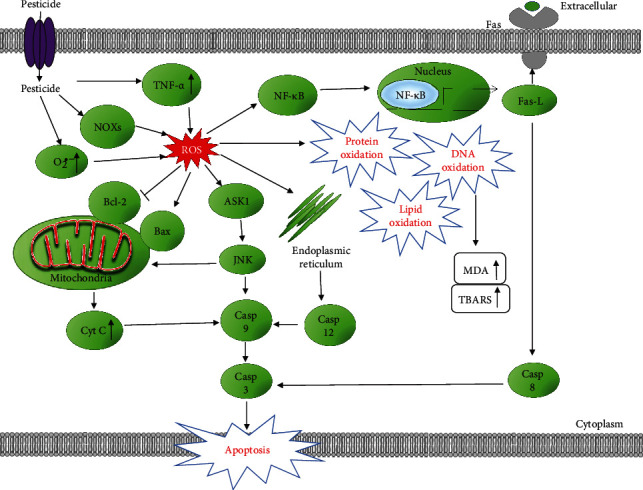
Schematic representation of signaling pathways involved in pesticide-induced reactive oxygen species (ROS) and oxidative stress. Pesticides increase NADPH oxidases (NOXs) and superoxide (O_2_^•−^) levels, which leads to an increase in ROS signaling in the cell. Increased ROS may induce lipid, protein, and DNA oxidation, leading to various toxicities. These stressors lead to activation of TNFR1/TNF-*α*, MAPKs, NF-*κ*B, and the mitochondrial apoptosis pathways. Continued stress leads to cell apoptosis and inflammation.

During normoxia, there is a steady-state balance between ROS and cellular antioxidant systems. However, overproduction of ROS in intra- or extracellular spaces can occur due to exposure to xenobiotics and other environmental factors which can result in the onset of cellular dysfunction and apoptosis [32]. ROS are capable of causing damage to biomolecules leading to cell and tissue injury [32]. Antioxidants act by reacting with ROS and RNS to neutralize or terminate the chain reaction before key molecules in the body are affected [20]. The major enzymatic antioxidant defense mechanisms consist of different forms of superoxide dismutases (SODs), catalase (CAT), and the glutathione peroxidases (GPXs). SODs are thought to provide a first line of defense against oxygen radicals, specifically the superoxide anion (O_2_^•−^), which is the major ROS produced by mitochondrial respiration and various metabolic reactions. The SODs remove the potentially dangerous superoxide anions from biological systems by converting them to H_2_O_2_, while CAT found in peroxisomes catalyzes the conversion of two molecules of H_2_O_2_ to O_2_ and two molecules of H_2_O [33]. The most abundant intracellular antioxidant, glutathione (GSH), is involved in the protection of cells against oxidative stress [34]. However, exposure to pesticides seem to be associated with significantly increased ROS and oxidative stress induction, beyond what the intrinsic cellular antioxidant system can reduce to normal physiological levels (Figure 1, Tables 1 and 2, and Supplemental Tables 1-6).

### 1.3. Reactive Nitrogen Species (RNS) and Pesticides

Reactive nitrogen species (RNS) belong to a family of nitrogen moieties that are closely associated with oxygen [35]. The interaction between exogenously and endogenously produced nitric oxide (NO) with oxidants such as hydrogen peroxide (H_2_O_2_), superoxide anion (*O*_2_^·−^), and reductants such as lithium aluminum hydride (LiAlH_4_) typically gives rise to RNS [36]. RNS can be classified as nitric oxide-derived compounds, including nitroxyl anion (NO^−^) (derived from the reduction of ·NO), nitrosonium cation (NO^+^), higher oxides of nitrogen (NO_2_, N_2_O_4_, etc.), S-nitrosothiols (RSNO), and dinitrosyl iron complexes [37]. NO is a ubiquitous intracellular messenger that regulates physiological functions including neural and cardiovascular activities. However, under pathologic conditions, NO can become deleterious because of its high reactivity with other free radicals, such as *O*_2_^·−^[37]. NO^+^ is created during the removal of one electron from ·NO. NO^+^ can react with nucleophilic centers, producing nitroso compounds. Nitrosyl halides are liberated when •NO reacts with fluorine, chlorine, or bromine [35].

Although RNS play vital roles in numerous biological processes such as the physiological regulation of smooth muscle cells, cardiomyocytes, platelets, and nervous and juxtaglomerular cells, they are harmful to the cells if produced and present in excessive amounts [37, 38]. RNS has pleiotropic properties on cellular targets, including effects even after both posttranslational modifications and interaction of targets with ROS [37]. These effects are likely due to increased levels of RNS reacting with different biomolecules such as lipids, DNA and RNA bases, metal cofactors, and proteins. The interplay of RNS with various cellular components leads to cellular abnormalities, cell injury, and cell death via the induction of nitrosative stress. Nitrosative stress can occur when NO or related species are induced during exposure to certain xenobiotic factors such as pesticides, leading to the nitrosylation of critical protein cysteine thiols (S-nitrosylation) and metallocofactors of proteins. Nitrosylation is the addition of a nitroso (−NO) group to an active metal ion center or thiol of a protein [28].

**Figure 2:**
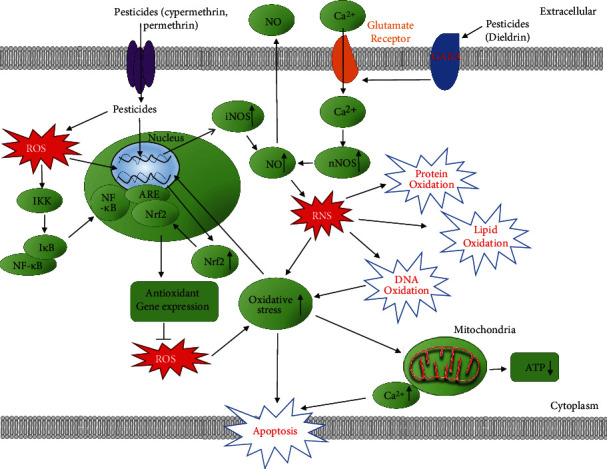
Schematic representation of signaling pathways involved in pesticide-induced reactive nitrogen species (RNS) signaling and oxidative stress. Pesticides including cypermethrin and permethrin increase nitric oxide (NO) and Ca^2+^ levels which increases reactive RNS signaling, thereby increasing oxidative stress in the cell. Pesticides can also lead to Keap1/Nrf2/ARE activation as well as the NF-*κ*B pathway. Increased RNS may induce lipid, protein, and DNA oxidation, resulting in mitochondrial dysfunction and apoptosis.

A comprehensive study of epidemiologic and toxicologic literature implicates oxidative stress, ROS, and RNS as culprits in the damage to lipids, DNA, and proteins induced by pesticides [39, 40].  Figure 2 shows a schematic diagram summarizing some of the major effects of pesticides that lead to RNS and subsequent oxidative stress. Diquat (1,1′-ethylene-2,2′-bipyridinium ion) (DQ), a nonselective quick-acting herbicide, is used as a contact and preharvest desiccant to control terrestrial and aquatic vegetation. Fu et al. found that DQ-induced oxidative stress was caused by ROS. However, this oxidative stress was also partly caused by increased RNS generation by peroxynitrite (ONOO−) generation in hepatocytes [41]. Wang et al. found that permethrin (PER) may exhibit toxic effects on animals by NO generation [40]. A study by Jin et al. revealed that PER significantly increased the mRNA expression of induced nitric oxide synthase (iNOS) after exposure of zebrafish to PER [42]. Another study found increased NO levels in the plasma of rats treated with a low dose of PER (34.05 mg/kg b.w.) [43]. When Afolabi et al. exposed the insecticide cypermethrin (CYP) to rats, they found a significant increase in the plasma concentration of 8-nitroguanine (8-NO2Gua). CYP treatment resulted in over 200% increase in the level of 8-NO2Gua when compared with the control [44]. The elevated plasma 8-NO2Gua level in CYP-exposed rats suggests that CYP plays a role in nitrosative stress and possesses genotoxic and mutagenic potential [44]. The presence of NO and NO metabolites in blood could be a possible source for RNS that causes damage to several organs and tissues.

It has been hypothesized that the damage to cellular macromolecules (nucleic acids, proteins, and lipids) by increased ROS and RNS caused by long-term pesticide exposure leads to cell death and overall tissue damage [45]. It is worth highlighting that RNS and ROS usually work together when they are present in a cell. Under conditions such as pesticide toxicity, NO is generated via the expression of iNOS which then reacts with the superoxide radical to form highly reactive peroxynitrite (ONOO−). ONOO− then causes cellular damage by interacting with biomolecules. One such reaction is that of ONOO− with guanine which results in nitrative and oxidative DNA lesions, such as 8-NO2Gua and 8-oxodeoxyguanosine (8-OHdG), respectively [46]. Previous findings have suggested that 8-NO2Gua formation occurred to a greater extent in cancerous tissues than noncancerous tissues [47]. This supports previous studies that have linked pesticide exposure with cancer [48, 49]. Although RNS is not as well studied as ROS, the experimental data for RNS suggests that RNS can cause tissue damage and should be investigated to a greater extent, especially to determine if all pesticides can cause RNS.

### 1.4. Pesticides and Oxidative Stress Generation

The low molecular weight and high liposolubility of pesticides increases their absorption and toxicity level [50]. Organophosphate (OP) and carbamate pesticides produce their effects through the inhibition of carboxyl ester hydrolases, in particular acetylcholinesterase, which leads to acetylcholine accumulation [50]. Moreover, some studies have suggested that acetylcholinesterase enzyme inhibition is associated with the increase in ROS in agricultural workers exposed to OP pesticides and bipyridyl herbicides (e.g., paraquat (PQ)). Oxidative stress can be induced by an increase in lipid peroxidation and a decrease in antioxidant capacity [51, 52]. Herein, some of the major classes of pesticides and the roles they play in oxidative stress generation are discussed.

### 1.5. Organophosphorus Pesticides

Quinalphos (QP), an organophosphorus (OP) pesticide, is used to control pests on various crops such as vegetables, fruits, cereals, rice, wheat, maize, coconut, tobacco, coffee, tea, sugarcane, jute, and cotton. Dwivedi et al. found that QP enhanced all the levels of adult rat hepatic antioxidant components, namely, SOD, CAT, GPx, and GSH-reductase, which take care of ROS generated *in vivo*. They also found a significant induction of hepatic P450 [53]. A list of OP pesticides and their effects on oxidative stress in different tissues is summarized in supplemental table 4.

Chlorpyrifos (CPF), a crystalline-kind OP insecticide, acaricide, and miticide, is mainly used to control foliage and soil-borne pests on a variety of food and feed crops [54]. Studies have implicated CPF and its derivatives in carcinogenesis [55]. Jung et al. demonstrated in their study that methyl parathion and CPF induced the production of inflammatory cytokines, such as tumor necrosis factor-*α* (TNF-*α*), interleukin-6 (IL-6), and interleukin-1*β* (IL-1*β*) in human hepatocellular carcinoma (HepG2) cells [56]. With regard to examining the deleterious effects of pesticides, Binukumar et al. found that rats chronically exposed to dichlorvos, another OP insecticide, displayed microglial activation with the induction of NADPH oxidase and proinflammatory cytokines (TNF-*α*, IL-1*β*, and IL-6) [57]. *In vitro* studies on human keratinocytes revealed the insecticide monocrotophos significantly increased NO, lactate dehydrogenase (LDH), malondialdehyde (MDA), nuclear changes, proinflammatory cytokines (TNF-*α*, IL-6, and IL-8), and ROS generation [58]. Several other studies conducted in rats exposed to OP pesticides have displayed similarly increased levels of proinflammatory cytokines [59–61].

Pesticides such as DQ do not bind covalently to macromolecules (i.e., lipids, proteins, and nucleic acids). However, they cause oxidative stress by generating ROS intracellularly via the reduction–oxidation (redox) cycling processes. DQ can easily cross the cell membrane and enter the cell through the dopamine transporter (DAT). While in the cell, DQ is reduced by receiving a single electron from NADPH, which serves as the main source of reducing equivalents in cells. This reaction forms NADP^+^ and a highly unstable DQ^+●^, which, in turn, transfers an electron to molecular oxygen (O_2_) to generate O_2_^•−^. This process goes on continuously, even in small amounts, to generate large quantities of O_2_^•−^ and these oxygen radicals are neutralized spontaneously or enzymatically via SOD to produce H_2_O_2_. However, with the large increase in ROS production, the cellular protective mechanisms, either nonenzymatic components (e.g. GSH, thioredoxin, selenium, and vitamins C and E) or antioxidant enzymes (e.g., SOD, GPx, glutathione peroxidase (GR), and CAT), become overwhelmed, resulting in oxidative stress and, consequently, apoptosis [62].

Uchendu et al. indicated that CPF and deltamethrin ((S) a-cyano-3-phenoxybenzyl-(1R)-cis-3-(2.2-dibromovinyl)-2,2-dimethylcyclopropane carboxylate (DM)), which belong to the OP and pyrethroid pesticide groups, respectively, induced oxidative stress due to the generation of free radicals and alteration in antioxidant defense mechanisms. They used a mixture of OP and pyrethroid insecticides, which are common insecticides used by farmers and stored near grains in some countries such as Nigeria [63]. The Uchendu et al. study showed that rats exposed to CPF and deltamethrin, either individually or in combination, had significantly lower levels of CAT, SOD, and GPx and significantly increased levels of MDA compared to the control group [63]. It was suggested that the elevated MDA concentration was due to increased lipid peroxidation, which was induced by excessive production of ROS. The decreased activities of the antioxidant enzymes in the rats exposed to OP and pyrethroid pesticides may be due to the direct deleterious effects of ROS [63].

Another study conducted by Ojha and Gupta indicated that commonly used OP pesticides such as CPF, methyl parathion (MPT), and malathion (MLT) induced apoptosis and DNA interstrand crosslink formation [64]. Ojha and Gupta showed that all OP pesticides significantly increased caspase-3 and caspase-9 activities in rat lymphocytes [64]. Their findings support the suggestion that elevated programmed cell death or apoptosis arises in the presence of oxidative stress and activated caspase-3 and caspase-9 play a role in the breakdown of several cellular components related to DNA repair and regulation during apoptosis [64].

Multiple studies have explored the tendency of OPs to cause cytotoxicity, DNA damage, and disturb oxidative balance, which leads to oxidative stress. In one study, Lu and Yu evaluated the effects of profenofos (PFF) on rat adrenal pheochromocytoma (PC12) cells. They found that PFF and its enantiomers significantly increased intracellular ROS and MDA levels in treated PC12 cells when compared to the control [65]. Their results showed that PFF treatment resulted in a significant increase in the expression of copper/zinc superoxide dismutase (Cu-ZnSOD), glutathione-s-transferase (GST), and CAT. They also found a significant upregulation in heat shock protein (HSP 70 and HSP 90) mRNAs in PC12 cells exposed to PFF. This suggests that the increased HSPs were playing a protective role against oxidative damage [65].

A common feature of all of these publications is that organophosphorus pesticides activates ROS cellular defenses (such as increased SOD, CAT, and GST) in many cell types and tissues. However, the antioxidant protective pathways do not seem to be enough to prevent cell and tissue damage as apoptosis is a common outcome of treatment with these pesticides (supplemental table 2).

### 1.6. Pyrethroid Insecticides

**Figure 3:**
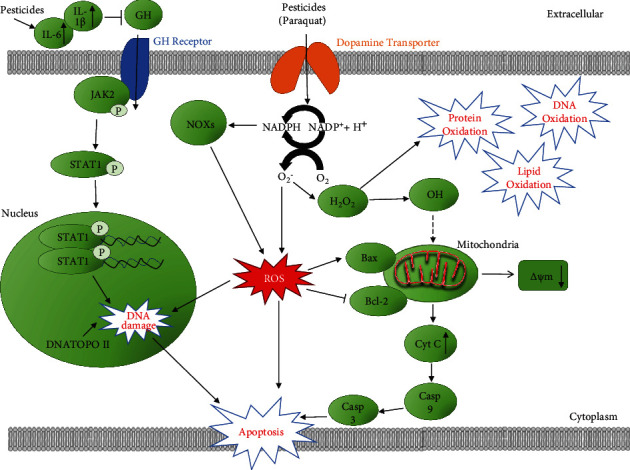
Schematic diagram of the mechanism by which pesticides affect DNA damage and mitochondrial function. Pesticides induce inflammatory cytokines (IL-6, IL-11, and IL-1*β*) and caspases that inhibit growth hormone (GH) thereby causing reproductive and birth defects in humans. Phosphorylation of the C terminal of STAT1 at residue 727 enhances the activity of other factors such as p53 that leads to DNA damage. Together with the action of DNA topoisomerase II, these molecules can cause DNA damage and eventually apoptosis. Pesticides can also induce NOXs and O_2_^•−^ and result in an increase in ROS that leads to mitochondrial dysfunction and activates the mitochondrial apoptosis pathway. _•_OH may also be causing mitochondrial stress. Δ*ψm*: mitochondrial membrane potential.

Permethrin (PER) is a type 1 pyrethroid insecticide. It is the most commonly used pyrethroid in the US and many other countries because of its high activity as an insecticide and its low mammalian toxicity [66]. PER can be used as a fungicide or insecticide for wood preservation purposes and can be found in lice shampoos or scabies treatment which increases their potential to cause harm to human health due to their widespread use around humans [67]. Due to the worldwide use of permethrin, humans and animals may have had exposure to this compound [67]. Studies have shown that pesticides in the pyrethroid family have a role in weakening the immune system because they can induce leukocytosis, decrease natural killer (NK) cell counts, and increase the cluster of differentiation antigen 4/cluster of differentiation antigen 8 (CD4+/CD8+) ratio [68]. A study by Gabbianelli et al. showed that 10 *μ*M of PER and its metabolites, 3-phenoxybenzyl alcohol (3-PBAlc), 3-phenoxybenzaldehyde (PBAld), and 3-phenoxybenzoic acid (3-PBA), significantly increased apoptosis in rat polymorphonuclear neutrophils (PMNs) [69]. Moreover, pyrethroids could alter the metabolism of catechol estrogens through the action of peroxidases, leading to the production of semiquinones and quinones, which are capable of forming DNA adducts [70]. In addition, quinones potentially affect DNA topoisomerase II, an enzyme that participates in DNA repair and recombination, which could lead to breaks in certain susceptible sites of the genome (breakpoint cluster regions of certain genes), modifying DNA topology through the induction of double-strand breaks (DSB) that need to be rejoined [70]. Furthermore, there are genes, such as mixed lineage leukemia (MLL), with particular susceptibility to the breakage by DNA topoisomerase II; the inhibition of this enzyme produces ruptures in this gene which participates in diverse oncogenic fusions driving the leukemogenic process [70] (Figure 3).

Like organophosphorus pesticides, pyrethroid insecticides also increase the levels of antioxidant enzymes and induces apoptosis (supplemental table 2). The lack of detailed studies with respect to the effects of many pesticides on the immune system limits what can be concluded from pyrethroid pesticide studies. However, the current data suggests that pyrethroid pesticides may have a greater effect on weakening the immune system than other pesticides.

### 1.7. Organochlorine (OC) Pesticides

Organochlorine (OC) pesticides are synthetic pesticides that belong to a group of chlorinated hydrocarbon derivatives, which are widely used in the chemical industry and in agriculture [1]. The chemicals identified as OC pesticides have been classified as persistent organic pollutants (POPs) because they have high persistence in the environment [1]. Despite their effective control of malaria and typhus fever, the majority of OC pesticides have been banned in most highly developed countries due to their high toxicity, slow degradation, and bioaccumulation [71]. OC insecticides such as dichloro-diphenyl-trichloroethane (DDT), hexachlorocyclohexane (HCH), aldrin, and dieldrin are among the most widely used pesticides in developing countries of Asia and Africa [1]. Dieldrin is a highly persistent OC insecticide that was widely used to control soil pests such as grasshoppers, locusts, termites, beetles, and textile pests in the agriculture field [72]. It was also effective in controlling tsetse flies, which are the vector that caused African sleeping sickness (*African trypanosomiasis*) and other tropical diseases including malaria, yellow fever, Chagas disease, Oroya fever, river blindness, and filariasis. The United States Environmental Protection Agency (USEPA) banned and restricted the use of dieldrin in 1974 due to its possible carcinogenicity to human and animal health after many years of widespread use. Since some developing countries are still using this pesticide, humans are still exposed to dieldrin mainly through contaminated foods [72]. Several postmortem studies have suggested that exposure to dieldrin has the likelihood of increasing the incidence of Parkinson's disease because significant levels of dieldrin were detected in the brains from Parkinson's patients, while no dieldrin was detected in age-matched control brains [72]. A list of other pesticides, including some not discussed in the text, and their effects on cell types or model systems are shown in supplemental table 5.

Like with organophosphorus and pyrethroid pesticides, organochlorine pesticides that are currently used can be found at low levels in the environment. The organochlorine pesticides show cellular effects that are similar to other classes of pesticides further suggesting that most pesticides may be causing some or most of their deleterious effects via excessive ROS production.

#### 1.7.1. Paraquat

One pesticide that has been well investigated relative to others is paraquat. Exposure to paraquat is associated with the increased risk of pulmonary fibrosis, as well as lung, brain, and heat injuries [73, 74]. Paraquat can generate several types of ROS intracellularly, including O_2_^•−^, H_2_O_2_, and •HO [75]. Paraquat can interact with nicotinamide adenine dinucleotide phosphate (NADPH) oxidase (NOX) and inducible nitric oxide synthase (iNOS) generating ROS and RNS in the cytosol [76]. Paraquat induced NOX type 1- (NOX1-) mediated ROS generation in dopaminergic cells [77], while it activates NOX type 2 (NOX2) in microglia [78]. High levels of NO can react with superoxide ions to form highly toxic peroxynitrite anions (ONOO–). Paraquat can also disrupt the oxidation of NAD(P)H to NAD(P)^+^ that occurs by the mitochondrial electron transport chain (ETC) complex I, by accepting electrons to form a charged version of paraquat (PQ+). PQ+ can generate superoxide radicals (O_2_^•−^) which can lead to other ROS products such as HO•. Brain mitochondria ETC complex III was also shown to affect the H_2_O_2_ levels induced by paraquat [79].

### 1.8. Signaling Mechanisms through Which Pesticides Induce ROS

Although the precise molecular mechanism by which acute or chronic exposure to pesticides induces oxidative stress and damage remains currently unknown, several events involving different cell signaling pathways such as changes in gene expression, activation, and/or inhibition occur. Understanding the cellular and molecular level changes is needed to elucidate the major pathways involved in pesticide-induced oxidative stress and develop potential protective agents or therapies.

### 1.9. Signal Transducers and Activators of Transcription (STAT)

OP pesticides are amongst the most commonly used pesticides in the US. Several studies have reported that CPF increases the production of free radicals and superoxide by disrupting mitochondrial electron transport chain (ETC) complex I activity depleting the antioxidant defenses [80]. Singh et al. reported that CPF induced a dose-dependent increase in cell death and contributed to oxidative stress by upregulating ROS generation and decreasing GSH levels in dopaminergic neuronal and human mesencephalic cells [80]. Based on the results of CPF-induced dopaminergic cell death, the group hypothesized that signal transducers and activators of transcription 1 (STAT1) regulate CPF-induced ROS production and that elevated ROS eventually leads to apoptotic cell death [80]. STAT proteins belong to a family of latent cytoplasmic transcription factors that play a major role in proliferation, growth, apoptosis, and differentiation within different cell types [81]. Janus kinase- (JAK-) STAT signaling is critical for both neuronal survival and cell death [80]. Upon the binding of ligands to their receptors, activation of JAK takes place and, in turn, phosphorylates STAT1 on tyrosine 701 and serine 727 residues. The phosphorylated STAT1 dimerizes and translocates into the nucleus, where STAT1 binds to the gamma interferon-activated site/interferon-stimulated response element (GAS/ISRE) present on the promoter region of specific target genes that regulate proinflammatory cytokines, NADPH oxidase (NOX), apoptosis, and cell cycle arrest regulators, such as caspases, Fas, and Bax [80]. STAT1 regulates cell death through both transcriptional-dependent expressions of proapoptotic genes and nontranscriptional signaling pathways [81]. In another study, OP pesticides induced a 66% decrease in intracellular ROS levels in STAT1 knockdown (KD) dopaminergic cells in comparison with scrambled small interfering RNA- (siRNA-) transfected cells exposed to the same pesticides [80]. NOX-1, a superoxide-generating NADPH-oxidase isoform, has been shown to regulate ROS generation in some cell types, including, but not limited to, monocytes, macrophages, vascular endothelial cells, and smooth muscle cells [82]. NOX-1 is the main ROS-producing enzyme during inflammation [83]. OP pesticides increased the recruitment of STAT1 to the endogenous NOX-1 promoter suggesting that NOX-1 is transcriptionally regulated by STAT1 [80]. STAT1 plays an important role in regulating ROS generation and antioxidant GSH levels in a NOX-1-dependent manner in neuronal cells treated with CPF, an OP pesticide. Mangum et al. found that OC insecticides induced NOX-dependent ROS generation in human monocytic cells [84]. Together, these data suggest that STAT1 activation of NOX is important for ROS generation in OP pesticide-induced oxidative stress.

### 1.10. TNFR1/TNF-*α* Pathway

Some reports suggest that the death receptor pathway is one of the possible mechanisms that induce oxidative stress. The ligation of cell surface death receptors, such as the tumor necrosis factor receptor (TNFR), enables communication signals of tumor necrosis factor-alpha (TNF-*α*), which leads to the activation of caspase-8 that cleaves effector caspase-3, either directly or indirectly via the mitochondrial route [40]. TNF-*α* is a powerful and potent proinflammatory cytokine produced by macrophages/monocytes during acute inflammation and is responsible for different signaling events within cells, leading to necrosis or apoptosis [85]. The inflammatory responses induced by TNF-*α* are mediated by its interaction with two cell surface receptors, TNFR1 and TNFR [86]. TNF-*α* is also involved in the induction of cytokine production, the activation and expression of adhesion molecules, and growth stimulation [87]. Pacheco et al. showed that increased TNF-*α* levels by itself could induce ROS generation and oxidative stress in the L929 mouse fibrosarcoma cell line [86]. A study in rats exposed to permethrin showed that an increase in the TNF-*α* levels increases ROS generation and decreases the antioxidant defense system, which leads to oxidative stress [40]. Additionally, Jin et al. found that permethrin increased TNF-*α* mRNA expression in a concentration-dependent manner when exposed to zebrafish for 72 hours of postfertilization [42]. Zebrafish is considered a good model for investigating cytokine genes such as TNF-*α* [88]. A study conducted by Tyagi et al. that focused on idiopathic preterm birth documented that significantly higher levels of *β*-HCH (beta-hexachlorocyclohexane) and p,p′-DDE (para, para-dichlorodiphenyldichloroethylene) were observed in maternal blood of preterm birth cases (*n* = 30) as compared to term delivery (*n* = 30) from July 2012 to June 2013 in Delhi, India [89]. Tyagi et al. found that TNF-*α* mRNA expression was 2.31-fold higher in preterm birth cases in comparison to term delivery [89]. This suggests that pesticides might be involved in the induction of proinflammatory pathway genes such as TNF-*α*.

### 1.11. Nurr1 and the NF-*κ*B pathway

Orphan nuclear receptor-related 1 (Nurr1) is a transcription factor that belongs to the nuclear receptor subfamily 4 group A member 2 (NR4A2) family of proteins and plays an important role in the metabolism of dopaminergic neurons [40]. Emerging evidence indicates that impaired Nurr1 function might contribute to the pathogenesis of Parkinson's disease [90]. Nurr1 exhibits anti-inflammatory actions due to its inhibitory activity towards the transcription factor NF-*κ*B in brain tissue [40]. Carloni et al. reported in their study that permethrin induced an increase in the expression of the proinflammatory NF-*κ*B transcription factor and a decrease in Nurr1 gene expression [91]. Another study conducted by Fedeli et al. showed that permethrin increased proinflammatory cytokine TNF-*α* expression and decreased IL-1*β*, IL-2, and IL-13 expression in the oldest treated rats [92]. These results suggest that TNF-*α*, Nurr1, and NF-*κ*B pathways may be partly responsible for some of the mechanisms related to oxidative stress caused by pesticides.

### 1.12. Protein Kinase C Signaling Pathway

Kitazawa et al. reported that caspase-3-mediated proteolytic cleavage of protein kinase C (PKC) *δ* contributed to apoptosis of dopaminergic PC12 cells following exposure to dieldrin [72]. PKC can be grouped into a family of serine/threonine kinase enzymes that belong to the AGC (cAMP-dependent, cGMP-dependent, and protein kinase C) superfamily of protein kinases [93]. They are protein kinase enzymes that are able to change enzyme activity, cellular location, or association with other proteins via phosphorylation of hydroxyl groups on serine and threonine residues, resulting in a functional change of the target protein [94]. Kitazawa et al. found that exposure of PC12 cells to dieldrin triggered both a dose-dependent release and a time-dependent release of cytosolic cytochrome C which is consistent with previous literature that suggests that increased ROS production induces or triggers mitochondrial cytochrome C release into cytosol [72]. Additionally, one of the most studied caspases that plays a critical role in execution of apoptosis, caspase-3, was found to be significantly activated following dieldrin exposure. Furthermore, exposure to dieldrin resulted in the proteolytic cleavage of native PKC*δ* over a period of 5 hours [72]. Kitazawa et al. suggest that the proposed mechanism for dieldrin-induced apoptosis in dopaminergic cells was that ROS production triggers cytochrome C release, which activates caspase-9 and caspase-3 and in turn cleaves PKC*δ*, resulting in apoptotic cell death. These results implicate PKC as a signaling pathway involved in pesticide-induced oxidative stress. Further studies on PKC involvement in pesticide-induced cellular changes are needed.

### 1.13. NF-*κ*B Signaling Pathway

Another OP that was extensively used before it was banned and globally phased out due to high toxicity is endosulfan. Endosulfan is primarily used to control a number of insects on food crops like tea, fruits, and vegetables and on grains and can be used as a wood preservative. Endosulfan can be released into the air, water, and soil in areas where it is applied as a pesticide [95]. A mortality study reported an increased incidence of Parkinson's mortality in rural California counties with high use of agricultural pesticides [96]. Jia et al. examined endosulfan and zineb individually and in combination for their potential to stimulate oxidative stress in human neuroblastoma cells (SH-SY5Y) *in vitro* [97]. They found that exposure to endosulfan and zineb significantly increased intracellular H_2_O_2_ and O_2_^∙−^ and production in neuroblastoma cells in a dose-and time-dependent manner which indicates that both pesticides induce oxidative stress [97]. Jia et al. also showed that the caspase-3 activity was significantly elevated in cells treated with endosulfan and zineb when compared with that of the control cells. The activity and expression of NF-*κ*B, a ubiquitous transcription factor, which serves as an indicator of oxidative stress, had significantly higher levels in neuroblastoma cells treated with endosulfan and zineb individually or in combination [97]. These results suggest that the oxidative stress induced by pesticide exposure to cells contributes, at least in part, to the activation of the NF-*κ*B signaling pathway. It can be partly extrapolated from this study that combination or exposure to two or more pesticides causes great harm to the health of farmers, workers, and other individuals who are at a higher disposition to pesticide exposure.

### 1.14. Endoplasmic Reticulum (ER) Stress

The ER serves many functions including the assembly, folding, posttranslational modification, and transport of proteins. In addition, the ER stores calcium which is essential for muscle contraction. ER stress occurs when the protein folding capacity of the ER is overwhelmed, and cells with ER stress are characterized by an accumulation of misfolded proteins inside the lumen of the ER. ER stress could be induced by several conditions including hypoxia, nutrient deprivation, and pesticides. If the ER stress is severe or extended, apoptosis could be induced [18]. Several pesticides such as chlorpyrifos, 2,4-dichlorophenol, deltamethrin, and paraquat have been shown to induce ER stress. Many of these pesticides also induce apoptosis but research suggests that pesticides induce ER stress and apoptotic cell death via different pathways.

### 1.15. Nonmitochondrial Apoptosis Pathway

Apoptosis is a form of programmed cell death that is used to remove unwanted cells. This process is generally characterized by morphology changes including DNA fragmentation, cell shrinkage, and mRNA decay. Pesticides have been documented to induce apoptosis by triggering several different signaling pathways including intrinsic pathways involving the mitochondria and DNA damage as well as extrinsic pathways such as modulation of death receptors [98, 99]. Organophosphorus pesticides like monocrotophos, profenofos, chlorpyrifos, and acephate induce apoptosis in cultured human peripheral blood lymphocytes [100]. Chlorpyrifos and cypermethrin induce apoptosis in human SH-SY5Y neuroblastoma cells [101], while malathion induces apoptotic cell death in N2 neuroblastoma cells [102]. Chlorpyrifos action may be via FAS/TNF signaling pathways [101]. Although pesticides are typically in low concentrations in rivers, lakes, and surface water, these low concentrations have been documented to induce DNA damage and apoptosis in fish. Pyrethroid pesticides are known to be up to 1000 times more toxic in fish than in mammals and birds because of its high absorption into the gills [103].

### 1.16. Mitochondrial Apoptosis Pathway

A common dysfunction associated with oxidative stress is mitochondrial dysfunction [104]. In some cases, mitochondrial dysfunction causes ROS, while in some cases, ROS could cause mitochondrial dysfunction. The complexes in mitochondria are the main site for ROS production, and many pesticides have been shown to inhibit mitochondrial complexes [105, 106]. As such, it is likely that a major contributor to oxidative stress is the ROS produced by dysfunctional mitochondria. In mammals and fish, mitochondrial dysfunction is often associated with ER stress and apoptosis [103, 107].

Pentachlorophenol (PCP) and its metabolite, tetrachlorohydroquinone (TCHQ), decreased the antioxidant GSH level in the mouse liver and drastically increased lipid peroxidation via the abundant production of urinary 8-iso-prostaglandin F2*α* (8-iso-PGF2*α*) [108]. Taking into consideration existing and emerging evidence, the mitochondrial apoptosis pathway is another possible mechanism that is involved in pesticide-induced oxidative stress. B cell lymphoma 2 (Bcl-2) and BCL2-associated X (Bax) are the main mitochondrial integrity regulators in this pathway. They also influence cytochrome c release and caspase activation. Bcl-2 and Bax are two well-known proteins associated with cell death but possess opposite function. The Bcl-2 protein functions as a suppressor where it prevents apoptosis by its antioxidative activity, while the Bax protein functions as a promoter of apoptosis [40]. After mitochondrial damage, Bax is translocated from the cytosol to the mitochondria and a significant decrease in Bcl-2 expression also occurs. Due to high levels of ROS from pesticide exposure, mitochondrial cytochrome c is released into the cytoplasm, which is a critical apoptotic event [40]. Chen et al. found that TCHQ increased the expression of Hsp 70 but decreased the expression of the Bcl-2/Bax ratio and cellular apoptosis susceptibility (CAS), the genes that play a role in apoptotic and necrotic processes, in liver cells. The ratio of Bcl-2/Bax protein may account for the survival or death of intoxicated cells [108]. Their results corroborate the involvement of the mitochondrial apoptotic pathway in pesticide-induced oxidative stress.

### 1.17. Autophagy

Autophagy is a normal process that irreversibly degrades damaged or unwanted eukaryotic cell components. Like apoptosis, it is a form of programmed cell death but autophagy involves different pathways from apoptosis [109]. In mammals, the autophagy process involves the formation of autophagosomes (vesicles) that fuse with the lysosome. Autophagy is important in reducing the effects of oxidative stress on cells [110].

Several pesticides have been shown to increase autophagy. CPF, which was described earlier to increase apoptosis, induces autophagy in neuronal cells [111]. In one study, pretreatment of SH-SY5Y neuronal cells with rapamycin (autophagy inducer) resulted in reduced CPF toxicity (less cell death) while inhibition of autophagy resulted in increased CPF toxicity [111]. In another study in SY5Y neuronal cells, Dai et al. found that CPF induced PTEN-induced putative kinase 1 (PINK1)/parkin-regulated mitophagy (a selective form of autophagy) [112]. Experiments using the pesticide fipronil suggest that autophagy is important in reducing the effects of pesticides. In one study, autophagy was found to increase the viability of cells treated with fipronil [113]. The mechanism of action for improved cell viability may be decreased caspase 3 levels resulting in low levels of fipronil-induced apoptosis [113]. Several investigations also suggest that paraquat induces autophagy. In an interesting study, knockout of the innate proinflammatory mediator Toll-like receptor 4 (TLR4) lessened paraquat-induced cardiac dysfunction [114]. A potential mechanism for the reduced paraquat-induced cardiac dysfunction may be via the regulation of AMPK-mediated cardiac autophagy [114]. In rat adrenal pheochromocytoma PC12 cells, rapamycin significantly decreased paraquat-induced cellular toxicity suggesting that basal autophagy has a protective role in cytotoxicity caused by paraquat [115].

### 1.18. Mitogen-Activated Protein Kinases (MAPKs)

The mitogen-activated protein kinases (MAPKs) are serine/threonine-specific protein kinases that phosphorylate their own dual-serine and threonine residues (autophosphorylation) or those found on their substrates, to activate or deactivate their target [116]. They are involved in regulating cellular processes such as proliferation, stress response, energy metabolism, gene expression, differentiation, proinflammation, mitosis, cell survival, apoptosis, and immune defense [117]. Chen et al. showed that exposure of TCHQ activated c-Jun NH2-terminal kinase (JNK) and p38 in NIH3T3 fibroblast cells [108]. Herein, this further demonstrates that the cascades of the MAPK (JNK, p38 MAPK, and extracellular signal-regulated protein kinase (ERK)) signaling pathway is involved in TCHQ-induced oxidative stress. Pentachlorophenol (PCP) is a restricted use OC pesticide, used industrially as a wood preservative for railroad ties, utility poles, and wharf pilings and used extensively as a biocide in the leather and textile industries [118]. It is highly effective against decay from fungus and damage from wood-boring insects in timbers [119]. Its molecular structure is that of a phenol group (aromatic ring) with five chlorine atoms which makes it a persistent organic pollutant. PCP has been detected in food and several consumable products. PCP has also been found in groundwater in micromolar concentrations. Even higher levels of PCP (0.7 mM) have been reported in the vicinity of industrial point sources of chlorophenols [120]. Due to the difficulty in the degradation of PCP in the environment, its use has been banned by countries which signed the Stockholm Convention with exception to the US. The IARC (International Agency for Research on Cancer) categorized PCP as carcinogenic to humans (group 1) based on epidemiological studies that showed that exposure to PCP causes non-Hodgkin lymphoma in humans [108]. Several studies have reported that exposure to PCP increases the risk of nasal carcinoma and soft tissue sarcoma and induces hepatocellular carcinomas/adenomas, hemangiosarcomas, and pheochromocytomas in a chronic tumorigenesis mouse animal model [121, 122]. Wispriyono et al. found that 20 *μ*M of PCP and its metabolite, TCHQ, markedly increased the number of apoptotic cells and induced DNA fragmentation in Jurkat human T cells after 10 hours of incubation. Notably, they discovered that after 1 hr of incubation, 20 *μ*M of TCHQ phosphorylated all the MAPKs examined (i.e., extracellular signal-regulated protein kinase (ERK), p38, and c-Jun NH2-terminal kinase (JNK)). They went on to show that TCHQ-induced apoptosis disappeared almost completely when treated with both the p38 inhibitor (SB203580) and MAPK/ERK kinase inhibitor (U0126) at the same time. Wispriyono et al. came to the conclusion that p38 and ERK are likely important signal transduction pathways involved in apoptosis in the human T cell line exposed to PCP metabolite [123]. CPF (50 *μ*M) induced redox imbalance altering the antioxidant defense system in breast cancer cells as well as increased formation of intracellular ROS and RNS. Finally, it was demonstrated by Ventura et al. that the main mechanism involved in the inhibition of CPF-induced cell proliferation is an increment of p-ERK1/2 levels mediated by H_2_O_2_ in breast cancer cells [124]. Apoptosis signal-regulating kinase 1 (ASK1) is a member of the mitogen-activated protein kinase (MAPK) family. ASK1 activates c-jun N-terminal kinase (JNK) and p38 in response to various stimuli including oxidative stress, endoplasmic reticulum stress, proinflammatory cytokines, infection, and calcium influx. ASK1 activates JNK and p38 by directly phosphorylating, and thereby activating, their respective MAP2Ks (also called mitogen-activated kinase kinase (MKK)), MKK4(SEK1)/MKK7, and MKK3/MKK6 [125]. Meijles et al. found that activation of cardiac ASK1 is ROS dependent in neonatal rat cardiomyocytes from perfused hearts where H_2_O_2_ activated ASK1 which suggests that ASK1 is selectively activated by ROS [126]. When Niso-Santano et al. exposed human neuroblastoma SH-SY5Y cells to 100 *μ*M PQ for 24 h, they found that paraquat increased ASK1 expression and nuclear apoptosis was significantly increased in PQ-treated cells [127].

### 1.19. Keap1/Nrf2/ARE Pathway and Ca^2+^ Signaling

The Keap1/Nrf2/ARE pathway plays a major role in the regulation of cytoprotective responses to endogenous and exogenous stresses caused by ROS [128]. There are four components involved in the Nrf2/Keap1 pathway; they are (a) the nuclear factor erythroid2-related factor 2 (Nrf2), (b) the actin-binding Kelch-like ECH-associated protein 1 (Keap1), (c) a group of small musculoaponeurotic fibrosarcoma (Maf) proteins, (d) and antioxidant response element (ARE) which are important for the antioxidant response in this pathway [116]. Nrf2 is a transcription factor that binds to the antioxidant responsive element (ARE) in DNA that induces the expression of a group of detoxing enzymes and antioxidant proteins/enzymes. Gene expression of heme oxygenase-1 (HO-1) and NAD(P)H dehydrogenase and others are regulated by Nrf2 [74–77]. Animal models that have increased Nrf2 levels show increased protection against oxidative stress [129, 130], while Nrf2 gene knockout mice have a higher susceptibility to oxidative damage [131, 132]. Keap1, a cysteine-rich protein, acts as an adaptor protein for a Cul3-dependent E3 ubiquitin ligase complex and supports ubiquitination of Nrf2 which then gets degraded by the ubiquitin proteasome system [116]. Consequently, the gene knockout of Keap1 results in constitutively hyperactive Nrf2 signaling.

The generation of excessive ROS from exposure to pesticides leads to the progression of oxidative stress in cells resulting in an increase in the oxidation or conjugation of key cysteine residues in Keap1. These modifications typically weaken its ability to act as an E3 ligase adaptor. As a result, Keap1 loses its ability to promote ubiquitination and degradation of Nrf2. Ultimately, Nrf2 dissociates from Keap1, leading to decreased proteasomal degradation of Nrf2, accumulation of free Nrf2 in the cytosol, and translocation of Nrf2 into the nucleus. Following Nrf2 translocation into the nucleus, it heterodimerizes with small Maf-binding proteins and binds to ARE. This binding ultimately activates ARE-dependent gene expression and initiates the transcription of antioxidant genes [116, 128]. These genes include NAD(P)H: quinine oxidoreductase 1 (Nqo1), heme oxygenase-1 (HO-1), *γ*-glutamylcysteine ligase (Gcl), microsomal epoxide hydrolase (Eh-1), GSTs, sulfiredoxin 1 (Srxn1), multidrug resistance-associated proteins (Mrps), bile salt efflux pump (Bsep), and carboxylesterases (Ces) [128, 133]. In a study conducted by Carloni et al., PER increased Nrf2 gene expression in the cerebellum of rats [91]. Another study found increased mRNA expression of Nrf2 (1.62-fold) and the intracellular Ca^2+^ influx in rat heart cells from 500-day-old rats exposed to PER during their early life (6th to 21st day of life) [134]. This suggests that the Keap1/Nrf2/ARE and Ca^2+^ signaling pathways might be involved in the toxic effect induced by PER. The overexpression of Nrf2 and the increased Ca^2+^ level might be due to epigenetic mechanisms that sustain the memory of pesticide contact, despite the fact that the exposure has ended [134]. Dou et al. found that the Nrf2/ARE pathway is involved in oxidative stress when induced by PQ in human neural progenitor cells (hNPCs) [135]. They detected significant upregulation in cytoplasmic and nuclear Nrf2 expression in hNPCs when exposed to 10 *μ*M of PQ. As a result of Nrf2 increase, they examined Nrf2-ARE-dependent genes and found that HO-1 and Nqo1 mRNA expression was significantly increased at 10 and 100 *μ*M after PQ treatment for 24 hours [135].

Deltamethrin ((S) a-cyano-3-phenoxybenzyl-(1R)-cis-3-(2.2-dibromovinyl)-2,2-dimethylcyclopropane carboxylate (DM)), one of the most potent pyrethroid insecticides with a cyano substituent [136], is used to control apple and pear suckers, plum fruit moth, caterpillars on brassicas, pea moth, aphids (apples, plums, and hops), winter moth (apples and plums), codling and tortrix moths (apples), and numerous insect pests of field crops [137]. DM plays a key role in controlling malaria vectors and is used in the manufacturing of long-lasting insecticidal mosquito nets [138]. It acts as a neurotoxin causing a prototypical type II neurological syndrome characterized by jerking leg movements and progressive writhing convulsions [136]. Treatment with DM increased free radicals in the hippocampus of rats and increased ROS in PC12 cells suggesting that DM exposure resulted in oxidative damage. The authors showed that DM caused a significant increase in cytoplasmic and nuclear Nrf2 protein expression in the cerebral cortex and hippocampus tissue. HO-1 mRNA levels were significantly elevated in tissue from both cerebral cortex and hippocampus tissues when exposed to DM [136]. Hence, they detected a marked increase in Nrf2 protein, HO-1 mRNA, and free radicals *in vivo* in response to DM. Their findings show that Nrf2 translocation from the cytoplasm to nucleus is initiated *in vivo* and is most likely a response to the DM-dependent induction of free radicals (Figure 2). Although the role of Ca^2+^ signaling in pesticide-induced cellular changes needs to be more thoroughly investigated, the Keap1/Nrf2/ARE pathway involvement in cytoprotective responses to pesticides is well supported by the current experimental data.

### 1.20. Possible Signaling Mechanisms through Which Pesticides Induce RNS

Kanthasamy et al. reported that dieldrin can cross the blood-brain barrier and can also be stored in adipose tissue with a half-life in humans of approximately 300 days, due to its lipophilicity [139]. Dieldrin targets neuronal ion channels in the brain through inhibition of the GABA(A) receptor, which results in hyperexcitation and a massive influx of Ca^2+^ via glutamate receptor channels. This Ca^2+^ influx can induce neuronal NOS, further increasing the production of ROS/RNS in the brain. Ca^2+^ plays an important role in numerous cellular processes including mediating cellular proliferation, apoptotic processes, the induction of oxidative stress, and physiological functions (Figure 2) [140, 141]. The detection of 3-nitrotyrosine residues on intracellular proteins exposed to different pesticides such as maneb, rotenone, and dieldrin suggests a role of RNS in diseases such as Parkinson's disease [45].

### 1.21. Possible Mechanisms to Reduce Oxidative Stress Induced by Pesticides

One of the major mechanisms that the body implements in fighting external toxic and harmful agents involves the immune system. The immune response consists of the antigen-non-specific response (innate) and the antigen-specific response (adaptive) [9]. Several experimental studies have reported that exposure to pesticides can exert damaging effects on the immune system [19, 42, 68, 85]. Immunocompetent cells secrete inflammatory mediators, such as cytokines, chemokines, ROS, and RNS. In particular, cytokines can regulate innate or adaptive immunity, hematopoiesis, inflammatory processes, and many other cellular activities through specific binding to their respective receptors [9].

Sometimes, the endogenous antioxidant system becomes incompetent and cannot scavenge the induced oxidative stress [142]. Several studies have reported the potential protective effect of exogenous antioxidant vitamins and minerals against pesticide-induced toxicity in animal models that exhibit alterations in their enzymatic antioxidant system [4, 143–147]. It is important to study the potentially harmful effects of pesticide exposure and various significant methods to mitigate these adverse effects. This section briefly documents the protective role of antioxidant vitamins like vitamins C and E, minerals like zinc, and other naturally occurring antioxidants like N-acetyl cysteine and epicatechin, against pesticide-induced oxidative stress in animal models.

### 1.22. Vitamin C

Vitamin C (ascorbic acid) is a water-soluble antioxidant. It has been shown to react directly with superoxide and hydroxyl radicals to neutralize ROS and reduce oxidative stress [4]. It has been suggested that vitamin C acts as a chain-breaking antioxidant that stops the propagation of peroxidative processes, thereby reducing lipid peroxidation caused by pesticides. Vitamin C can also do a one-electron reduction of lipid hydroperoxyl radicals via the vitamin E redox cycle [4]. A study conducted with male albino Wistar rats by Rai et al. found that vitamin C treatment prevented oxidative stress induced by carbofuran in the erythrocytes of rats [148]. Jaiswal et al. showed that pretreatment of vitamin C with carbofuran provided significant recovery in ameliorating the altered levels of oxidative stress biomarkers. They observed that the levels of MDA, total thiols, and GSH as well as the activities of SOD, CAT, and GST were close to those of the untreated control which suggest that vitamin C is able to provide significant protection from the pesticide's intoxication in the rat heart [149].

El-Gendy et al. studied the protective effect of vitamin C (200 mg/kg b.w.) before and after administration of imidacloprid (a neonicotinoid) in male Swiss albino mice. Their study showed that oral administration of 14.976 mg/kg imidacloprid caused significant elevation of lipid peroxidation levels and the activities of antioxidant enzymes including CAT, SOD, GPx, and GST [147]. However, they reported that vitamin C might ameliorate imidacloprid-induced oxidative damage by decreasing lipid peroxidation levels (measured by thiobarbituric acid-reactive substances (TBARS)) and altering antioxidant defense systems in the liver [147].

Vitamin C treatment of CPF-intoxicated mice decreased the lipid peroxidation level and GST activity, normalized CAT, SOD, and glucose-6-phosphate dehydrogenase activities, and increased the GSH level [150]. In addition, coadministration of propanil with vitamin C ameliorated the harmful effects of propanil in most of the tested oxidative stress parameters in mice liver tissues. Their study suggested that vitamin C could be an important dietary component based on its ability to attenuate propanil-induced hepatotoxicity [151]. All of these studies suggest a clear protective role of vitamin C against pesticide-induced toxicity. Hence, it is likely that other compounds with antioxidant properties can also have significant beneficial effects against pesticide-induced toxicity.

### 1.23. Vitamin E

Vitamin E, present in a biologically active form as *α*-tocopherol, performs as an antioxidant. It is a major lipid-soluble antioxidant present in all cellular membranes and protects against lipid peroxidation [152]. It can act directly with a variety of oxygen radicals, including the peroxyl radical (ROO•), trichloromethyl radial (CCl3), and peroxide (HO•) production. Vitamin E acts by rapidly transferring its phenolic hydrogen atom to lipid peroxyl radicals resulting in the formation of two molecules that are unreactive toward polyunsaturated lipids [4].

Yousef et al. showed that when rats intoxicated with deltamethrin were exposed to vitamin E, the levels of GST and SOD were elevated and the levels of lipid peroxidation were decreased. Thus, vitamin E alleviated the harmful effects of deltamethrin that were observed, exhibiting its beneficial effects in male Sprague Dawley rats [153]. In male Wistar rats, vitamin E has shown its ameliorating effects by restoring the levels of endogenous antioxidant enzymes such as SOD, CAT, GPx, and GST, suggesting its potential antioxidant role against atrazine-induced oxidative stress [154, 155]. A study conducted by John et al. found that treatment of rats with dimethoate and malathion increased the levels of lipid peroxidation in erythrocytes; however, pretreatment of rats with vitamin E before administering dimethoate and malathion showed decreased levels of lipid peroxidation in erythrocytes. Their results display that vitamin E may ameliorate dimethoate- and malathion-induced oxidative stress by decreasing lipid peroxidation and altering antioxidant defense systems in erythrocytes [146]. Ben Amara et al. showed that exposure of rats to dimethoate for 30 days showed pronounced oxidative stress due to an increased lipid peroxidation level and decreased GSH and nonprotein thiol levels [144]. A decrease in GPx, SOD, and CAT activities was also observed, but coadministration of selenium and/or vitamin E through diet in rats improved the altered oxidative stress biomarkers [144]. These results suggest that the use of the antioxidant vitamin E may prevent or reduce many of the damaging effects of some pesticides.

### 1.24. Zinc

Zinc (Zn) is one of the most abundant trace elements in the body and can upregulate various transcription factors and detoxifying molecules (glutathione, SOD, glutathione S-transferase, and hemeoxygenase-1) [4]. Zn also induces the nuclear factor erythroid 2-related factor 2 (Nrf2) to act as an antioxidant and is required for enzymes involved in lipid synthesis and lipoprotein excretion [4]. Saad-Hussein et al. conducted a cross-section comparison study, comparing 80 pesticide sprayers from a small village located within an agricultural area in Upper Egypt with 80 control subjects not occupationally exposed to pesticides [142]. Their subjects had no medical history of chronic diseases, and the pesticide sprayer group had been exposed to pesticides for more than 15 years (15–30 years), without wearing any personal protective equipment. Interestingly, they found that Zn (110 mg) supplementation for 1 month significantly decreased MDA levels and increased SOD, GPx, and Zn levels in pesticide sprayers [142]. Goel et al. showed that zinc treatment to CPF-intoxicated male Sprague-Dawley rats normalized the raised levels of lipid peroxidation to within normal limits [158]. Moreover, they found that Zn treatment in these animals resulted in an elevation in GSH, CAT, and GST levels. Additionally, they found a significant decrease in the levels of SOD. However, results of studies from male and female rats revealed that Zn had greater ameliorating effects in female CPF-intoxicated rats, when compared to males, in reducing oxidative stress parameters [156, 157]. Overall, these results demonstrate the potential protective role of Zn in alleviating the hepatic toxicity, as well as emphasize a role for antioxidants in reducing pesticide toxicity [158]. Differences between females and males are now beginning to be investigated, but the current data suggests that female and male animals show unique differences with regard to pesticide and antioxidant treatments. As such, more targeted research is needed to help determine the differences between males and females.

### 1.25. N-Acetylcysteine

N-Acetylcysteine (NAC) is a nutritional supplement derived from L-cysteine amino acid. NAC is a well-tolerated mucolytic drug that moderates clinging mucous secretions and supports glutathione S-transferase (GST) activity. When administered orally, deacetylation of NAC occurs while passing along the small intestine as well as the liver. Thus, its bioavailability is decreased to 4–10%. NAC stimulates glutathione biosynthesis, promotes detoxification, and acts directly as a scavenger of free radicals, especially oxygen radicals. It is a powerful antioxidant and a potential treatment option for diseases characterized by the generation of free oxygen radicals [159]. Tebuconazole (TEB), a triazole fungicide, is widely used to control fungal growth in vegetables, fruits, and seeds. It can also be used as a biocide preservative for industrial and construction material [160]. Ben Othmène et al. found that TEB increased lipid peroxidation, DNA damage, and p53 and p21 protein levels after 24 h in H9c2 cardiomyoblasts. They also suggested that TEB might induce oxidative stress in cardiac cells via the mitochondrial apoptotic pathway due to the loss of mitochondrial transmembrane potential (ΔΨ*m*), an increase in the Bax/Bcl-2 ratio, an activation of caspase-9 and caspase-3, a cleavage of poly (ADP-ribose) polymerase (PARP), and an increase in mitochondrial superoxide (measured by MitoSOX). However, when they treated cardiomyocytes with the ROS scavenger NAC, there was a decrease in TEB-induced DNA damage and activation of the mitochondrial pathway of apoptosis [161].

Dorval and Hontela showed that rainbow trout (*Oncorhynchus mykiss*) pretreated with NAC following exposure to endosulfan had significantly higher levels of GSH and decreased levels of lipid hydroperoxides (LOOH) [162]. Another study conducted by Cankayali et al. using male Wistar rats cotreated with dichlorvos and NAC found that NAC might prevent lipid peroxidation and decrease the risk of oxidative stress [163]. Finally, Yurumez et al. found that male NMRI mice treated with 250–500 mg/kg Mancozeb (MZB) for 40 days exhibited significantly increased lipid peroxidation, increased protein carbonyl concentration in the testes, and decreased activities of antioxidant enzymes (SOD and CAT). The total antioxidant capacity and GSH content were found to be significantly less in the testes of MZB-exposed mice [60]. However, cotreatment of MZB-exposed mice with NAC reversed the changes in oxidative stress indices found earlier with MZB. They found significantly decreased levels of lipid peroxidation, and the activities of antioxidant enzymes SOD and CAT were maintained near control levels following NAC + MZB cotreatment [60]. As with other antioxidants discussed earlier, NAC seems to also have strong antioxidative stress properties.

### 1.26. Epicatechin

Flavonoids are a large group of natural phenolic compounds with different subclasses that have been described as powerful antioxidants from previous *in vitro* studies [164]. The antioxidant properties of flavonoids are largely dependent on their structure, and the major contributing factor is the presence of 3′,4′-dihydroxycatechol, which has reducing capabilities and influence on the intracellular redox status [165]. Epicatechin is a flavan-3-ol, a subclass of the flavonoids found in green tea, grape, apples, and cocoa [166]. Tea extracts and/or its constituents have been reported to possess pharmacological effects such as anti-inflammatory, antibacterial, antiviral, antioxidant, antitumor, antihyperlipidemic, anticarcinogenic, and cytoprotective effects. Also, it was shown that green tea extract can scavenge nitric oxide (NO) and O_2_^•−^ very effectively [167]. Moreover, Afolabi et al. found that cotreatment of CYP-exposed rats with epicatechin significantly reduced the formation of nitrosative nucleic acids by 51% [44]. Another study conducted to investigate the effects of catechin against PCP-induced cytotoxicity in human erythrocytes found that PCP significantly decreased GSH levels, total sulfhydryl (SH) content, and cellular antioxidant power. PCP treatment also lowered the activity of antioxidant enzymes and inhibited enzymes of glucose metabolism. However, prior treatment with catechin before incubation with PCP increased the GSH level and total SH content in erythrocytes [120]. Maheshwari and Mahmood reported that prior treatment of catechin prevented the oxidative damage of membrane lipids and lowered malondialdehyde and lipid hydroxyperoxide levels to 1.6- and 1.56-fold relative to control values. Finally, they found that catechin decreased intracellular ROS and RNS levels in PCP-treated erythrocytes [120]. In addition, Spencer et al. showed that epicatechin and its *in vivo* metabolite, 3′-O-methyl epicatechin, protected human fibroblasts from hydrogen peroxide-induced oxidative stress by inhibiting caspase-3 activation [165]. These studies suggest that epicatechin significantly mitigates pesticide-induced oxidative modifications in a concentration-dependent manner while not exhibiting any deleterious effect on its own. Catechin may be a potential chemoprotectant against pesticide toxicity, and other structurally related compounds to catechin may also be beneficial, but further experimentation is needed.

## 2. Conclusions

The current experimental evidence from research studies suggests that all classes of pesticides induce oxidative stress, RNS, and ROS in different cell types and animal models and that oxidative stress is one of the most important mechanisms of pesticide toxicity. Albeit not exhaustive due to the large number of pesticides available, this review covers the major classes of commonly used pesticides in the United States and the rest of the world. Pesticide exposure could come from occupational routes as well as from food, water, air, and dust. What is typically lacking in previous reviews and research publications are the molecular mechanisms involved in pesticide toxicity because of the complexity of mechanisms that may be involved with different classes of pesticides. A search of the Internet for publications that attempted to explain the mechanisms involved in pesticide toxicity revealed only two publications with one figure each.

The higher levels of oxidative stress eventually cause cell apoptosis through several pathways: the mitochondrial apoptosis pathway, Keap1/Nrf2/ARE, Ca^2+^, TNFR1/TNF-*α*, Nurr1, STAT1, ASK1, MAPKs, and NF-*κ*B pathways amongst others. Increased levels of ROS and RNS may also affect the ubiquitin proteasome system (UPS) that degrades altered and misfolded proteins [168]. Numerous reports suggest that Kelch-like ECH-associated protein-1 (Keap1), a substrate adaptor protein for a cullin-3 E3-ubiquitin ligase (Cul3)/Ring-Box- (Rbx1-) dependent complex, plays a critical role in the ubiquitination and degradation of Nrf2, IKK*β*, and Bcl-2/Bcl-xL. ROS disrupts Keap1 via modifying reactive cysteines (Cys273, Cys288, and Cys151) and then inducing a conformational change that leads to the release of Nrf2, IKK*β*, and Bcl-2/Bcl-xL from Keap1 and the suspending of their ubiquitination and degradation [169–172]. UPS dysfunction could lead to various cellular malfunctions including proteotoxicity, mitochondrial dysfunction, and apoptosis.

Even though we are starting to understand the mechanisms involved in pesticide toxicity, more research is needed as it is crucial to better understand the molecular mechanisms by which pesticides induce oxidative stress. Interestingly, the T-2 toxin, which is not a pesticide, shows molecular mechanisms for toxicity similar to pesticides. The T-2 toxin induces oxidative stress in numerous cell lines causing oxidative damage to lipids, proteins, and DNA [173]. The T-2 toxin increases MDA content (lipid peroxidation representative) and CAT and SOD activities as well as decreases GSH-Px activity in rat anterior pituitary GH3 cells. Caspase-3, -8 and -9 are significantly induced by the T-2 toxin in a dose-dependent manner. While these T2 toxin-induced mechanisms are similar to pesticides, the T2 toxin also increases the mRNA levels of IL-6, IL-11, and IL-1*β* and inhibits the synthesis and secretion of GH. It is possible that pesticides could be working in a similar manner to T-2 toxin in regard to the induction of interleukins and growth hormone deficiency [174]. Both PCP and DDT increase IL-6 production [175]. Also, previous research found that people regularly exposed to pesticides, such as women and children raised in agricultural areas, had low levels of insulin-like growth factor-1 (IGF-1), which plays an important role in childhood growth. This makes the children at higher risk of developing growth disorders [176]. While studies suggest that antioxidants may be beneficial for reducing pesticide toxicity, further studies should focus on the possible ways to ameliorate the side effects of pesticide exposure not just via exogenous antioxidants but by influencing the signaling pathways involved in pesticide-induced oxidative stress. More studies are urgently needed to determine how sex differences may be involved in pesticide-induced toxicity, as well as if any other cellular pathways are involved in pesticide-related toxicity.

### 2.1. Limitations of Previous Studies

Many of the clinical studies on humans have relatively low sample sizes complicating the interpretation of the data as well as the reliability of that data to make generalized conclusions for different populations. Experimental data on gender-specific effects has not been well investigated, but experimental data suggest that xenobiotics affect males and females differently, so pesticides are likely to have gender-specific effects [156, 157]. It would be of great interest to the public to understand the variations in the response of male and female animals to pesticide exposure. It would also be important to determine if there are gender-specific differences in the possible effects of exogenous antioxidants in the mitigation of pesticide-induced oxidative stress biomarkers (lipid peroxidation, lipid oxidation, and protein oxidation), DNA fragmentation, and apoptosis.

Oxidative modification of proteins and proteolytic pathways compromise the protein quality and cell viability due to oxidative stress that arose from the increased ROS level when exposed to pesticides. These events may be among the most relevant in driving protein toxicity in several pathologies such as neurodegenerative diseases, cardiovascular diseases, cancer, reproductive diseases, and birth defects. However, the experimental data on the role of protein toxicity and the UPS in pesticide toxicity is very limited [177]. Finding novel ways to prevent pesticide toxicity may require understanding the role of the UPS pathway in this process to increase the capacity of proteolytic systems to remove intracellular oxidized proteins.

## Figures and Tables

**Table 1:** The effects of the most commonly used pesticides in the agricultural market sector in 2012 on oxidative stress in different tissues.

Pesticide	Cell type/model system	ROS	SOD	GSH	CAT	GST	GPx	DD	LP	PC	AOC	MMP	Reference
Glyphosate	Human skin keratinocyte HaCaT cells	↑	↓										[178]
	Human liver carcinoma (HepG2) cells						↓	↑			↓		[179]
	Rat heart H9c2 cells											↓	[180]
	Adult albino male rats (liver)			↓					↑				[181]
	*Caenorhabditis elegans*					↑						↓	[182]
	*Chlorella kessleri*		↑	↑	↑				↑				[183]

Atrazine (ATR)	Male and female Balb/c mice	↑		↓									[184]
	Male mice (liver and kidney)		↓		↓	↓			↑		↓		[185]
	Male Wistar rats (erythrocytes)		↑	↓	↑	↑	↑						[155]
	Adult male Wistar rats (testes and epididymis)		↓		↓	↓			↑				[186]
	Male Wistar rats		↓	↓	↓				↑				[187]
	Adult male albino rats		↓	↓	↓		↓		↑				[188]
	Female Wistar rats				↓		↓		↑				[189]
	Murine microglial cells (BV-2)	↑											[190]
	Albino rats			↓	↑		↑	↑	↑				[191]

Metolachlor-S	*Scenedesmus obliquus (green algae)*	↑	↑		↑								[192]
	*Parachlorella kessleri* (microalga)	↑			↑				↑				[193]
	Wheat (*Triticum aestivum* L.)	↑	↑		↓				↑				[194]

2,4-Dichlorophenoxyacetic acid (2,4-D)	*Umbelopsis isabelline (Fungus)* pea (*Pisum sativum* L.)	↑	↑				↑		↑				[195, 196]
	Pea (*Pisum sativum* L.)		↑		↑		↑		↑				[196]
	Nongreen potato tuber callus		↑		↑	↑							[197]
	Male 7-week-old Kunming mice		↓		↓				↑				[198]
	Goldfish gills, *Carassius auratus*		↑		↑		↑		↑	↑			[199]
	*Cnesterodon decemmaculatus*			↑	↑	↑		↑					[200]
	*Acanthospermum hispidum* D.C., Asteraceae weed								↑				[201]
	Rat cerebellar granule cells	↑		↓	↓		↑						[202]
	Wistar rats		↓	↓	↓		↓		↑				[203]
	Wistar Albino rats		↓	↓	↓	↓	↓		↑	↑			[204]
	Male Wistar rats (liver)		↑↓		↓		↓		↑				[205]
	Male Wistar albino rats—plasma, liver, kidney, erythrocytes		↓		↓		↓		↑				[206]
	Male Wistar rats (liver)				↓								[207]
	Female B6C3F1 mice *peritoneal macrophages*			↓									[208]

Metam	*Soil bacteria*				↑↓				↑				[209]

Acetochlor	*Bufo raddei* tadpole liver							↑	↑		↓		[210]
	Female zebrafish	↑	↑		↑		↑						[211]
	Male C57BL/6 mice (testis)		↓	↓					↑				[212]
	GC-1 spermatogonia cell		↓	↓					↑				[212]
	Human liver carcinoma cells (HepG2)	↑	↓				↓					↓	[213]
	Zebrafish				↑		↑						[214]
	Primary human corneal epithelial (HCE) cells							↑	↑	↑			[215]
Chloropicrin	Human retinal pigment epithelial cells (ARPE-19)	↑											[216]
	Human lung epithelial cells (A549)	↑											[217]
	*Gill tissues of Pacific oyster (Crassostrea gigas), blue mussels (Mytilus edulis)*		↑	↑	↑		↑		↑				[218]

Chlorothalonil	*Polychaete Laeonereis acuta*								↑		↓		[219]
	*Fish Danio rerio (gills)*	↑				↑					↑		[220]
	*Fish Danio rerio (liver)*		↑						↑				[220]
	Isolated rat hepatocytes			↓					↑				[221]
	*Botryllus schlosseri hemocytes*			↓									[222]
	*Male Wistar rats (liver)*							↑					[223]
	Fresh water fish, *Channa punctatus*		↓	↓	↓				↑				[224]

Pendimethalin	Male Wistar rats (liver and kidney)		↓	↓	↓	↓		↑	↑	↑			[225]
	Human lymphocytes	↑						↑					[226]
	Rat bone marrow cells	↑		↓	↓			↑	↑				[226]
	*Clarias batrachus* (liver)		↑		↑				↑				[227]
	Fish *Channa punctatus* (brain)		↓	↓	↓	↓			↑	↑			[228]
	Fish *Channa punctatus* (gills, liver, kidney)			↓	↓	↓	↓		↑	↑			[229]
	Chinese hamster lung fibroblast (V79) cells	↑						↑					[230]
	Male mice (spleen and thymus)		↔	↓	↓		↓		↑				[145]

Ethephon	3T3 murine embryonic fibroblast (MEF) cells	↑						↑	↑				[231]
	Spinach (*Spinacia oleracea* L.)				↑								[232]
	*Ipomoea cairica* (Linn.) sweet	↑											[233]
	*Carassius auratus goldfish blood and gills*		↑		↑	↑			↑	↑			[234]

Mancozeb	Carassius auratus Goldfish—liver and kidney		↑		↑		↑		↑	↑			[235]
	Carassius auratus Goldfish—brain								↑	↑			[235]
	*Cassia angustifolia*		↑	↑	↑				↑				[236]
	*Caenorhabditis elegans*	↑				↑						↓	[237]
	*Caenorhabditis elegans*	↑				↑						↓	[238]
	*Drosophila melanogaster*	↑	↓	↓	↑	↑			↑				[239]
	*Rat*-*1 fibroblasts*, peripheral blood mononucleated cells (PBMC)	↑						↑					[240]
	Male NMRI mice		↓	↓	↓		↓		↑	↑	↓		[241]
	Rat thymocytes	↑										↓	[242]
	*Human gastric adenocarcinoma* (*AGS*) cells	↑										↓	[243]
	Immortalized murine mesencephalic dopaminergic (N27) cells	↑		↓				↑				↓	[80]
Chlorpyrifos	Lund human mesencephalic (LUHMES) cells	↑						↑				↓	[80]
	Human neuroblastoma *SH*-*SY5Y* cells	↑											[244]
	Rat adrenal pheochromocytoma (PC12) cells	↑							↑				[245]
	Rat erythrocytes		↓		↓	↓			↑				[156]
	Male Wistar rats		↓		↓		↑		↑				[63]
	Male Wistar rats (aorta, liver, plasma, and kidney)		↑						↑				[246]
	Male Swiss albino adult rats		↓	↓	↓		↓		↑				[247]
	Adult male Wistar rats	↑											[248]
	Male Wistar rats		↑		↑	↓	↓		↑				[249]
	Male Kunming mice	↑	↓	↓	↓		↓		↑				[250]
	Male Wistar rats (liver)											↓	[251]

Metolachlor	*Soil bacteria*				↓↑				↑				[209]
	Lettuce, bean, and pea seeds and leaves		↓		↓		↓						[252]
	8-week-old male rats		↓	↓		↓	↓		↑				[253]

Propanil	Wistar rats, liver		↓	↓	↓	↓			↑				[151]
	Albino rats, liver			↓	↓				↑				[254]
	Common carp (*Cyprinus carpio*) brain		↓	↓	↓				↑	↑			[255]
	Isolated mitochondria from potato tubers (*Solanum tuberosum)*											↓	[256]

Dicamba	Nongreen potato tuber callus		↑		↑	↑							[197]
	*Cnesterodon decemmaculatus*			↑	↑	↑		↑					[200]
	Isolated mitochondria Arabidopsis	↑											[257]
	Chinese hamster lung fibroblast (V79) cells	↑						↑					[230]

Trifluralin	Male Wistar albino rats—kidney, ureter, urinary bladder		↓				↓	↑	↑				[258]
	*Chlamydomonas mexicana*		↑		↑								[259]

Acephate	*Drosophila melanogaster*		↑		↑	↑		↑	↑	↑			[260]
	*Male albino rats (plasma and liver)*		↓	↓	↓		↑		↑				[261]
	*Male rats (erythrocytes)*			↓		↑			↑				[262]
	*Albino rats*		↓	↓	↓				↑				[263]
	*Human sperm*							↑					[264]
	Chinese hamster ovary (CHO-K1) cells			↑		↑	↑						[265]
	Porcine kidney proximal tubule *cell* line (*LLC*-*PK)*	↑							↑				[266]
	Human dopaminergic neuroblastoma cells (SK-N-SH)	↑							↑			↓	[267]
Paraquat (PQ)	Rat lung slices	↑											[268]
	Rat organotypic midbrain slice cultures	↑											[269]
	Rat primary mesencephalic cultures	↑											[270]
	Rat primary mesencephalic cultures	↑											[79]
	Human neural progenitor cells (hNPCs)		↓		↓				↑				[135]
	Human neural progenitor cells (hNPCs)	↑											[271]
	Human plasma								↑		↓		[51]
	Rat brain mitochondria	↑											[79]
	Nongreen potato tuber callus		↑		↑								[197]
	*Amaranthus palmeri*	↑	↑		↑								[272]

Glufosinate	Horseweed, palmer amaranth, kochia	↑	↑		↑				↑				[273]
	*Chlorella vulgaris*		↑		↑				↑				[274]

ROS: reactive oxygen species; SOD: superoxide dismutase; GSH: glutathione; CAT: catalase; GST: glutathione-S-transferase; GPx: glutathione peroxidase; DD: DNA damage; LP: lipid peroxidation; PC: protein carbonylation; AOC: antioxidant capacity; MMP: mitochondrial membrane potential; ↑: increased; ↓: decreased.

**Table 2:** Effects of commonly used conventional pesticide active ingredients in the home and garden market sector in 2012 on oxidative stress in different tissues.

Pesticide	Cell type/model system	Concentration/dose	Oxidative stress markers	Reference
Carbaryl	*Cantareus apertus* (digestive gland)	1 *μ*M	Increased lipid peroxidation, increased activities of CAT, SOD, GPx, and GR, and decreased total oxyradical scavenging capacity	[275]
	*Calothrix brevissima*	10, 20, 30, and 40 mg/L	Increased lipid peroxidation and increased SOD, CAT, and APX activities	[276]
	*Caenorhabditis elegans*	0.5, 1, and 1.5 mM	Decreased SOD activity and increased CAT and GPx activities	[277]
	Mouse neuroblastoma cells (neuro 2A)	10 *μ*M	Increased ROS level, loss of mitochondrial membrane potential, increased proapoptotic gene Bax and caspase-3 expression, and decreased antiapoptotic gene Bcl-2 expression	[278]
	Rat adrenal pheochromocytoma (PC12) cells	100 *μ*g/mL	Increased lipid peroxidation, increased SOD activity, decreased GSH content, and decreased mitochondrial membrane potential	[279]
	Water buffalo (*Bubalus bubalis*)	1 mg/kg	Increased lipid peroxidation, increased activities of GPx, GR, GST, SOD, and CAT, and decreased GSH level	[280]

Permethrin (PER)	Rat polymorphonuclear neutrophils (PMNs)	PER (10 *μ*M)	Increased apoptosis, protein carbonyl, and conjugated diene formation in lipids	[69]
		PER metabolites (3-PBAlc, PBAld, and 3-PBA) (10 *μ*M)	Increased apoptosis, protein carbonyl, and conjugated diene formation in lipids	[69]
	Rat adrenal pheochromocytoma (PC12) cells	PER (10, 20, and 30 mg/L)	PER induced enantioselective oxidative stress and cytotoxicity	[281]
		1*R-trans*-PER (10, 20, and 30 mg/L)	Increased ROS generation and MDA level and decreased the activity of SOD, CAT, and GSH	[281]
		1*S-trans*-PER (10, 20, and 30 mg/L)	The toxic effect on PC12 cells induced by 1*R-trans-*PER was approximately 1.6 times higher than by 1*S-cis*-PER	[281]
	Thymic cells from C57BL/6 mice	PER (150, 300, 600, and 1000 *μ*M	Induced O_2_^•−^ and H_2_O_2_	[282]
		Lindane (37.5, 50, 75, 150, and 200 *μ*M)	PER and lindane mixtures increased SOD activity, had no effect on CAT levels, and inhibited GPx and GSH-R-specific activities	[282]
	Wistar rats	34.05 mg/kg	Increased Nurr-1, Nrf2, and NF-*κ*B p65 mRNA levels in the cerebellum	[91]
	Wistar rats	34.05 mg/kg	Increased plasma lipid peroxidation	[92]
	Male and female 500-day-old rats	4 mL/kg	Increased calcium and Nrf2 gene expression levels in old age	[134]

Cypermethrin	Wistar rats	25 mg/kg	Increased lipid peroxidation and protein oxidation, increased plasma IL-6 and TNF-*α* levels, and increased 8-NO_2_Gua levels	[44]
	Wistar rats	1.5–15 mg/kg	Increased lipid peroxidation	[283]

Deltamethrin	Sprague–Dawley rats (hippocampi)	3.125 mg/kg and 12.50 mg/kg	Increased reactive free radical formation in the brain, increased nuclear Nrf2 expression, and increased HO-1 mRNA levels	[136]
	Rat adrenal pheochromocytoma (PC12) cells	10, 100 *μ*M	Increased intracellular ROS production	[136]
	Male Wistar rats	6.25 mg/kg	Decreased CAT activity, SOD activity, and GPx activity. Increased lipid peroxidation	[63]
Bifenthrin	Human colon carcinoma (HCT116) cell	1/4 IC50, 1/2 IC50, 3/4 IC50, and IC50	Increased ROS production levels, increased lipid peroxidation, increased DNA damage, decreased mitochondrial membrane potential, and increased caspase-3 activity and MAPK activation	[284]
	Male ICR mice	1S-*cis*-BF (5 mg/kg)	Increased hepatic ROS level, increased serum and hepatic lipid peroxidation, decreased GSH activity, increased CAT activity, increased SOD activity, and increased Cat and Ho-1 mRNA levels	[285]
	Human umbilical vein endothelial cells *(HUVECs)*	15, 30 *μ*M	Increased apoptosis	[286]
	Zebrafish	15, 30 *μ*M	Increased intestinal ROS level	[286]

2-Methyl-4-chlorophenoxyacetic acid (MCPA)	Human erythrocytes	250, 500 ppm	Decreased GSH level	[287]
	Human erythrocytes	2.0 mM, 4.0 mM	Increased lipid peroxidation	[288]
	*Ramalina fraxinea*	20, 50, 100 mg/L	Increased lipid peroxidation	[289]

Malathion	Thymic cells from C57BL/6 mice	37.5, 75, 150, 300 *μ*M	Induced O_2_^•−^ and H_2_O_2_	[282]
	Rat erythrocytes	0.13 mg/kg	Increased lipid peroxidation, increased SOD and CAT activities, and increased total-SH content	[146]
	Male Wistar rats (cortex, striatum, cerebellum, hippocampus)	25, 50, 100, and 150 mg/kg	Increased lipid peroxidation, increased protein carbonylation, increased/decreased CAT activity, and increased/decreased SOD activity	[290]
	Prepubertal male mice	200 mg/kg	Increased lipid peroxidation, increased ROS level, decreased SH group, reduced CAT and GPx activities in the liver and kidney, decreased total SOD, Cu/Zn-SOD, and Mn-SOD activities in the liver, and decreased total SOD and Mn-SOD activities in the kidney	[291]
	Male Swiss mice	500 mg/kg	Increased lipid peroxidation, increased ROS level, increased SH group content, and increased testicular activities of SOD, Cu/Zn-SOD, Mn-SOD, Fe-SOD, and CAT	[292]
	Male Wistar rats	250 mg/kg	Increased lipid peroxidation and decreased testicular total antioxidant capacity	[293]
	Male Swiss albino mice	27 mg/kg	Increased lipid peroxidation, decreased testicular activities of SOD, CAT, and GPx, and decreased GSH level	[294]
	Wistar male rats	250 mg/kg	Increased 8-hydroxy-2′-deoxyguanosine (8-OHdG) level, increased NO level, decreased total antioxidant capacity (TAC), increased total oxidant status (TOS), decreased CAT and SOD activities, and increased DNA damage	[295]
	Sprague Dawley rats	200 mg/kg	Increased lipid peroxidation, increased NO level, and decreased GSH level	[296]
	Human liver carcinoma cell (HepG2)	6–24 mM	Increased lipid peroxidation and increased oxidative DNA damage	[297]
	Porcine cumulus-oocyte complexes	750 and 1000 *μ*M	Increased ROS level, increased lipid peroxidation, increased protein carbonylation, increased Cu/Zn-SOD, GST, and G6PD expression levels, and decreased CAT and GPx expression levels	[298]
	Male Wistar rats	25, 50, 100, and 150 mg/kg	Increased mitochondrial superoxide production in the hippocampus, increased lipid peroxidation in the hippocampus and striatum, and decreased complex IV activity in the hippocampus	[299]
	Rohu (*Labeo rohita) liver*	5 *μ*g/L	Increased intracellular ROS level, increased lipid peroxidation, increased activities of CAT, SOD, POD, GSH, GR, GST, and GPx, and increased DNA damage	[300]
	Human erythrocytes	25, 75, 200 *μ*M	Increased lipid peroxidation, decreased SOD, CAT, and GPx activities	[301]
	Female Wistar rats (ovary)	50 mg/kg	Increased lipid peroxidation and decreased GSH content	[302]
	*Allium cepa*	0.05, 0.13, 0.26, 0.39, and 0.52 g/L	Increased lipid peroxidation, increased CAT, GST, and SOD activities, decreased APX and GR activities, and increased DNA damage	[303]
	Male Kunming mice	10^−5^ M	Increased lipid peroxidation, increased •OH level, decreased SOD, GPx, and CAT activities, decreased GSH content, and increased levels of Bax, Bcl-2, and p53 in splenic T cells	[250]

## Data Availability

All information is from published papers and is summarized in supplemental tables.

## Abbreviations

CAT: Catalase
CPF: Chlorpyrifos
CYP: Cypermethrin
DDT: Dichloro-diphenyl-trichloroethane
ER: Endoplasmic reticulum
Fas-L: Fas ligand
Flt: Fms-related receptor tyrosine kinase 1
GPX: Glutathione peroxidase
GR: Glutathione reductase
GSH: Glutathione
HCH: Hexachlorocyclohexane
H_2_O_2_: Hydrogen peroxide
HMOX1: Heme oxygenase 1
iNOS: Inducible nitric oxide synthase
JAK: Janus kinases
JNK: c-Jun N-terminal kinases
KD: Knockdown
MAPK: Mitogen-activated *protein* kinase
MDA: Malondialdehyde
NF-*κ*B: Nuclear factor *κ*B
NO: Nitric oxide
NOX: NADPH oxidase
Nrf2: Nuclear factor-erythroid 2-related factor 2
PER: Permethrin
PKC*δ*: Protein kinase C*δ*
PQ: Paraquat
RNS: Reactive nitrogen species
ROS: Reactive oxygen species
STAT: Signal transducers and activators of transcription
SOD: Superoxide dismutase
TCHQ: Tetrachlorohydroquinone
TBARS: Thiobarbituric acid-reactive substances
TLR: Toll-like receptor
TNF*α*: Tumor necrosis factor *α*
UPS: Ubiquitin proteasome system.

## Supplementary Materials

Supplementary Materials Supplemental Table 1: tissue toxicity of pesticides. Supplemental Table 2: signaling pathways altered by pesticides. Supplemental Table 3: effects of commonly used conventional pesticide active ingredients in the agricultural market sector in 2012 on oxidative stress in different tissues. Supplemental Table 4: effects of most commonly used organophosphate insecticide active ingredients in the home and in 2012 on oxidative stress in different tissues. Supplemental Table 5: table showing additional pesticides that have been documented to cause oxidative stress in different tissues. Supplemental Table 6: table showing pesticide tolerable and nontoxic concentrations.
