# Supplementary material for: A Common Feature of Pesticides: Oxidative Stress—The Role of Oxidative Stress in Pesticide-Induced Toxicity
Source: Oxid Med Cell Longev. 2022 Jan 19;2022:5563759. doi: 10.1155/2022/5563759 (PMC8791758; doi:10.1155/2022/5563759)
Supplement: Supplementary Materials — Supplemental Table 1: tissue toxicity of pesticides. Supplemental Table 2: signaling pathways altered by pesticides. Supplemental Table 3: effects of commonly used conventional pesticide active ingredients in the agricultural market sector in 2012 on oxidative stress in different tissues. Supplemental Table 4: effects of most commonly used organophosphate insecticide active ingredients in the home and in 2012 on oxidative stress in different tissues. Supplemental Table 5: table showing additional pesticides that have been documented to cause oxidative stress in different tissues. Supplemental Table 6: table showing pesticide tolerable and nontoxic concentrations. [file 5563759.f1.pdf]

A Common Feature of Pesticides: Oxidative Stress
The Role of Oxidative Stress in Pesticide-induced Toxicity

Rasheed O. Sule[a], Liam Condon[a], and Aldrin V. Gomes[a,b*]

[a] Department of Neurobiology, Physiology and Behavior,
[b] Department of Physiology and Membrane Biology
University of California, Davis, Davis, CA 95616

Supplemental Tables

Supplemental Table 1. Tissue Toxicity of Pesticides

Pesticide	Cardiac Toxicity	Kidney Toxicity	Liver Toxicity	Brain Toxicity	Other Organ Toxicity
Glyphosate	[1, 2] (Zebrafish (*Danio rerio*)*, and* mammals)	[3-5] (rats and common carp (Cyprinus carpio L.))	[5, 6] (rats and field lizard podarcis siculus)	[7] (mice)	
Atrazine	[8, 9] (mice, African clawed frog)	[10-12] (Piaractus mesopotamicus, quail (Coturnix C. coturnix, Caspian kutum, Rutilus frisii kutum)	[10] (Piaractus mesopotamicus)	[13] (rat)	[14] quails (Coturnix Coturnix coturnix)
Metolachlor-S			[15] (rat)		
2,4-dichlorophenoxyacetic acid (2,4-D)	[16, 17] (rats, humans)	[17, 18] (rodents, humans)	[17, 18] (rodents, humans)	[19] (rodents, fish)	
Metam			[20] (rat)		
Acetochlor	[21] (zebrafish larvae)			[22] (zebrafish larvae)	
Chloropicrin					[23] (respiratory, humans)
Chlorothalonil			[24] (zebrafish (Danio rerio)		[25] (Ovaries, mice)
Pendimethalin	[26] (zebrafish)	[27] (rat)	[27-30] (rat, rainbow trout (Oncorhynchus mykiss),		[31] (bone marrow, mouse)

			Nile tilapia (*Oreochromis niloticus*)		
Ethephon		[32, 33] (rats)	[34, 35] (rats)		
Mancozeb		[36] (rats)	[36-38] (rats)	[36, 39] (rats)	[40] (striated muscle, rats) [41] (reproductive organs/rats)
Chlorpyrifos	[42-44] (rabbits, rats)	[45-48] (rats, mice)	[46, 47, 49] (rats, mice)	[45, 50] (rats)	
Propanil		[51] (mice)	[51-54] (rats, mice)		[55] (Thymus/ mice)
Dicamba			[56] (human)		
Trifluralin		[57] (rats)			
Acephate		[58] (in Vitro/renal tubular cells)			[59] (reproductive organs/ male mice)
Paraquat	[60-63] (mice)	[64-67] (rats)	[66, 68-71] (rats, mice, lizards, zebrafish)	[72-77] (rat, human, mice, monkey, freshwater fish bryconamericus iheringii)	[78] (esophagus, human) [79] (stomach, small intestine, testes, rat)
Glufosinate			[80] (fish)	[81, 82] (mice, zebrafish larvae)	
Phorate			[83] (mice)		[83] (testis, mice)
Dicrotophos					[84] (blood, rat)
Dimethoate	[85, 86] (rats)	[87-89] (rats, mice, fish)	[88-93] (rats, mice, fish)	[91, 93, 94] (rats)	
Terbufos	[95] (rats)				

Lindane	[96-98] (rats)	[99-101] (rats, fish)	[99, 100, 102] (rats, fish)	[103, 104] (rats, mice)	
Dichlorodiphenoxy-trichloroethane (DDT)	[105] (mice)		[106] (rats)		
Maneb		[107-109] (rats, mice, fish)	[108, 110] (mice, fish, zebrafish embryos)	[111] (zebrafish embryos)	
Dieldrin		[112] (rat)	[112] (rat)	[113] (human)	
Clothianidin		[114] (fish)		[114] (fish)	
Chlordane			[115] (rats)		[116] (Thymic atrophy, rats)
Pentachlorophenol (PCP)			[117, 118] (rats)		[119] (reproductive organs/fish)
Endosulfan	[120-122] (rats)	[123] (mice)	[123] (mice)	[122, 124, 125] (rats, zebrafish)	
Zineb			[126] (rats)	[126] (rats)	
Rotenone		[127, 128] (rats)	[127, 129, 130] (rats, fish)	[127, 131] (rats, fish)	
Ziram				[132] (zebrafish embryos)	
Methamidophos	[133] (rats)	[134] (rats)	[134] (rats)	[135] (rats)	
Dichlorvos		[136-139] (rats, mice)	[136, 140, 141] (rats, mice)	[142, 143] (rats)	
Tebuconazole	[144, 145] (rats)	[146] (rat)	[145] (zebrafish)		
Triflumuron		[147] (mice)	[147] (mice)		
Acetamiprid		[148, 149] (rats, mice)	[149, 150] (rats)	[151, 152] (rats, mice)	

Thifluzamide		[153] (zebrafish)	[153, 154] (zebrafish)		
Aldicarb	[155] (human)	[155] (human)	[155] (human)		
Propoxur	[44] (rabbits)		[156, 157] (rats)	[158] (rats)	
Methidathion	[159] (rats)		[160, 161] (rats)		
Diazinon	[162, 163] (rats)	[164, 165] (rats, fish)	[165, 166] (rats, fish)	[167, 168] (rats, mice)	
Monocrotophos	[169] (rats)	[170] (fish)	[170] (fish)	[171] (rats)	
Aluminum phosphide	[172] (rats)	[172] (rats)			
Penconazole (triazole)	[173] (rats)	[174] (rats)			
Imidacloprid (IMI)			[175] (fish)		
Fluazifop-p-butyl (FPB)		[176] (rats)	[176] (rats)		
Nitenpyram			[177] (zebrafish)		
Thiacloprid			[178] (rats)		

Supplemental Table 2. Signaling Pathways altered by Pesticides

Pesticide	Signaling Pathways Involved	Reference
Glyphosate	Mitochondrial apoptotic signaling pathway	[179], [180]
Atrazine	NF-κB signaling pathway	[181]
	Apoptosis signaling pathway<br>Nrf2 signaling pathway<br>TNF-α NF-κB signaling pathway	[182]
	Nrf2 signaling pathway	[183]
	TNF-α NF-κB signaling pathway	[184]
	Autophagy and apoptosis pathways	[185]
	ER stress-signaling pathway<br>Mitochondrial apoptosis pathway<br>Autophagy pathway	[186]
2,4-dichlorophenoxyacetic acid (2,4-D)	Apoptosis signaling pathway	[187]
Acetochlor	MAPK signaling pathway	[188]
	Apoptosis signaling pathway	[189]
Chloropicrin	Apoptotic signaling pathways<br>MAPK signaling pathway	[190], [191]
	ER stress-signaling pathways	[192]
Pendimethalin	TNF-α signaling pathway<br>Apoptosis signaling pathway	[27]
Mancozeb	Apoptosis signaling pathway	[193], [194]
	iNOS and NOX4 signaling pathways	[195]
Chlorpyrifos	Apoptosis signaling pathway<br>PKCδ-STAT1 signaling pathway<br>NOX-1 signaling pathways	[196]
	IκB/NF-kB signaling pathway	[197]
	MAPK signaling pathway	[198]
	Apoptosis signaling pathway<br>Increased TLR7 signaling	[199]

**Pesticide**	**Signaling Pathways Involved**	**Reference**
Paraquat	Nrf2 signaling pathway<br>NF-κB signaling pathway	[200]
	Apoptosis signaling pathway<br>NF-κB signaling pathway	[201]
	ER stress–signaling pathways	[202-206]
	PERK signaling pathway	[207]
	Nrf2 signaling pathway	[208]
	Proteasomal and autophagic Pathways	[209]
Permethrin	TLR4-NF-κB signaling pathway	[210]
	Nrf2 signaling pathway<br>TLR4-NF-κB signaling pathway	[211]
	Nrf2 signaling pathway<br>NF-κB signaling pathway	[212]
Deltamethrin	Nrf2 signaling pathway<br>NF-κB signaling pathway<br>Nrf2 signaling pathway	[212],<br>[213]
Bifenthrin	MAPK signaling pathway	[214]
	TNF-α signaling pathway<br>Flt signaling pathway<br>IL-6 signaling pathway	[215]

Flt, vascular endothelial growth factor receptor 1 precursor; IL-6, interleukin 6; Nrf2, nuclear factor erythroid 2-related factor 2; TLR4, toll Like receptor 4; ER, endoplasmic recticulum; NF-κB, nuclear factor kappa-light-chain-enhancer of activated B cells; MAPK, mitogen-activated protein kinase; TNF-α, tumor necrosis factor alpha; STAT1, signal transducer and activator of transcription 1; NOX-1, nadph oxidase 1

Supplemental Table 3. Effects of commonly used Conventional Pesticide Active Ingredients in the Agricultural Market Sector in 2012 on oxidative stress in different tissues.

Pesticide	Cell Type/Model System	Concentration /dose	Oxidative stress markers	Reference
Glyphosate	Human Skin Keratinocytes HaCaT cells	*10, 25, 50, 100* µM	Increased intracellular ROS levels. Decreased SOD1 expression level.	[179]
	Human liver carcinoma (HepG2) cells	0.5, 2.91, 3.5 µg/mL	Decreased total antioxidant capacity, decreased GPx activity, increased DNA damage.	[216]
	Rat heart H9c2 cells	5 µM, 10 µM	Induced apoptotic cell death. Decreased Bcl-1 expression, increased Bax expression, increased caspases 3/7/9 activity, decreased mitochondrial membrane potential.	[180]
	Adult albino male rats (Liver)	134.95 mg/kg	Increased lipid peroxidation, increased NO level, decreased GSH level, increased TNF-α level.	[217]
	Caenorhabditis elegans	2.7%, 5.5%, 9.8%	Increased GST expression, decreased ATP levels, decreased mitochondrial membrane potential.	[218]
	Chlorella kessleri	40, 50, 60, 70 mg/L	Increased lipid peroxidation, increased GSH content, increased SOD and CAT activities.	[219]
Atrazine (ATR)	Male and female Balb/c mice	100-400 mg/kg	Increased ROS, increased GSSG activity, and decreased GSH activity.	[181]
	Male mice (Liver and kidney)	78.25 mg/kg	Decreased CAT activity, GST activity, and SOD activity. Decreased intracellular ferric reducing/ antioxidant power. Increased lipid peroxidation.	[220]
	Male Wistar rats (Erythrocytes)	300 mg/kg	Decreased GSH activity. Increased SOD activity, CAT activity, increased GPx activity, and GST activity.	[221]
	Adult male Wistar rats (testes and epididymis)	120, 200 mg/kg	Decreased epididymal CAT activity, decreased GST activity, decreased SOD activity, and increased lipid peroxidation.	[222]
	Male Wistar rats	25 mg/kg	Decreased CAT activity, GSH activity, and SOD activity. Increased lipid peroxidation.	[223]
	Adult male albino rats	300 mg/kg	Decreased CAT activity, GPx activity, GSH activity, SOD activity, and increased lipid peroxidation.	[182]
	Female Wistar rats	5 mg/kg and 125 mg/kg	Decreased CAT activity, decreased GPx activity, increased lipid peroxidation, increased NO level, increased expression of Nrf2 in kidney, decreased expression of HO1 and NQO1.	[183]
	Murine microglial cells (BV-2)	12.5, 25, and 50 µM	Increased intracellular ROS, increased NO and iNOS level, increased *TNF-α and IL*-1β level.	[184]

	Albino rats	400 mg/kg	Significantly decreased serum/cardiac tissue GSH and TSH levels; significantly increased cardiac tissue HO-1 activity; serum/cardiac tissue GPx and CAT activity, MDA and serum 8-OHdG level altered; significantly decreased cardiac tissue complex I activity.	[224]
Metolachlor-S	*Scenedesmus obliquus (green algae)*	0.1, 0.2, and 0.3 mg/L	Increased ROS generation level, increased SOD and CAT activities.	[225]
	Parachlorella kessleri (microalga)	2–200 μg/L	Increased intracellular ROS level, increased antioxidant enzymes (APX, GR, CAT) activities, increased lipid peroxidation.	[226]
	Wheat (*Triticum aestivum* L.)	2.5, 5 mg/L	Increased superoxide anion (ROS) level, increased lipid peroxidation, increased SOD activity, decreased POD activity, decreased CAT and DHAR expression.	[227]
2,4-dichlorophenoxyacetic acid (2,4-D)	*Umbelopsis isabelline (Fungus)*	100 mg/L	Increased ROS and RNS level, increased lipid peroxidation, decreased CAT activity.	[228]
	Pea (*Pisum sativum* L.)	45.2 μM<br>22.6 mM	Increased XOD activity, increased SOD, CAT, GPx, APX, and glutathione reductase (GR) activities, increased lipid peroxidation.	[229]
	Non-green potato tuber callus	1-50 μM	Increased CAT, SOD, GR, and GST activities.	[230]
	Male 7-week-old Kunming mice	50, 100, 200 mg/kg	Increased testicular lipid peroxidation, decreased SOD and CAT activities, increased testicular cell apoptosis.	[187]
	Goldfish gills, *Carassius auratus*	1-100 mg/L	Increased lipid peroxidation, increased GSSG content, increased SOD, CAT, and GPx activities.	[231]
	Cnesterodon decemmaculatus	252 mg/L	Increased DNA damage, increased CAT, GST, and GSH activities.	[232]
	Acanthospermum hispidum D.C., Asteraceae weed	300 μM	Increased lipid peroxidation.	[233]
	Rat Cerebellar Granule Cells	1 mM	Increased ROS generation level, decreased GSH level, decreased CAT activity, increased GPx activity	[234]
	Wistar rats	600 ppm	Increased hepatic lipid peroxidation, decreased CAT, SOD, GPx, and GSH activities.	[235]

	Wistar Albino rats	5 mg/kg	Increased hepatic lipid peroxidation, increased protein carbonylation, decreased GSH content. Decreased SOD, CAT, GST, and GPx activities in liver.	[236]
	Male Wistar rats (Liver)	15, 75, 150 mg/kg	Increased lipid peroxidation, decreased CAT, GPx, and GR activities, increased/decreased SOD activity.	[237]
	Male Wistar Albino rats Plasma, Liver, Kidney, Erythrocytes	15, 75, 150 mg/kg	Increased lipid peroxidation, decreased SOD, GPx, and CAT activities.	[238]
	Male Wistar rats (Liver)	15, 75, 150 mg/kg	Decreased CAT and GR enzymatic activities.	[239]
Metam	Female B6C3F1 mice *Peritoneal macrophages*	50 µL	Decreased GSH level, upregulation of genes involved in response to oxidant stress.	[240]
Acetochlor	*Soil bacteria*	62 mM and 620 mM	Increased lipid peroxidation, , increased CAT activity at low concentration, decreased CAT activity at high concentration, decreased GR activity.	[241]
	Bufo raddei tadpole liver	0.017, 0.034 and 0.068 mg/L	Increased lipid peroxidation, increased DNA damage, decreased total antioxidant capability level.	[242]
	Female zebrafish	1-100 µg/L	Increased plasma ROS level, increased plasma CAT, SOD, and GPx activities.	[243]
	Male C57BL/6 mice (testis)	250, 500, and 1000 mg/kg	Increased lipid peroxidation, decreased SOD activity, decreased GSH activity.	[188]
	GC-1 spermatogonia cell	10^{-4} M, 2 × 10^{-4} M, 4 × 10^{-4} M, 8 × 10^{-4} M, and 10^{-3} M	Increased cell apoptosis, increased lipid peroxidation, decreased SOD and GSH activities, increased Bax expression and decreased Bcl-2 expression	[188]
	Human liver carcinoma cells (HepG2)	100-400 µM	Increased ROS generation, increased apoptotic cells, decreased antioxidant SOD and GPx activities, decreased mitochondrial transmembrane potential.	[244]
	Zebrafish	50, 100, 200 µg/L	Increased C*AT, GPX, GPX1a, Cu/Zn-SOD and Mn-SOD mRNA levels.*	[189]

Chloropicrin	Primary human corneal epithelial (HCE) cells	50, 75 μM	Increased apoptotic cell death and DNA damage, increased HO-1 expression, increased lipid peroxidation, increased protein carbonylation.	[190]
	Human retinal pigment epithelial cells (ARPE-19)	1–50 μM	Increased ROS production, increased HO-1 level.	[192]
	Human lung epithelial cells (A549)	1–200 μM	Increased ROS production, increased protein oxidation	[191]
Chlorothalonil	*Gill tissues of Pacific oyster (Crassostrea gigas), blue mussels (Mytilus edulis)*	0.1, 1, and 10 μg/L	Increased lipid peroxidation, increased intracellular GSH level, increased CAT, SOD, GPx, and GR activities.	[245]
	Polychaete Laeonereis acuta	0.1, 10, and 100 μg/L	Decreased antioxidant capacity against peroxyl radicals, increased lipid peroxidation.	[246]
	Fish Danio rerio	0.1 and 10 μg/L	Gills: Increased ROS level, increased antioxidant capacity against peroxyl radicals (ACAP), increased GST and GCL activities,<br>Liver: Increased lipid peroxidation, increased SOD activity.	[247]
	Isolated rat hepatocytes	1–1000 μM	Increased lipid peroxidation, decreased GSH content.	[248]
	Botryllus schlosseri hemocytes	0.1, 1 and 10 μM	Increased apoptotic cells, decreased cytochrome c oxidase activity, decreased GSH activity.	[249]
	Male Wistar rats (Liver)	0.1, 0.13, 0.5, 1 mg/kg	Increased 8-OH-2-deoxyguanosine (8-OH-2-DG) level.	[250]
Pendimethalin	Fresh water fish, *Channa punctatus*	0.9, 1.8, and 2.7 mg/L	Increased lipid peroxidation, decreased GSH level, decreased SOD and CAT activities.	[251]
	Male Wistar rats (Liver and Kidney)	62.5, 125 and 250 mg/kg	Increased lipid peroxidation, increased protein carbonylation, decreased GSH level, decreased SOD, CAT, and GST activities, increased oxidative DNA damage.	[27]
	Human lymphocytes	50, 100, 200 μM	Increased ROS level,<br>increased mitochondrial dysfunction, increased apoptosis, increased oxidative DNA damage.	[252]
	Rat bone-marrow cells	12.5, 25, and 50 mg/kg	Increased oxidative DNA damage, increased ROS level, increased lipid peroxidation, increased mitochondrial dysfunction, increased apoptotic cells, decreased CAT activity, decreased GSH level.	[252]

	Clarias batrachus (liver)	0.177, 0.236, 0.355 mg/L	Increased lipid peroxidation, increased SOD and CAT activities.	[253]
	Fish *Channa punctatus* (Brain)	0.5, 0.8 ppb	Increased lipid peroxidation, increased protein carbonylation, decreased GSH level, decreased CAT, GST, and SOD activities.	[254]
	Fish *Channa punctatus* (Gills, Liver, Kidney)	0.5, 0.8 ppb	Increased lipid peroxidation, increased protein carbonylation, decreased GSH and NP-SH level, decreased CAT, GPx, and GST activities.	[255]
	Chinese hamster lung fibroblast (V79) cells	1, 2.5, 5, 7.5, 10, 25, 50, 100, 250, 500 and 1000 μM	Increased ROS level, increased oxidative DNA damage.	[256]
Ethephon	Male mice (Spleen and thymus)	1995 ppm (1/10th oral LD50)	Increased lipid peroxidation, decreased GSH level, decreased CAT and GPx activities, no change in SOD activity.	[257]
	3T3 murine embryonic fibroblast (MEF) cells	10, 40, 160, 640 μM	Increased ROS production level, increased lipid peroxidation, increased DNA damage.	[258]
	Spinach (Spinacia oleracea L.)	10 mM	Increased CAT, polyphenol oxidase (PPO), peroxidase (POD) activities.	[259]
	Ipomoea cairica (Linn.) Sweet	1.4, 7.2 g/L	Increased H_2O_2 levels.	[260]
	Schisandra chinensis Turcz. (*Baill*.)	1 ppm, 10 ppm	Increased POD activity, decreased PPO activity.	[261]
Mancozeb	*Carassius auratus Goldfish blood and gills*	3, 5 and 10 mg/L	Increased protein carbonylation, increased lipid peroxidation, increased SOD, CAT, GST, and G6PDH activities.	[262]
	Carassius auratus Goldfish brain, liver, and kidney	3, 5 and 10 mg/L	Increased protein carbonylation, increased lipid peroxidation, increased SOD activity in liver and kidney, increased CAT activity in liver, increased GPx activity in liver and kidney.	[263]
	Cassia angustifolia	0.1, 0.15, 0.2 and 0.25 %	Increased lipid peroxidation, increased APX, GR, SOD, CAT activities, increased GSH content.	[264]
	Caenorhabditis elegans	0.1%, 1.0%,1.5%	Increased H_2O_2 levels, increased GST expression, decreased mitochondrial membrane potential, decreased ATP levels.	[265]
	Caenorhabditis elegans	0.1%, 7.5%, 15%	Increased H_2O_2 levels, increased hydroxyl radicals (·OH) level, decreased mitochondrial membrane potential, increased GST expression.	[266]

	Drosophila melanogaster	5 and 10 mg/mL	Increased lipid peroxidation, increased ROS level, increased CAT and GST activities, decreased SOD activity, decreased GSH level.	[267]
	Rat-1 fibroblasts, Peripheral blood mononucleated cells (PBMC)	125, 250, 500 ng/mL	Increased 8-hydroxy-2′-deoxyguanosine (8-OHdG) level, increased ROS level, increased apoptotic cells.	[193]
	Male NMRI mice	50 and 500 mg/kg	Increased lipid peroxidation, increased protein carbonylation, decreased SOD and CAT activities, decreased GSH content, decreased total antioxidant capacity, decreased GPx expression, increased iNOS and NOX4 expression.	[195]
	Rat thymocytes	0.5, 2 and 5 µg/ml	Increased ROS production, increased apoptotic cells, decreased mitochondrial membrane potential, decreased ATP levels.	[268]
	Human Gastric Adenocarcinoma (*AGS*) cells	5, 10 µM	Increased ROS level, increased apoptotic cells, decreased mitochondrial transmembrane potential, upregulation of Bax and downregulation of Bcl-2 and Bcl-xL.	[194]
Chlorpyrifos	Immortalized murine mesencephalic dopaminergic (N27) cells	300 nM-300 µM	Increased intracellular ROS generation, increased mitochondrial ROS, decreased mitochondrial membrane potential, decreased GSH level, increased DNA fragmentation, increased caspase-3 activation and proteolytic cleavage of PARP, increased Bax level and decreased Bcl-2 level, increased proteolytic cleavage of PKCδ, increased STAT1 phosphorylation.	[196]
	Lund human mesencephalic (LUHMES) cells	10 nM-100 µM	Increased DNA fragmentation, increased intracellular ROS, decreased mitochondrial membrane potential, and increased caspase-3 activity.	[196]
	Human neuroblastoma *SH-SY5Y* cells	50 µM to 200 µM	Increased intracellular ROS, increased apoptosis, increased TNF-α levels, dysfunction in HO-1 and MnSOD levels.	[197]
	Rat adrenal pheochromocytoma (PC12) cells	25, 50, 100, and 200 µM	Increased apoptotic cells, increased activity of caspase-9 and caspase-3 and cleaved of PARP-1, increased ROS level, increased lipid peroxidation, decreased mitochondrial complex I activity, increased HO-1 level, decreased CuZnSOD and MnSOD levels.	[198]
	Rat erythrocytes	6.75 mg/kg	Decreased SOD activity, GST activity, and CAT activity. Increased lipid peroxidation.	[269]
	Male Wistar rats	4.75 mg/kg	Decreased CAT activity, decreased SOD activity, increased GPx activity, and increased lipid peroxidation.	[270]

	Male Wistar rats (aorta, liver, plasma, and kidney)	2.5, 5, 25 mg/kg	Increased lipid peroxidation, increased nitration (NO), increased SOD activity.	[271]
	Male Swiss albino adult rats	5, 10, 20, 30 mg/kg	Increased lipid peroxidation, decreased testicular CAT, SOD, and GPx activities, decreased GSH content.	[272]
	Adult male Wistar rats	8 mg/kg	Increased amygdala ROS levels. Increased amygdala and hippocampal NO level.	[273]
	Male Wistar rats	5.4 mg/kg	Increased levels of MDA, SOD and CAT; decreased GPx and GST activities in heart.	[274]
	Male Kunming mice	10^{-5} M	Increased lipid peroxidation, increased •OH level, decreased SOD, GPx, and CAT activities, decreased GSH content, increased levels of Bax, Bcl-2, and p53 in splenic B cells.	[199]
Metolachlor	Male Wistar rats (Liver)	400–1000 nmol/mg	Decreased mitochondrial membrane potential.	[15]
	Soil bacteria	34 mM and 340 mM	Increased lipid peroxidation, increased CAT activity at low concentration, decreased CAT activity at high concentration, decreased GR activity.	[241]
	Lettuce, bean and pea seeds and leaves	0.2 – 200 μM	Decreased CAT, SOD, and GPx antioxidant enzymatic activities.	[275]
Propanil	8 weeks old male rats	200 mg/kg	Increased lipid peroxidation, decreased SOD, GPx, and GST activities, decreased GSH level.	[276]
	Wistar rats Liver	100 mg/kg	Increased lipid peroxidation, decreased CAT, SOD, GST, and GSH activities.	[277]
	Albino rats Liver	20 mg/kg	Increased lipid peroxidation, decreased GSH level, decreased CAT activity.	[52]
	Common carp (*Cyprinus carpio*) Brain	2 and 4 mg/L	Increased lipid peroxidation, increased protein carbonylation, decreased GSH level, decreased SOD, CAT activities	[278]
Dicamba	Isolated mitochondria from Potato tubers (*Solanum tuberosum)*	5-30 μmol/mg	Decreased mitochondrial transmembrane potential, inhibited complex II, III, and IV activity.	[279]
	Non-green potato tuber callus	1-50 μM	Increased CAT, SOD, GR, and GST activities.	[230]
	Cnesterodon decemmaculatus	410 mg/L	Increased DNA damage, increased CAT, GST, and GSH activities.	[232]

	Isolated mitochondria Arabidopsis	1 mM	Increased mitochondrial H_2O_2 levels.	[280]
Trifluralin	Chinese hamster lung fibroblast (V79) cells	1, 2.5, 5, 7.5, 10, 25, 50, 100, 250, 500 and 1000 µM	Increased ROS level, increased oxidative DNA damage.	[256]
	Male Wistar albino rats Kidney, ureter, urinary bladder	0.8, 2 g/kg	Increased lipid peroxidation, decreased SOD and GPx activities, increased apoptotic DNA fragmentation.	[57]
Acephate	*Chlamydomonas mexicana*	15 mg/L	Increased SOD and CAT activities.	[281]
	Drosophila melanogaster	1-6 µg/mL	Increased lipid peroxidation, increased protein carbonylation, increased CYP450, SOD, CAT, and GST activities, increased Hsp70 expression, increased DNA damage.	[282]
	Male albino rats (Plasma and Liver)	30 mg/kg	Increased lipid peroxidation, decreased CAT, SOD, and GSH activities, increased GPx activity.	[283]
	Male rats (Erythrocytes)	360 mg/kg	Increased lipid peroxidation, decreased GSH content, increased GST and GR activities.	[284]
	Albino rats	50 mg/kg	Increased lipid peroxidation, decreased testicular activities of CAT, SOD, and levels of GSH.	[285]
	Human Sperm	50, 100, and 200 µg/mL	Increased DNA damage.	[286]
	Chinese hamster ovary (CHO-K1) cells	237.12 µM	Increased GST and GPx activities, increased GSH content.	[287]
	Porcine kidney proximal tubule *cell* line (*LLC-PK)*	2500 ppm	Increased lipid peroxidation, increased H_2O_2 accumulation,	[58]
Paraquat (PQ)	Human dopaminergic neuroblastoma cells (SK-N-SH)	0.2, 0.5, 1 mM	Increased cytosolic ROS, increased mitochondrial ROS, increased apoptosis, increased oxidative stress in the mitochondrial matrix, decreased mitochondrial membrane potential, increased free radical formation, increased lipid peroxidation.	[200]
	Human IMR-32 neuroblastoma cells	0.05, 0.1, 0.2 mM	Transcriptional activity driven by ARE and NF-κB activated.	[200]
	Rat lung slices	100 µM–10 mM	Complex I-dependent increased ROS production.	[288]
	Rat organotypic midbrain slice cultures	10–50 µM	Excitotoxic mechanism of ROS production.	[289]
	Rat primary mesencephalic cultures	0.5–1 µM	NADPH oxidase-dependent microglial ROS production.	[290]

	Rat primary mesencephalic cultures	250 μM	Robust and immediate production of ROS, involvement of mitochondrial complex III and mitochondrial membrane potential in ROS generation.	[291]
	Human neural progenitor cells (hNPCs)	1, 10, 100 μM	Decreased SOD and CAT, increased lipid peroxidation, increased Nrf2, HO-1, and NQO1expression, upregulated Cyt C and caspase-9.	[208]
	Human neural progenitor cells (hNPCs)	0.1, 1, 10, 100 μM	Increased apoptotic cells, increased intracellular ROS, increased caspase-3 activity, increased NF-κB activity, p53 and p21 mRNA expression.	[201]
	Human plasma	unknown	Increased lipid peroxidation, decreased plasma antioxidant capacity and plasma SH groups.	[292]
	Rat brain mitochondria	250 μM	Robust and immediate production of ROS, mitochondrial uptake of PQ, involvement of mitochondrial complex III and mitochondrial membrane potential in ROS generation.	[293]
	Non-green potato tuber callus	1-50 μM	Increased CAT, SOD, and GR activities.	[230]
Glufosinate	*Amaranthus palmeri*	560 g/ha	Increased O_2^- and H_2O_2 (ROS) level, increased SOD, CAT, APX, and GR activities.	[294]
	Horseweed, palmer amaranth, kochia	560 g/ha	Increased lipid peroxidation, increased O_2^- and H_2O_2 (ROS) level, increased SOD, CAT, APX, and GR activities.	[295]
	Chlorella vulgaris	10, 20 μg/mL	Increased lipid peroxidation, increased SOD, CAT, and POD activities.	[296]

Supplemental Table 4. Effects of most commonly used Organophosphate Insecticide Active Ingredients in the Home and in 2012 on oxidative stress in different tissues.

Pesticide	Cell Type/Model System	Concentration/dose	Oxidative stress markers	Reference
Phorate	Male Wistar rats (Liver/bone marrow cells)	0.046, 0.092, 0.184 mg/kg	Increased lipid peroxidation, decreased CAT activity and GSH level, increased intracellular ROS in bone marrow cells, reduced mitochondrial membrane potential in bone marrow, significant cellular DNA damage and apoptosis.	[297]
	Human amnion epithelial (WISH) cells	50-1000 µM	Increased extracellular superoxide anion (O_2^-) production, increased intracellular ROS generation, mitochondrial damage.	[298]
	Human lymphocytes	50-500 µM	Increased intracellular ROS generation, decreased mitochondrial membrane potential.	[299]
	Calf thymus DNA (ct-DNA)	1000 µM	Increased 8-Hydroxy-2′-deoxyguanosine (8-oxodG) DNA adduct formation.	[299]
Dicrotophos	Renal tubular epithelial cell (LLC-PK_1)	1250 ppm	Increased ROS production, increased lipid peroxidation.	[300]
	Human liver carcinoma (HepG2) cells	25 - 400 µM	Increased DNA damage level.	[301]
	Human liver carcinoma (HepG2) cells	50 µM–200 µM	Increased intracellular ROS production, decreased mitochondrial membrane potential, increased apoptosis.	[302]
Dimethoate	Bok choy	600 g a.i. /ha (applied dose of active ingredient per hectare)	Increased ROS level.	[303]
	Rat liver and kidney	*10-30 mg/kg*	Decreased SOD level, increased lipid peroxidation.	[304]
	Rat peripheral blood cells	*10-60 mg/kg*	Increased DNA damage and fragmentation, increased apoptosis, decreased mitochondrial membrane potential.	[305]
	Human peripheral blood lymphocytes	40-100 mM	Increased lipid peroxidation, increased protein oxidation, increased SOD and CAT activities.	[306]
	Gammarus pulex	10-40 µg/L	Increased lipid peroxidation, decreased GSH level, decreased SOD, CAT, and GST activities.	[307]

	Male Wistar rat liver and brain	0.6, 6, 30 mg/kg	Increased lipid peroxidation, increased SOD, CAT, GPx, and GR activities, decreased GSH at 30 mg/kg.	[91]
	Adult female rats	0.2 g/L	Increased lipid peroxidation, decreased GPx, SOD, CAT activities activity, decreased GSH level.	[308]
	Female Wistar rats (Erythrocytes)	0.2 g/L	Increased lipid peroxidation, increased protein carbonylation, increased advanced oxidation protein products (AOPP), increased CAT, SOD, and GPx activities decreased GSH level.	[309]
	Rat erythrocytes	0.03 mg/kg	Increased lipid peroxidation, increased SOD and CAT activities, increased total-SH content.	[310]
	Freshwater fish *Channa punctatus* (Bloch)	4.8, 9.6, 14.3 μg/L	Increased lipid peroxidation, decreased SOD activity, increased GSH level, increased DNA damage.	[311]
	Oncorhynchus mykiss	0.0735, 0.3675, 0.7350 mg/L	Increased lipid peroxidation, increased SOD and GPx activities.	[312]
	Cyprinus carpio	16 and 32 μg/L	Increased lipid peroxidation, increased CAT activity, decreased total antioxidant levels.	[313]
Terbufos	Mouse immortalized spermatogonia (GC-1) cells	50, 100, 200 μM	Increased intracellular ROS production, decreased mitochondrial membrane potential, increased apoptosis, increased DNA damage.	[314]

Supplemental Table 5. Table showing additional pesticides that have been documented to cause oxidative stress in different tissues.

Pesticide	Cell Type/Model System	Concentration/dose	Oxidative stress markers	Reference
Lindane	Thymic cells from C57BL/6 mice	37.5, 50, 75, 150, 200 μM	PER and lindane mixtures increased SOD activity, had no effect on CAT levels and inhibited GPx and GSH-R specific activities.	[315]
	Rats	100 mg/kg	Increased lipid peroxidation, decreased GSH, SOD, CAT, GPx, and GST activities.	[96]
Dichlorodiphenoxy-trichloroethane (DDT)	Human normal liver (HL-7702) cells	*p,p'*-DDT (10, 20, 30 μM)	Increased intracellular ROS. Activated caspase-9, increased Bax and p53 levels, anti-apoptotic gene Bcl-2 downregulated, and increased NF-κB p65 levels.	[316]
Maneb	Rat adrenal pheochromocytoma (PC12) cells	50–1000 ng/ml	Increased GSH and GSSG, altered HO-1 levels.	[317]
	Rat primary mesencephalic cultures	30 μM	ROS generation, mitochondrial uncoupling.	[318]
		10–120 μM	Decreased ATP levels, mitochondrial dysfunction.	[318]
Dieldrin	Rat adrenal pheochromocytoma (PC12) cells	30–500 μM	Increased ROS, decreased mitochondrial membrane potential and activity, decreased lipid peroxidation, increased DNA fragmentation.	[319]
	Rat adrenal pheochromocytoma (PC12) cells	100μM	Increased 8-oxodGuo levels (created by ROS).	[320]
	Rat adrenal pheochromocytoma (PC12) cells	100-300μM	Increased caspase-3 activity and DNA fragmentation.	[321]
	Immortalized mouse nigral clonal (SN4741) cells	40 μM	Increased ROS, apoptotic cell death	[322]
	PC12 cells	30-100 μM	Increased ROS, apoptotic cell death.	[323]
	Immortalized rat mesencephalic clonal (N27) cells	0-70μM	α-synuclein aggregation, increased DNA fragmentation.	[324]
	Primary mouse microglial cultures	0.1 nM–1 μM	Microglial NADPH-oxidase dependent ROS production.	[325]

	Human THP-1 monocytes	10 *μ*M, 20 *μ*M	Elevated ROS level, dose-dependent increase in $p47^{phox}$ phosphorylation (necessary to activate NOX2).	[326]
	Mice	3–48 mg/kg	Increased lipid peroxidation.	[327]
	Mice	0.3–3 mg/kg	Decreased GSH, decreased DAT expression, increased α-synuclein, increased cysteinyl-catechol adducts.	[328]
Clothianidin	Male Wister rats	2, 8, and 24 mg/kg	Increased TBARS significantly in testicular tissue, GSH level did not change significantly in all treated groups.	[329]
	Male Wister rats	2, 8, and 32 mg/kg	Significant decrease in the GSH level in the testicular tissue.	[330]
Chlordane	Human THP-1 monocytes	1 *μ*M , 5 *μ*M, and 10 *μ*M *trans*-nonachlor	Elevated ROS level, enhanced superoxide production, dose-dependent increase in $p47^{phox}$ phosphorylation (necessary to activate NOX2).	[326]
	Murine macrophage cell J774a.1	10 *μ*M *trans*-nonachlor	Increased intracellular superoxide level	[326]
	Human HL-60 cell	1 *μ*M and 10 *μ*M *trans*-nonachlor	Increased intracellular superoxide level	[326]
Pentachlorophenol (PCP)	Primary murine splenocytes	25–100 μM PCP	Increased ROS production, increased apoptosis, decreased mitochondrial membrane potential.	[331]
	Primary murine splenocytes	PCP metabolite (12.5–50 μM TCHQ)	Significantly increased ROS production, increased apoptosis, increase in annexin V and PI positive (necrotic cells), decreased mitochondrial membrane potential, ERK activation, increased expression of cleaved caspase-3 and degradation of PARP.	[331]
	Murine hepatocytes	0.03, 0.06, and 0.12%	Increased 8-OHdG levels	[332]
	Sprague–Dawley rats	40 mg/kg PCP	Increased lipid peroxidation	[118]
Endosulfan	Human peripheral blood lymphocytes	0.1, 17 μM	Increased apoptosis	[333]
	Human neuroblastoma *SH-SY5Y* cells	50, 100, 200 μM	Increased intracellular ROS, increased intracellular superoxide anion, decreased specific activities of SOD, CAT, and GPX,	[334]

			increased lipid peroxidation, increased expression of NFκB p50.	
	Male Wistar rats	2 mg/kg	Increased SOD, GPx, CAT activities and increased (MDA level) lipid peroxidation.	[122]
Zineb	Human neuroblastoma *SH-SY5Y* cells	50, 100, 200 μM	Increased intracellular ROS, increased intracellular superoxide anion, decreased specific activities of SOD, CAT, and GPX, increased lipid peroxidation, increased expression of NFκB p50.	[334]
1-methyl-4-phenylpyridinium (MPP^+)	Human dopaminergic neuroblastoma cells (SK-N-SH)	0.01–2.5 mM MPP^+	Increased mitochondrial ROS, increased apoptosis, increased oxidative stress in the mitochondrial matrix, decreased mitochondrial membrane potential, increased lipid peroxidation.	[200]
	Rat dopaminergic N27 cells	100–1000 μM	Increased intracellular ROS, increased Nox2 protein expression.	[335]
Rotenone	Rat primary mesencephalic cultures	0.5–30 nM	NADPH oxidase dependent microglial ROS production.	[336]
	Human dopaminergic neuroblastoma cells (SK-N-SH)	0.65–4 μM	Increase mitochondrial ROS, increased apoptosis, decreased mitochondrial membrane potential, increased lipid peroxidation.	[200]
Ziram	Human Natural Killer *Cells (NK-92MI)*	0.125 – 4 μM	Increased apoptosis, increased intracellular levels of active caspases3, 3/7, 8, 9, increased cytochrome-c release and decreased mitochondrial membrane potential.	[337]
	Progenitor Leydig cells (PLCs)	1 – 5 μM	Increased intracellular ROS, increased apoptosis, decreased mitochondrial membrane potential.	[338]
Methamidophos	Human Peripheral Blood Mononuclear Cells (PBMCs)	3 – 20 mg/L	Increased intracellular ROS production, increased lipid peroxidation, decreased GSH level.	[339]
Dichlorvos	Adult male Wistar rats	8.8 mg/kg	Increased amygdala and hippocampal ROS levels. Increased NO level both in amygdala and hippocampus.	[340]
	Human erythrocytes	1 – 100 μM	Decreased SOD, CAT, and GPx activities. Increased lipid peroxidation.	[341]

	Human colon carcinoma (HCT116) cell	150 – 450 μM	Increased ROS, increased lipid peroxidation, increased DNA damage, decreased mitochondrial membrane potential, increased caspase-3, CAT, and SOD activities.	[342], [343]
	Male Wistar rats (Erythrocytes)	70 mg/kg	Increased lipid peroxidation, increased SOD activity.	[344]
	Male albino rats (Brain)	6 mg/kg	Increased lipid peroxidation, increased protein carbonylation, increased mitochondrial DNA oxidation, increased level of mitochondrial ROS production, decreased mitochondrial glutathione levels and MnSOD activity.	[345]
Tebuconazole	H9c2 cardiac cells	10 – 100 μM	Increased mitochondrial ROS, increased lipid peroxidation, increased apoptosis, decreased mitochondrial membrane potential, increased in Bax/Bcl-2 level, activated caspase-9 and caspase-3.	[346]
	Male Wistar rat	0.9, 9 and 27 mg/kg	Increased lipid peroxidation, protein carbonyl, advanced oxidation protein product (AOPP) levels and DNA damage. Increased renal SOD, CAT, and GPx activities, decreased GSH activity.	[346]
Triflumuron	Balb/c mice bone marrow cell	250 – 500 mg/kg	Increased DNA damage.	[347]
	Human colon carcinoma (HCT116) cell	100, 200, 400 and 600 μM	Increased intracellular ROS, increased lipid peroxidation, increased DNA damage, increased SOD, CAT, and GST activities. Decreased mitochondrial membrane potential	[347], [348]
	Human renal embryonic cells (HEK 293) and Human liver carcinoma cells (HepG2)	50 – 300 μM	Increased intracellular ROS, increased lipid peroxidation, increased Hsp70 levels, increased CAT and SOD activities, decreased mitochondrial membrane potential, increased expression levels of Bax and decreased expression of Bcl-2.	[349]
	Male Balb/C mice	250, 350, and 500 mg/kg	Increased protein carbonyl, increased lipid peroxidation, increased CAT, SOD, GPx, and GST activities.	[147]
Acetamiprid	Rat adrenal pheochromocytoma (PC12) cells	110, 220, and 330 μM	Increased intracellular ROS, increased lipid peroxidation, increased DNA damage, decreased mitochondrial membrane potential.	[350]

	Pancreatic Cell Line AR42J	1 - 6 mM	Increased DNA damage, decreased GSH level.	[351]
	Male and female Wistar rats (Kidney & Liver)	5.5, 11, and 22 mg/kg	Increased lipid peroxidation, decreased GSH, SOD, CAT, and GPx activities.	[352]
	Kumming male mice	30 mg/kg	Increased MDA and NO concentrations in the testes; reduced the activity of CAT, GPx, SOD; and activated p38.	[353]
Thifluzamide	Zebrafish embryos	0.19, 1.9, 2.85 mg/L	Increased lipid peroxidation, decreased SOD.	[354]
Aldicarb	Chinese Hamster Ovary (CHO-K1) cells	Aldicarb 10 µg/mL	Increased lipid peroxidation, decreased GSH activity, increased GR, GPx and GST activities.	[355]
	Chinese Hamster Ovary (CHO-K1) cells	Aldicarb sulfone 10 µg/mL	Increased lipid peroxidation, decreased GSH activity, increased GR, GPx and GST activities.	[355]
	Chinese Hamster Ovary (CHO-K1) cells	Aldicarb sulfoxide 10 µg/mL	Increased lipid peroxidation, decreased GSH activity, increased GR, GPx and GST activities.	[355]
Propoxur	Chinese Hamster Ovary (CHO-K1) cells	10 µg/mL	Increased lipid peroxidation, decreased GSH activity, increased GR, GPx and GST activities.	[355]
Methidathion	Male Wistar rats	5 mg/kg	Increased lipid peroxidation.	[356]
Diazinon	Female Sprague-Dawley rats	8, 10, 12 and 20 mg/kg	Increased lipid peroxidation, decreased SOD, GPx activities.	[357]
	Male Wistar albino rats	20 mg/kg	A significant increase in cardiac MDA and NO content was observed compared with the control group, but cardiac antioxidants were significantly ($p \leq .05$) decreased.	[358]
	Male MFI mice	16.25 and 32.5 mg/kg	Increased lipid peroxidation, increased protein carbonylation, decreased GSH level, decreased CAT, SOD, GPx, and GR activities.	[359]
	Rats	15 mg/kg	Increased MDA level, lower level of reduced GSH, induction of apoptosis	[163]
	Male Wistar rats	20 mg/kg	Increased level of TNF-α and *8-iso-prostaglandin F*2α	[360]
	Wistar albino rats	335 mg/kg	Increased lipid peroxidation	[361]

Monocrotophos	Wistar rats	0.36 mg/kg	Cardiac oxidative stress was conferred by accumulation of protein carbonyls, lipid peroxidation and glutathione production.	[169]
	Male albino Wistar rats (Liver, Kidney, Spleen, Brain)	4.5 and 9.0 mg/kg	Increased lipid peroxidation, increased DNA damage.	[362]
	Chinese hamster ovary (CHO-K1) cells	233.58 μM	Increased GPx activity, increased GSH content.	[287]
Aluminum phosphide	Male Wistar rats	12 mg/kg	Elevated ROS and plasma iron levels. Increased Caspase 3 and 9 levels, increased CAT activity.	[363]
	Male Wistar rats	6 and 12 mg/kg	Increased hydroxyl radicals, increased lipid peroxidation, decreased mitochondrial complexes (II, IV and V), decreased mitochondrial membrane potential, increased apoptosis.	[364]
Penconazole (triazole)	Male Wistar rats	67 mg/kg	Increased lipid peroxidation, protein carbonyls and DNA fragmentation in the heart, increased CAT, SOD, and GPx activities, decreased GSH level.	[173]
Imidacloprid (IMI)	Chinese hamster ovary (CHO-K1) cells	0.97– 500 μM	Significant inhibition of the activity of GST, GPx, and GR.	[365]
	Male Wistar rats	40 mg/kg	Significant increase in MDA level, the activities of CAT, and SOD and decreased the activities of GST and total SH.	[366]
	Male Wistar rats	45 and 90 mg/kg	Significantly increased MDA levels and decreased GSH levels and the activities of CAT, SOD, GPx, and GST.	[367]
	Male Wistar rats	45 and 90 mg/kg	Increased MDA content and decreased the activities of SOD, GSH, GPx, and CAT.	[368]
	Male Wistar rats	0.5, 2, and 8 mg/kg	Increased lipid peroxidation and increased apoptosis.	[369]
	Wistar rats	1 mg/kg	Significant increase in NO production in brain. IMI induced the mRNA transcription of iNOS, eNOS, and nNOS in brain and iNOS and eNOS in the liver.<br>IMI significantly increased CAT activity in brain and increased MDA levels in plasma, brain, and liver. XO activity was elevated in liver and	[370]

			brain; MPO activity was increased only in the liver. GSH levels were significantly depleted in brain.	
	Female Wistar rats	5, 10, and 20 mg/kg	Significantly increased MDA content and significantly decreased SOD, CAT and GPx activities, and GSH content in ovary.	[371]
	Female Wistar rats	10 μM equivalent to 2.6 mg/100 g	Increased the production of NO levels in liver. MDA increased significantly in liver and plasma. CAT, SOD, and GPx activities responded differently to IMI administration. GSH level was significantly decreased in the liver and brain.	[372]
	Female Wistar rats	5, 10, and 20 mg/kg	Significantly decreased GSH level in liver and the activities of GPx, CAT, and SOD in liver and brain and significantly increased MDA content in brain and kidney.	[373]
	Male Swiss albino mice	14.976 mg/kg	Significantly increased MDA levels, increased CAT, SOD, GPx and GST activities, and decreased GSH level.	[374]
	Earthworm *Eisenia fetida*	0.2, 0.66, 2, and 4 mg/kg	IMI significantly increased ROS level, MDA content, and CAT and peroxidase (POD) activities.	[375]
	Honeybees (*Apis mellifera* L.) thorax	0.7, 2.0 ng/mL	Increased lipid peroxidation, increased GPx, CAT activities	[376]
Fluazifop-*p*-butyl (FPB)	Male Wistar rats	18.75, 37.50, and 75.00 mg/kg	Increased lipid peroxidation, decreased testicular antioxidant status, decreased testicular activities of SOD, CAT, GST and GSH.	[176]
	Acanthospermum hispidum	10 μM	Increased lipid peroxidation, increased ROS level,	[377]
	Acanthospermum hispidum	5 μM, 10 μM	Increased ROS level, increased SOD, CAT, APx, and GR activities.	[378]
	Acanthospermum hispidum	5 μM, 10 μM	Increased lipid peroxidation.	[379]
Nitenpyram	Male and female zebrafish (*Danio rerio*)	0.6, 1.2, 2.5, and 5.0 mg/L	Resulted to DNA damage in the exposed zebrafish livers. SOD and CAT activities were inhibited; ROS production, GST activity, DNA damage, and MDA content increased.	[177]

Thiacloprid	Male Wistar rats	22.5, 112.5 mg/kg	Significantly decreased CAT, GPx, and GSH, increased lipid peroxidation levels in lymphoid organs and significantly increased total NOx (NO2 and NO3) in polymorphonuclear leukocytes and TBARS levels in all lymphoid organs and the plasma.	[380]
	Earthworm *Eisenia fetida*	1 and 3 mg/kg	Increased DNA damage, inhibited the activities of GST, CAT, SOD, and POD.	[381]

Supplemental Table 6. Table showing pesticide tolerable and nontoxic concentrations.

Pesticide	Nontoxic concentrations	Reference
Glyphosate	10-100 μM induced proliferation and cell growth in Human Skin Keratinocytes HaCaT cells.	[179]
	0.5-2.91 μg/mL induced a slight increase of cell proliferation after treatment in Human liver carcinoma (HepG2) cells.	[216]
	2.7% No significant superoxide and hydroxyl radicals production in *Caenorhabditis elegans*.	[218]
	0.5 - 15 mM had no effects on transmembrane potential (Δψm), no effect on enzymatic activities of mitochondrial respiratory complexes II, III, and IV, and no effect on ATP and ATPsynthase activities in isolated rat liver mitochondria.	[382]
Atrazine (ATR)	5 and 25 mg/kg had no significant increase on blood urea nitrogen (BUN) and creatinine (CREA) levels in female Wistar rats.	[183]
Metolachlor-S	2 μg/L had no effect on cell growth and cell numbers at 48h treatment, it decreased ROS content at 72 h treatment, and decreased lipid peroxidation at 4 h treatment in *Parachlorella kessleri* (microalga).	[226]
2,4-dichlorophenoxyacetic acid (2,4-D	0.01-0.5μM had no effect on cell growth in non-green potato tuber callus.	[230]
	1-100 mg/L had no effect on protein carbonyl levels in Goldfish gills, *Carassius auratus*.	[231]
	15 mg/kg had no clinical signs of 2,4-D poisoning in Male Wistar rats (Liver)	[237]
	15 mg/kg had no clinical signs of 2,4-D poisoning in Male Wistar rats (Liver)	[239]
Acetochlor	0.017mg/L had no significant increase in lipid peroxidation (MDA content) in tadpole liver.	[242]
	250mg had no effect in the organ/body ratio in lung, spleen, testis in C57BL/6 male mice.	[188]

	50 μM did not induce any significant cytotoxic effect for 12 h and 24 h in HepG2 cells. 100 μM had no effect on the mitochondrial membrane potential after 24 hr treatment.	[244]
Chloropicrin	10-20 μM had no significant decrease in cell viability of human retinal pigment epithelial (ARPE-19) cells.	[192]
	10 μM had no significant decrease in cell viability of lung epithelial cell line (A549).	[191]
Chlorothalonil	1-5 μM did not induce cytotoxicity (<15% LDH leakage) and lipid peroxidation in isolated rat hepatocytes.	[248]
Pendimethalin	50 μM no effect on mitochondrial membrane potential (Δψm) in human lymphocytes.	[252]
	12.5 mg/kg did not show significant ROS level and had no significant effect on the mitochondrial membrane potential in rat bone-marrow cells.	[252]
	1-25 μM had no effect on cell viability in Chinese hamster lung fibroblast V79 cells.	[256]
Ethephon	10-160 μg/ml significantly increased cell proliferation in 3T3 embryonic fibroblast cells. 10-40 μg/ml had no significant changes in ROS content and lipid peroxidation.	[258]
	3-10 mg/L had no effect on significant effect on the protein carbonylation. 3-5 mg/L had no effect on lipid peroxidation in *Carassius auratus.*	[262]
Mancozeb	5mg/ml had no significant effect on cell viability after 15 days of treatment in Drosophila melanogaster.	[267]
	50mg/kg had no effect on lipid peroxidation in male NMRI mice testes.	[195]
Chlorpyrifos	25 μM had no significant effect on cell viability in SH-SY5Y cells.	[197]
	25 μM had no significant effect on cell viability in PC12 cells and had no effect on cell morphology.	[198]
	2.5 mg/kg had lower MDA level compared to 5 and 10mg/kg exposure in aorta, liver, plasma, and kidney of male Wistar rats.	[271]
	0.1 and 1 μM did not affect cell viability in splenocytes.	[199]

Metolachlor	100 μM did not affect cell density in *B. stearothermophilus.*	[15]
Paraquat	0.1-10 μM did not affect cell viability in human neural progenitor cells.	[201]
Bifenthrin	5 μM did not affect cell viability in human colorectal HCT-116 cells.	[214]
	5mg/kg had no effect on the body weight of male mice and no obvious signs of poisoning was found.	[383]
	1-5 μM did not affect cell viability in human umbilical vein endothelial (HUVEC) cells.	[215]
Malathion	6 mM had no effect on cell viability and had a mitogenic effect on the growth of human liver carcinoma (HepG2) cells.	[384]
Phorate	0.046mg/kg did not induce lipid peroxidation in rats liver tissue after 14 days exposure.	[297]
Dimethoate	0.6mg/kg did not induce lipid peroxidation in rats liver and brain tissue.	[91]
Endosulfan	50 μM had no significant difference in LDH release (cell viability) in SH-SY5Y cells	[334]
Zineb	50 μM had no significant difference in LDH release (cell viability) in SH-SY5Y cells.	[334]
Methamidophos	3 mg/L did not affect the cell viability in human peripheral blood mononuclear cells.	[339]
Dichlorvos	1 μM did not induce MDA level/lipid peroxidation in erythrocytes.	[341]
Acetamiprid	27.5-55 mg/kg had no clinical signs of toxicity in Wistar rats. No significant decreases in body weight during 13 weeks of treatment.	[352]
Imidacloprid	5-10 mg/kg did not produce any signs of toxicity and mortality during 90 day exposure.	[371]
	0.2 mg/kg had no significant change in ROS level and MDA content during exposure period of 14 days in *Eisenia fetida*.	[375]

References:

1. Gress, S., et al., *Glyphosate-based herbicides potently affect cardiovascular system in mammals: review of the literature.* Cardiovasc Toxicol, 2015. **15**(2): p. 117-26.
2. Roy, N.M., et al., *Glyphosate induces cardiovascular toxicity in Danio rerio.* Environ Toxicol Pharmacol, 2016. **46**: p. 292-300.
3. Gunatilake, S., S. Seneff, and L. Orlando, *Glyphosate's Synergistic Toxicity in Combination with Other Factors as a Cause of Chronic Kidney Disease of Unknown Origin.* Int J Environ Res Public Health, 2019. **16**(15).
4. Ma, J., Y. Bu, and X. Li, *Immunological and histopathological responses of the kidney of common carp (Cyprinus carpio L.) sublethally exposed to glyphosate.* Environ Toxicol Pharmacol, 2015. **39**(1): p. 1-8.
5. Tizhe, E.V., et al., *Effect of zinc supplementation on chronic hepatorenal toxicity following oral exposure to glyphosate-based herbicide (Bushfire(R)) in rats.* J Int Med Res, 2020. **48**(8): p. 300060520925343.
6. Verderame, M. and R. Scudiero, *How Glyphosate Impairs Liver Condition in the Field Lizard Podarcis siculus (Rafinesque-Schmaltz, 1810): Histological and Molecular Evidence.* Biomed Res Int, 2019. **2019**: p. 4746283.
7. Gallegos, C.E., et al., *Intranasal glyphosate-based herbicide administration alters the redox balance and the cholinergic system in the mouse brain.* Neurotoxicology, 2020. **77**: p. 205-215.
8. Asouzu Johnson, J., et al., *Cardiotoxicity in African clawed frog (Xenopus laevis) sub-chronically exposed to environmentally relevant atrazine concentrations: Implications for species survival.* Aquat Toxicol, 2019. **213**: p. 105218.
9. Lin, J., et al., *The chemopreventive potential of lycopene against atrazine-induced cardiotoxicity: modulation of ionic homeostasis.* Sci Rep, 2016. **6**: p. 24855.
10. de Paiva, P.P., et al., *Acute toxicity of commercial atrazine in Piaractus mesopotamicus: Histopathological, ultrastructural, molecular, and genotoxic evaluation.* Vet World, 2017. **10**(9): p. 1008-1019.
11. Zhang, C., et al., *Atrazine induced oxidative stress and mitochondrial dysfunction in quail (Coturnix C. coturnix) kidney via modulating Nrf2 signaling pathway.* Chemosphere, 2018. **212**: p. 974-982.
12. Khoshnood, Z., *Histopathological alterations in the kidney of Caspian kutum, Rutilus frisii kutum, larvae and fingerlings exposed to sublethal concentration of atrazine.* Bull Environ Contam Toxicol, 2015. **94**(2): p. 158-63.
13. Filipov, N.M., et al., *Dopaminergic toxicity of the herbicide atrazine in rat striatal slices.* Toxicology, 2007. **232**(1-2): p. 68-78.
14. Lin, J., et al., *A novel mechanism underlies atrazine toxicity in quails (Coturnix Coturnix coturnix): triggering ionic disorder via disruption of ATPases.* Oncotarget, 2016. **7**(51): p. 83880-83892.
15. Pereira, S.P., et al., *Toxicity assessment of the herbicide metolachlor comparative effects on bacterial and mitochondrial model systems.* Toxicology in Vitro, 2009. **23**(8): p. 1585-1590.
16. De Oliveira Mantovani, R., et al., *Effect of different doses of 2,4-dichlorophenoxyacetic acid (2,4-D) on cardiac parameters in male Wistar rats.* Environ Sci Pollut Res Int, 2021. **28**(3): p. 3078-3087.

17. Singh, S., et al., *Fatal 2,4-D (ethyl ester) ingestion.* J Assoc Physicians India, 2003. **51**: p. 609-10.
18. Garabrant, D.H. and M.A. Philbert, *Review of 2,4-dichlorophenoxyacetic acid (2,4-D) epidemiology and toxicology.* Crit Rev Toxicol, 2002. **32**(4): p. 233-57.
19. Freitas, L.M., et al., *Animal models in the neurotoxicology of 2,4-D.* Hum Exp Toxicol, 2019. **38**(10): p. 1178-1182.
20. Thompson, R.W., H.L. Valentine, and W.M. Valentine, *In Vivo and in Vitro Hepatotoxicity and Glutathione Interactions of N-Methyldithiocarbamate and N,N-Dimethyldithiocarbamate in the Rat.* Toxicological Sciences, 2002. **70**(2): p. 269-280.
21. Liu, H., et al., *In vivo cardiovascular toxicity induced by acetochlor in zebrafish larvae.* Chemosphere, 2017. **181**: p. 600-608.
22. Yang, M., et al., *Thyroid endocrine disruption of acetochlor on zebrafish (Danio rerio) larvae.* J Appl Toxicol, 2016. **36**(6): p. 844-52.
23. Pesonen, M. and K. Vahakangas, *Chloropicrin-induced toxicity in the respiratory system.* Toxicol Lett, 2020. **323**: p. 10-18.
24. Sanchez Garayzar, A.B., et al., *Hepatic gene expression profiling in zebrafish (Danio rerio) exposed to the fungicide chlorothalonil.* Comp Biochem Physiol Part D Genomics Proteomics, 2016. **19**: p. 102-111.
25. Hao, Y., et al., *Chlorothalonil inhibits mouse ovarian development through endocrine disruption.* Toxicol Lett, 2019. **303**: p. 38-47.
26. Meng, Y., et al., *Cardiac toxicity assessment of pendimethalin in zebrafish embryos.* Ecotoxicology and Environmental Safety, 2021. **222**: p. 112514.
27. Ahmad, M.I., et al., *Pendimethalin-induced oxidative stress, DNA damage and activation of anti-inflammatory and apoptotic markers in male rats.* Scientific Reports, 2018. **8**(1): p. 17139.
28. Danion, M., et al., *Effects of in vivo chronic exposure to pendimethalin on EROD activity and antioxidant defenses in rainbow trout (Oncorhynchus mykiss).* Ecotoxicology and Environmental Safety, 2014. **99**: p. 21-27.
29. Hamed, H.S. and Y.S. El-Sayed, *Antioxidant activities of Moringa oleifera leaf extract against pendimethalin-induced oxidative stress and genotoxicity in Nile tilapia, Oreochromis niloticus (L.).* Fish Physiology and Biochemistry, 2019. **45**(1): p. 71-82.
30. El-Sayed, Y.S., et al., *Physiological and oxidative stress biomarkers in the freshwater monosex Nile tilapia, Oreochromis niloticus L., exposed to pendimethalin-based herbicide.* Environmental Toxicology, 2015. **30**(4): p. 430-438.
31. Dimitrov, B.D., et al., *Comparative genotoxicity of the herbicides Roundup, Stomp and Reglone in plant and mammalian test systems.* Mutagenesis, 2006. **21**(6): p. 375-382.
32. Tousson, E., et al., *Modulatory effects of Saussurea lappa root aqueous extract against ethephon-induced kidney toxicity in male rats.* Environ Toxicol, 2019. **34**(12): p. 1277-1284.
33. Mokhtari, T., et al., *Ameliorative effect of virgin olive oil against nephrotoxicity following sub-chronic administration of ethephon in male rats.* J Tradit Complement Med, 2020. **10**(5): p. 487-495.
34. Al-Brakati, A., *Protective effect of aged garlic extracts against hepatotoxicity induced by ethephon in Wistar albino rat.* Environ Sci Pollut Res Int, 2020. **27**(6): p. 6139-6147.
35. Bhadoria, P., et al., *Effect of ethephon on the liver in albino rats: A histomorphometric study.* Biomed J, 2015. **38**(5): p. 421-7.

36. Kackar, R., M.K. Srivastava, and R.B. Raizada, *Assessment of toxicological effects of mancozeb in male rats after chronic exposure.* Indian J Exp Biol, 1999. **37**(6): p. 553-9.
37. Saber, T.M., A.M.A. Abo-Elmaaty, and H.M. Abdel-Ghany, *Curcumin mitigates mancozeb-induced hepatotoxicity and genotoxicity in rats.* Ecotoxicol Environ Saf, 2019. **183**: p. 109467.
38. Yahia, E., et al., *Subchronic Mancozeb Treatement Induced Liver Toxicity Via Oxidative Stress in Male Wistar Rats.* Commun Agric Appl Biol Sci, 2014. **79**(3): p. 553-9.
39. Tsang, M.M. and L.D. Trombetta, *The protective role of chelators and antioxidants on mancozeb-induced toxicity in rat hippocampal astrocytes.* Toxicol Ind Health, 2007. **23**(8): p. 459-70.
40. Stephenson, O.J. and L.D. Trombetta, *Comparative effects of Mancozeb and Disulfiram-induced striated muscle myopathies in Long-Evans rats.* Environ Toxicol Pharmacol, 2020. **74**: p. 103300.
41. Girish, B.P. and P.S. Reddy, *Forskolin ameliorates mancozeb-induced testicular and epididymal toxicity in Wistar rats by reducing oxidative toxicity and by stimulating steroidogenesis.* J Biochem Mol Toxicol, 2018. **32**(2).
42. Cetin, N., et al., *Chlorpyrifos induces cardiac dysfunction in rabbits.* Res Vet Sci, 2007. **82**(3): p. 405-8.
43. Khalaf, H.A. and A.A.E. El-Mansy, *The possible alleviating effect of saffron on chlorpyrifos experimentally induced cardiotoxicity: Histological, immunohistochemical and biochemical study.* Acta Histochem, 2019. **121**(4): p. 472-483.
44. Zafiropoulos, A., et al., *Cardiotoxicity in rabbits after a low-level exposure to diazinon, propoxur, and chlorpyrifos.* Hum Exp Toxicol, 2014. **33**(12): p. 1241-52.
45. Nasr, H.M., F.M. El-Demerdash, and W.A. El-Nagar, *Neuro and renal toxicity induced by chlorpyrifos and abamectin in rats: Toxicity of insecticide mixture.* Environ Sci Pollut Res Int, 2016. **23**(2): p. 1852-9.
46. Kucukler, S., et al., *Hesperidin protects against the chlorpyrifos-induced chronic hepato-renal toxicity in rats associated with oxidative stress, inflammation, apoptosis, autophagy, and up-regulation of PARP-1/VEGF.* Environ Toxicol, 2021. **36**(8): p. 1600-1617.
47. Ma, P., et al., *Oxidative damage induced by chlorpyrifos in the hepatic and renal tissue of Kunming mice and the antioxidant role of vitamin E.* Food Chem Toxicol, 2013. **58**: p. 177-83.
48. Baba, N., et al., *Free radical-induced nephrotoxicity following repeated oral exposureto chlorpyrifos alone and in conjunction with fluoride in rats.* Turkesh Journal of Medical Science, 2016. **46**(2): p. 512-517.
49. Sharma, S., et al., *Toxicity assessment of chlorpyrifos on different organs of rat: exploitation of microbial-based enzymatic system for neutralization.* Environ Sci Pollut Res Int, 2019. **26**(29): p. 29649-29659.
50. Kopjar, N., et al., *Evaluation of chlorpyrifos toxicity through a 28-day study: Cholinesterase activity, oxidative stress responses, parent compound/metabolite levels, and primary DNA damage in blood and brain tissue of adult male Wistar rats.* Chem Biol Interact, 2018. **279**: p. 51-63.
51. Cakici, O. and E. Akat, *Propanil-induced histopathological changes in the liver and kidney of mice.* Anal Quant Cytopathol Histpathol, 2013. **35**(3): p. 163-70.

52. Otuechere, C.A., et al., *Protective Effect of Curcumin against the Liver Toxicity Caused by Propanil in Rats.* International Scholarly Research Notices, 2014. **2014**: p. 853697.
53. Otuechere, C.A. and E.O. Farombi, *Pterocarpus mildbraedii leaf extract ebbs propanil-induced oxidative and apoptotic damage in the liver of rats.* Drug Chem Toxicol, 2020: p. 1-8.
54. Santillo, M., et al., *Enhancement of tissue lipoperoxidation in propanil-treated rats.* Toxicol Lett, 1995. **78**(3): p. 215-8.
55. Cuff, C.F., et al., *3,4-Dichloropropionanilide-induced atrophy of the thymus: mechanisms of toxicity and recovery.* Fundam Appl Toxicol, 1996. **33**(1): p. 83-90.
56. Lerro, C.C., et al., *Dicamba use and cancer incidence in the agricultural health study: an updated analysis.* International journal of epidemiology, 2020. **49**(4): p. 1326-1337.
57. Denek, Z., et al., *The effects of resveratrol against trifluralin toxicity in the urinary tract of rats.* Toxicology and Industrial Health, 2016. **32**(1): p. 106-117.
58. Poovala, V.S., et al., *Role of Oxidant Stress and Antioxidant Protection in Acephate-Induced Renal Tubular Cytotoxicity.* Toxicological Sciences, 1998. **46**(2): p. 403-409.
59. Farag, A.T., M.H. Eweidah, and A.M. El-Okazy, *Reproductive toxicology of acephate in male mice.* Reprod Toxicol, 2000. **14**(5): p. 457-62.
60. Li, Q., et al., *Insulin-like growth factor I deficiency prolongs survival and antagonizes paraquat-induced cardiomyocyte dysfunction: role of oxidative stress.* Rejuvenation Res, 2007. **10**(4): p. 501-12.
61. Ge, W., et al., *Cardiac-specific overexpression of catalase attenuates paraquat-induced myocardial geometric and contractile alteration: role of ER stress.* Free Radic Biol Med, 2010. **49**(12): p. 2068-77.
62. Wang, S., et al., *Toll-like receptor 4 knockout alleviates paraquat-induced cardiomyocyte contractile dysfunction through an autophagy-dependent mechanism.* Toxicol Lett, 2016. **257**: p. 11-22.
63. Wang, J., et al., *Cardiac-Specific Knockout of ETA Receptor Mitigates Paraquat-Induced Cardiac Contractile Dysfunction.* Cardiovasc Toxicol, 2016. **16**(3): p. 235-43.
64. Silfeler, I., et al., *Protective Effect of Ellagic Acid on Paraquat-induced Kidney Hazards in Rats.* Iran J Kidney Dis, 2017. **11**(1): p. 23-28.
65. Wei, T., et al., *Protective effects of exogenous beta-hydroxybutyrate on paraquat toxicity in rat kidney.* Biochem Biophys Res Commun, 2014. **447**(4): p. 666-71.
66. Awadalla, E.A., *Efficacy of vitamin C against liver and kidney damage induced by paraquat toxicity.* Exp Toxicol Pathol, 2012. **64**(5): p. 431-4.
67. Tan, D., et al., *Betanin attenuates oxidative stress and inflammatory reaction in kidney of paraquat-treated rat.* Food Chem Toxicol, 2015. **78**: p. 141-6.
68. Eftekhari, A., et al., *Hepatoprotective role of berberine against paraquat-induced liver toxicity in rat.* Environ Sci Pollut Res Int, 2020. **27**(5): p. 4969-4975.
69. Muller, T.E., et al., *Sodium Selenite Prevents Paraquat-Induced Neurotoxicity in Zebrafish.* Mol Neurobiol, 2018. **55**(3): p. 1928-1941.
70. Zhang, L., et al., *Hepatotoxicity and reproductive disruption in male lizards (Eremias argus) exposed to glufosinate-ammonium contaminated soil.* Environ Pollut, 2019. **246**: p. 190-197.
71. Yang, H., et al., *LncRNA NR_030777 Alleviates Paraquat-Induced Neurotoxicity by Regulating Zfp326 and Cpne5.* Toxicol Sci, 2020. **178**(1): p. 173-188.

72. Di Marzio, W.D. and M.C. Tortorelli, *Acute toxicity of paraquat and no-inhibitory chronic effect on brain acetylcholinesterase activity of freshwater fish bryconamericus iheringii (pisces, characidae).* Journal of Environmental Science and Health, Part B, 1993. **28**(6): p. 701-709.
73. Naylor, J., et al., *Further evidence that the blood/brain barrier impedes paraquat entry into the brain.* Human & Experimental Toxicology, 1995. **14**(7): p. 587-594.
74. Wu, B., et al., *Central nervous system damage due to acute paraquat poisoning: A neuroimaging study with 3.0T MRI.* NeuroToxicology, 2012. **33**(5): p. 1330-1337.
75. McCormack, A.L., et al., *Role of oxidative stress in paraquat-induced dopaminergic cell degeneration.* Journal of Neurochemistry, 2005. **93**(4): p. 1030-1037.
76. Fei, Q., et al., *Paraquat Neurotoxicity Is Mediated by a Bak-dependent Mechanism*.* Journal of Biological Chemistry, 2008. **283**(6): p. 3357-3364.
77. Bartlett, R.M., et al., *Paraquat is excluded by the blood brain barrier in rhesus macaque: An in vivo pet study.* Brain research, 2009. **1259**: p. 74-79.
78. Chen, H.-H., et al., *Spectrum of corrosive esophageal injury after intentional paraquat or glyphosate-surfactant herbicide ingestion.* International journal of general medicine, 2013. **6**: p. 677-683.
79. Debe, E., B. Okolonkwo, and A. Ngokere, *Toxicological effects of paraquat on the histology of the stomach, small intestine and testis of male albino rat (Rattus norvegicus).* Port Harcourt Medical Journal, 2007. **2**.
80. Mohanty, G., et al., *Application of comet assay in the study of DNA damage and recovery in rohu (Labeo rohita) fingerlings after an exposure to phorate, an organophosphate pesticide.* Ecotoxicology, 2011. **20**(1): p. 283-92.
81. Comakli, S., et al., *Immunofluorescence/fluorescence assessment of brain-derived neurotrophic factor, c-Fos activation, and apoptosis in the brain of zebrafish (Danio rerio) larvae exposed to glufosinate.* Neurotoxicology, 2018. **69**: p. 60-67.
82. Meme, S., et al., *MRI characterization of structural mouse brain changes in response to chronic exposure to the glufosinate ammonium herbicide.* Toxicol Sci, 2009. **111**(2): p. 321-30.
83. Morowati, M., *Inhalation toxicity studies of thimet (phorate) in male Swiss albino mouse, Mus musculus: I. Hepatotoxicity.* Environ Pollut, 1997. **96**(3): p. 283-8.
84. Nurulain, S.M., et al. *Protective effects of the antihistamine promethazine aginst acute paraxon-methyl and dicrotophos toxicity in adult rats*. International journal of clinical and experimental medicine, 2015. **8**, 17891-17901.
85. Amara, I.B., et al., *Protective effects of vitamin E and selenium against dimethoate-induced cardiotoxicity in vivo: biochemical and histological studies.* Environ Toxicol, 2013. **28**(11): p. 630-43.
86. Saafi-Ben Salah, E.B., et al., *Antioxidant-rich date palm fruit extract inhibits oxidative stress and nephrotoxicity induced by dimethoate in rat.* J Physiol Biochem, 2012. **68**(1): p. 47-58.
87. Ben Amara, I., et al., *Dimethoate induces kidney dysfunction, disrupts membrane-bound ATPases and confers cytotoxicity through DNA damage. Protective effects of vitamin E and selenium.* Biol Trace Elem Res, 2013. **156**(1-3): p. 230-42.
88. Ayed-Boussema, I., et al., *Genotoxicity associated with oxidative damage in the liver and kidney of mice exposed to dimethoate subchronic intoxication.* Environ Sci Pollut Res Int, 2012. **19**(2): p. 458-66.

89. Gill, T.S., J.C. Pant, and J. Pant, *Gill, liver, and kidney lesions associated with experimental exposures to carbaryl and dimethoate in the fish (Puntius conchonius Ham.).* Bull Environ Contam Toxicol, 1988. **41**(1): p. 71-8.
90. Yan, X., et al., *Effects of ZnO Nanoparticles on Dimethoate-Induced Toxicity in Mice.* J Agric Food Chem, 2015. **63**(37): p. 8292-8.
91. Sharma, Y., et al., *Dimethoate-induced effects on antioxidant status of liver and brain of rats following subchronic exposure.* Toxicology, 2005. **215**(3): p. 173-181.
92. Saafi, E.B., et al., *Protective effect of date palm fruit extract (Phoenix dactylifera L.) on dimethoate induced-oxidative stress in rat liver.* Exp Toxicol Pathol, 2011. **63**(5): p. 433-41.
93. Yahia, D. and M.F. Ali, *Assessment of neurohepatic DNA damage in male Sprague-Dawley rats exposed to organophosphates and pyrethroid insecticides.* Environ Sci Pollut Res Int, 2018. **25**(16): p. 15616-15629.
94. Arnal, N., et al., *Pro-apoptotic effects of low doses of dimethoate in rat brain.* Toxicol Appl Pharmacol, 2019. **363**: p. 57-63.
95. Nurulain, S.M., et al., *Terbufos-sulfone exacerbates cardiac lesions in diabetic rats: a sub-acute toxicity study.* Arh Hig Rada Toksikol, 2016. **67**(2): p. 126-35.
96. Vijaya Padma, V., et al., *Oral treatment with gallic acid and quercetin alleviates lindane-induced cardiotoxicity in rats.* Can J Physiol Pharmacol, 2013. **91**(2): p. 134-40.
97. Sauviat, M.P. and N. Pages, *[Cardiotoxicity of lindane, a gamma isomer of hexachlorocyclohexane].* J Soc Biol, 2002. **196**(4): p. 339-48.
98. Ananya, R., et al., *Oxidative stress and histopathological changes in the heart following oral lindane (gamma hexachlorohexane) administration in rats.* Med Sci Monit, 2005. **11**(9): p. BR325-9.
99. Bijoy Nandan, S. and P.J. Nimila, *Lindane toxicity: histopathological, behavioural and biochemical changes in Etroplus maculatus (Bloch, 1795).* Mar Environ Res, 2012. **76**: p. 63-70.
100. Vijaya Padma, V., et al., *Protective effect of gallic acid against lindane induced toxicity in experimental rats.* Food Chem Toxicol, 2011. **49**(4): p. 991-8.
101. Prasad, W.L., et al., *Amelioration of Gamma-hexachlorocyclohexane (Lindane) induced renal toxicity by Camellia sinensis in Wistar rats.* Vet World, 2016. **9**(11): p. 1331-1337.
102. Padma, V.V., et al., *Effect of quercetin against lindane induced alterations in the serum and hepatic tissue lipids in wistar rats.* Asian Pac J Trop Biomed, 2012. **2**(11): p. 910-5.
103. Bist, R., S. Misra, and D.K. Bhatt, *Inhibition of lindane-induced toxicity using alpha-lipoic acid and vitamin E in the brain of Mus musculus.* Protoplasma, 2010. **242**(1-4): p. 49-53.
104. Sharma, P. and R. Singh, *Dichlorvos and lindane induced oxidative stress in rat brain: Protective effects of ginger.* Pharmacognosy Res, 2012. **4**(1): p. 27-32.
105. La Merrill, M.A., et al., *Perinatal DDT Exposure Induces Hypertension and Cardiac Hypertrophy in Adult Mice.* Environ Health Perspect, 2016. **124**(11): p. 1722-1727.
106. Harada, T., et al., *Toxicity and Carcinogenicity of Dichlorodiphenyltrichloroethane (DDT).* Toxicol Res, 2016. **32**(1): p. 21-33.
107. Jaballi, I., et al., *Polysaccharide from a Tunisian red seaweed Chondrus canaliculatus: Structural characteristics, antioxidant activity and in vivo hemato-nephroprotective properties on maneb induced toxicity.* Int J Biol Macromol, 2019. **123**: p. 1267-1277.

108. Boran, H., I. Altinok, and E. Capkin, *Histopathological changes induced by maneb and carbaryl on some tissues of rainbow trout, Oncorhynchus mykiss.* Tissue Cell, 2010. **42**(3): p. 158-64.
109. Jaballi, I., et al., *Increasing maneb doses induces reactive oxygen species overproduction and nephrotoxicity in adult mice.* Toxicol Mech Methods, 2017. **27**(5): p. 382-393.
110. Sefi, M., et al., *Beneficial role of vanillin, a polyphenolic flavoring agent, on maneb-induced oxidative stress, DNA damage, and liver histological changes in Swiss albino mice.* Hum Exp Toxicol, 2019. **38**(6): p. 619-631.
111. Cao, F., et al., *Developmental neurotoxicity of maneb: Notochord defects, mitochondrial dysfunction and hypoactivity in zebrafish (Danio rerio) embryos and larvae.* Ecotoxicol Environ Saf, 2019. **170**: p. 227-237.
112. Hfaiedh, N., et al., *Protective effects of fenugreek (Trigonella foenum graecum L.) upon dieldrin-induced toxicity in male rat.* Gen Physiol Biophys, 2012. **31**(4): p. 423-30.
113. Kanthasamy, A.G., et al., *Dieldrin-induced neurotoxicity: relevance to Parkinson's disease pathogenesis.* Neurotoxicology, 2005. **26**(4): p. 701-19.
114. Dogan, D., G. Nur, and H.A. Deveci, *Tissue-specific toxicity of clothianidin on rainbow trout (Oncorhynchus mykiss).* Drug Chem Toxicol, 2021: p. 1-11.
115. Ogata, M. and F. Izushi, *Effects of chlordane on parameters of liver and muscle toxicity in man and experimental animals.* Toxicol Lett, 1991. **56**(3): p. 327-37.
116. Bondy, G., et al., *Toxicity of the chlordane metabolite oxychlordane in female rats: clinical and histopathological changes.* Food Chem Toxicol, 2003. **41**(2): p. 291-301.
117. Bekhouche, K., et al., *Hepatoprotective effects of the n-butanol extract from Perralderia coronopifolia Coss. against PCP-induced toxicity in Wistar albino rats.* Environ Sci Pollut Res Int, 2019. **26**(30): p. 31215-31224.
118. Wang, Y.-J., et al., *Oxidative stress and liver toxicity in rats and human hepatoma cell line induced by pentachlorophenol and its major metabolite tetrachlorohydroquinone.* Toxicology Letters, 2001. **122**(2): p. 157-169.
119. Yang, L., J. Zha, and Z. Wang, *Pentachlorophenol affected both reproductive and interrenal systems: In silico and in vivo evidence.* Chemosphere, 2017. **166**: p. 174-183.
120. Wei, J., et al., *Endosulfan induces cardiotoxicity through apoptosis via unbalance of pro-survival and mitochondrial-mediated apoptotic pathways.* Sci Total Environ, 2020. **727**: p. 138790.
121. Wei, J., et al., *Endosulfan induces cell dysfunction through cycle arrest resulting from DNA damage and DNA damage response signaling pathways.* Sci Total Environ, 2017. **589**: p. 97-106.
122. Kalender, S., et al., *Endosulfan-induced cardiotoxicity and free radical metabolism in rats: the protective effect of vitamin E.* Toxicology, 2004. **202**(3): p. 227-235.
123. Yan, J., et al., *Discrepant effects of alpha-endosulfan, beta-endosulfan, and endosulfan sulfate on oxidative stress and energy metabolism in the livers and kidneys of mice.* Chemosphere, 2018. **205**: p. 223-233.
124. Pereira, V.M., et al., *Endosulfan exposure inhibits brain AChE activity and impairs swimming performance in adult zebrafish (Danio rerio).* Neurotoxicology, 2012. **33**(3): p. 469-75.
125. Lakroun, Z., et al., *Oxidative stress and brain mitochondria swelling induced by endosulfan and protective role of quercetin in rat.* Environ Sci Pollut Res Int, 2015. **22**(10): p. 7776-81.

126. Astiz, M., M.J. de Alaniz, and C.A. Marra, *Effect of pesticides on cell survival in liver and brain rat tissues.* Ecotoxicol Environ Saf, 2009. **72**(7): p. 2025-32.
127. Abdel-Salam, O.M.E., et al., *Bougainvillea spectabilis flowers extract protects against the rotenone-induced toxicity.* Asian Pac J Trop Med, 2017. **10**(5): p. 478-490.
128. Jiang, X.W., et al., *Rotenone induces nephrotoxicity in rats: oxidative damage and apoptosis.* Toxicol Mech Methods, 2017. **27**(7): p. 528-536.
129. Melo, K.M., et al., *Short-term exposure to low doses of rotenone induces developmental, biochemical, behavioral, and histological changes in fish.* Environ Sci Pollut Res Int, 2015. **22**(18): p. 13926-38.
130. Jiang, X., et al., *The effects of rotenone-induced toxicity via the NF-kappaB-iNOS pathway in rat liver.* Toxicol Mech Methods, 2017. **27**(4): p. 318-325.
131. Badruzzaman, M., et al., *Rotenone alters behavior and reproductive functions of freshwater catfish, Mystus cavasius, through deficits of dopaminergic neurons in the brain.* Chemosphere, 2021. **263**: p. 128355.
132. Lulla, A., et al., *Neurotoxicity of the Parkinson Disease-Associated Pesticide Ziram Is Synuclein-Dependent in Zebrafish Embryos.* Environ Health Perspect, 2016. **124**(11): p. 1766-1775.
133. Maretto, G.X., et al., *Acute exposure to the insecticide O,S-dimethyl phosphoramidothioate (methamidophos) leads to impairment of cardiovascular reflexes in rats.* Ecotoxicol Environ Saf, 2012. **80**: p. 203-7.
134. Araoud, M., et al., *Toxic effects of methamidophos on paraoxonase 1 activity and on rat kidney and liver and ameliorating effects of alpha-tocopherol.* Environ Toxicol, 2016. **31**(7): p. 842-54.
135. Kalkan, S., et al., *Protective effect of an adenosine A1 receptor agonist against metamidophos-induced toxicity and brain oxidative stress.* Toxicol Mech Methods, 2009. **19**(2): p. 148-53.
136. Somia el, M. and F. Madiha, *Pathological effects of dichlorvos and fenitrothion in mice.* Pathol Res Pract, 2012. **208**(5): p. 286-91.
137. Agarwal, S., B. Chaudhary, and R. Bist, *Protective propensity of bacoside A and bromelain on renal cholinesterases, gamma-Aminobutyric acid and serotonin level of Mus musculus intoxicated with dichlorvos.* Chem Biol Interact, 2017. **261**: p. 139-144.
138. Hou, Y., et al., *Effect of quercetin against dichlorvos induced nephrotoxicity in rats.* Exp Toxicol Pathol, 2014. **66**(4): p. 211-8.
139. Celik, M.M., et al., *Protective Effects of Intralipid and Caffeic Acid Phenethyl Ester on Nephrotoxicity Caused by Dichlorvos in Rats.* J Anal Methods Chem, 2015. **2015**: p. 491406.
140. Binukumar, B.K., et al., *Mitochondrial energy metabolism impairment and liver dysfunction following chronic exposure to dichlorvos.* Toxicology, 2010. **270**(2-3): p. 77-84.
141. Guo, R., et al., *[The protective effect of regulation of paraoxonase 1 gene on liver oxidative stress injury induced by dichlorvos poisoning in mice].* Zhonghua Wei Zhong Bing Ji Jiu Yi Xue, 2015. **27**(4): p. 285-90.
142. Choudhary, S. and K.D. Gill, *Protective effect of nimodipine on dichlorvos-induced delayed neurotoxicity in rat brain(1).* Biochem Pharmacol, 2001. **62**(9): p. 1265-72.

143. Kaur, P., et al., *Impaired mitochondrial energy metabolism and neuronal apoptotic cell death after chronic dichlorvos (OP) exposure in rat brain.* Neurotoxicology, 2007. **28**(6): p. 1208-19.
144. Ben Othmene, Y., et al., *Tebuconazole induced cardiotoxicity in male adult rat.* Food Chem Toxicol, 2020. **137**: p. 111134.
145. Othmene, Y.B., et al., *Tebuconazole induced oxidative stress and histopathological alterations in adult rat heart.* Pestic Biochem Physiol, 2020. **170**: p. 104671.
146. Othmene, Y.B., et al., *Oxidative stress, DNA damage and apoptosis induced by tebuconazole in the kidney of male Wistar rat.* Chem Biol Interact, 2020. **330**: p. 109114.
147. Timoumi, R., et al., *Acute triflumuron exposure induces oxidative stress responses in liver and kidney of Balb/C mice.* Environ Sci Pollut Res Int, 2019. **26**(4): p. 3723-3730.
148. Erdemli, M.E., et al., *Protective effects of melatonin and vitamin E in acetamiprid-induced nephrotoxicity.* Environ Sci Pollut Res Int, 2020. **27**(9): p. 9202-9213.
149. Karaca, B.U., et al., *Toxic effects of subchronic oral acetamiprid exposure in rats.* Toxicol Ind Health, 2019. **35**(11-12): p. 679-687.
150. Chakroun, S., et al., *Hematological, biochemical, and toxicopathic effects of subchronic acetamiprid toxicity in Wistar rats.* Environ Sci Pollut Res Int, 2016. **23**(24): p. 25191-25199.
151. Kagawa, N. and T. Nagao, *Neurodevelopmental toxicity in the mouse neocortex following prenatal exposure to acetamiprid.* J Appl Toxicol, 2018. **38**(12): p. 1521-1528.
152. Dhouib, I.B., et al., *Neuroprotective effects of curcumin against acetamiprid-induced neurotoxicity and oxidative stress in the developing male rat cerebellum: biochemical, histological, and behavioral changes.* Environ Sci Pollut Res Int, 2017. **24**(35): p. 27515-27524.
153. Yang, Y., et al., *Toxic effects of thifluzamide on zebrafish (Danio rerio).* J Hazard Mater, 2016. **307**: p. 127-36.
154. Yang, Y., et al., *Crosstalk of oxidative damage, apoptosis, and autophagy under endoplasmic reticulum (ER) stress involved in thifluzamide-induced liver damage in zebrafish (Danio rerio).* Environ Pollut, 2018. **243**(Pt B): p. 1904-1911.
155. Proença, P., et al., *Aldicarb poisoning: one case report.* Forensic Science International, 2004. **146**: p. S79-S81.
156. Wang, P., et al., *Subchronic toxicity of low dose propoxur, permethrin, and their combination on the redox status of rat liver.* Chem Biol Interact, 2017. **272**: p. 21-27.
157. Liang, Y.J., et al., *(1)H NMR-based metabonomic profiling of rat serum and urine to characterize the subacute effects of carbamate insecticide propoxur.* Biomarkers, 2012. **17**(6): p. 566-74.
158. Mehta, K.D., et al., *Protective effect of melatonin on propoxur-induced impairment of memory and oxidative stress in rats.* Environ Toxicol, 2014. **29**(6): p. 705-13.
159. Yavuz, T., et al., *Cardiotoxicity in rats induced by methidathion and ameliorating effect of vitamins E and C.* Hum Exp Toxicol, 2004. **23**(7): p. 323-9.
160. Sutcu, R., et al., *The effects of subchronic methidathion toxicity on rat liver: role of antioxidant vitamins C and E.* Cell Biol Toxicol, 2006. **22**(3): p. 221-7.
161. Gokalp, O., et al., *The effects of methidathion on liver: role of vitamins E and C.* Toxicol Ind Health, 2003. **19**(2-6): p. 63-7.

162. Danaei, G.H., et al., *Protective effect of thymoquinone, the main component of Nigella Sativa, against diazinon cardio-toxicity in rats.* Drug Chem Toxicol, 2019. **42**(6): p. 585-591.
163. Razavi, B.M., et al., *Protective effect of crocin on diazinon induced cardiotoxicity in rats in subchronic exposure.* Chem Biol Interact, 2013. **203**(3): p. 547-55.
164. Boroushaki, M.T., et al., *Protective effect of pomegranate seed oil against acute toxicity of diazinon in rat kidney.* Iran J Pharm Res, 2013. **12**(4): p. 821-7.
165. Al-Otaibi, A.M., et al., *Toxicity bioassay and sub-lethal effects of diazinon on blood profile and histology of liver, gills and kidney of catfish, Clarias gariepinus.* Braz J Biol, 2019. **79**(2): p. 326-336.
166. Ahmadi-Naji, R., E. Heidarian, and K. Ghatreh-Samani, *Evaluation of the effects of the hydroalcoholic extract of Terminalia chebula fruits on diazinon-induced liver toxicity and oxidative stress in rats.* Avicenna J Phytomed, 2017. **7**(5): p. 454-466.
167. Yilmaz, N., M. Yilmaz, and I. Altuntas, *Diazinon-induced brain toxicity and protection by vitamins E plus C.* Toxicol Ind Health, 2012. **28**(1): p. 51-7.
168. Sonei, A., et al., *Protective Effects of Berberis vulgaris on Diazinon-Induced Brain Damage in Young Male Mice.* Prev Nutr Food Sci, 2020. **25**(1): p. 65-70.
169. Velmurugan, G., D.D. Venkatesh Babu, and S. Ramasamy, *Prolonged monocrotophos intake induces cardiac oxidative stress and myocardial damage in rats.* Toxicology, 2013. **307**: p. 103-8.
170. Narra, M.R., et al., *In vivo impact of monocrotophos on biochemical parameters of a freshwater fish during subacute toxicity and following cessation of exposure to the insecticide.* Z Naturforsch C J Biosci, 2011. **66**(9-10): p. 507-14.
171. Karumuri, S.B., et al., *Impact of chronic low dose exposure of monocrotophos in rat brain: Oxidative/ nitrosative stress, neuronal changes and cholinesterase activity.* Toxicol Rep, 2019. **6**: p. 1295-1303.
172. Haghi-Aminjan, H., et al., *The role of minocycline in alleviating aluminum phosphide-induced cardiac hemodynamic and renal toxicity.* Environ Toxicol Pharmacol, 2018. **64**: p. 26-40.
173. Chaâbane, M., et al., *Improvement of Heart Redox States Contributes to the Beneficial Effects of Selenium Against Penconazole-Induced Cardiotoxicity in Adult Rats.* Biol Trace Elem Res, 2016. **169**(2): p. 261-70.
174. Chaabane, M., et al., *Nitraria retusa fruit prevents penconazole-induced kidney injury in adult rats through modulation of oxidative stress and histopathological changes.* Pharm Biol, 2017. **55**(1): p. 1061-1073.
175. Qadir, S. and F. Iqbal, *Effect of subleathal concentrtion of imidacloprid on the histology of heart, liver and kidney in Labeo rohitr.* Pak J Pharm Sci, 2016. **29**(6): p. 2033-2038.
176. Ore, A. and E.T. Olayinka, *Fluazifop- p-butyl, an aryloxyphenoxypropionate herbicide, diminishes renal and hepatic functions and triggers testicular oxidative stress in orally exposed rats.* Toxicol Ind Health, 2017. **33**(5): p. 406-415.
177. Yan, S., et al., *Toxic effects of nitenpyram on antioxidant enzyme system and DNA in zebrafish (Danio rerio) livers.* Ecotoxicol Environ Saf, 2015. **122**: p. 54-60.
178. Hendawi, M.Y., R.T. Alam, and S.A. Abdellatief, *Ameliorative effect of flaxseed oil against thiacloprid-induced toxicity in rats: hematological, biochemical, and histopathological study.* Environ Sci Pollut Res Int, 2016. **23**(12): p. 11855-63.

179. George, J. and Y. Shukla, *Emptying of Intracellular Calcium Pool and Oxidative Stress Imbalance Are Associated with the Glyphosate-Induced Proliferation in Human Skin Keratinocytes HaCaT Cells.* ISRN dermatology, 2013. **2013**: p. 825180-825180.
180. Kim, Y.-h., et al., *Mixtures of glyphosate and surfactant TN20 accelerate cell death via mitochondrial damage-induced apoptosis and necrosis.* Toxicology in Vitro, 2013. **27**(1): p. 191-197.
181. Gao, S., et al., *Oral Exposure to Atrazine Induces Oxidative Stress and Calcium Homeostasis Disruption in Spleen of Mice.* Oxidative Medicine and Cellular Longevity, 2016. **2016**: p. 7978219.
182. Abass, M.A., et al., *Lycopene ameliorates atrazine-induced oxidative damage in adrenal cortex of male rats by activation of the Nrf2/HO-1 pathway.* Environ Sci Pollut Res Int, 2016. **23**(15): p. 15262-74.
183. Liu, W., et al., *Effects of atrazine on the oxidative damage of kidney in Wister rats.* International journal of clinical and experimental medicine, 2014. **7**(10): p. 3235-3243.
184. Ma, K., et al., *Neurotoxicity effects of atrazine-induced SH-SY5Y human dopaminergic neuroblastoma cells via microglial activation.* Molecular BioSystems, 2015. **11**(11): p. 2915-2924.
185. Song, X.-Y., et al., *Atrazine Causes Autophagy- and Apoptosis-Related Neurodegenerative Effects in Dopaminergic Neurons in the Rat Nigrostriatal Dopaminergic System.* International journal of molecular sciences, 2015. **16**(6): p. 13490-13506.
186. Sharma, S., et al., *Melatonin Reverses Fas, E2F-1 and Endoplasmic Reticulum Stress Mediated Apoptosis and Dysregulation of Autophagy Induced by the Herbicide Atrazine in Murine Splenocytes.* PLOS ONE, 2014. **9**(9): p. e108602.
187. Zhang, D., et al., *Exposure to 2,4-dichlorophenoxyacetic acid induces oxidative stress and apoptosis in mouse testis.* Pesticide Biochemistry and Physiology, 2017. **141**: p. 18-22.
188. Song, X., et al., *Study on systemic and reproductive toxicity of acetochlor in male mice.* Toxicology Research, 2018. **8**(1): p. 77-89.
189. Jiang, J., et al., *Effect of acetochlor on transcription of genes associated with oxidative stress, apoptosis, immunotoxicity and endocrine disruption in the early life stage of zebrafish.* Environmental Toxicology and Pharmacology, 2015. **40**(2): p. 516-523.
190. Goswami, D.G., et al., *Toxic consequences and oxidative protein carbonylation from chloropicrin exposure in human corneal epithelial cells.* Toxicology Letters, 2020. **322**: p. 1-11.
191. Pesonen, M., et al., *Chloropicrin-induced toxic responses in human lung epithelial cells.* Toxicology Letters, 2014. **226**(2): p. 236-244.
192. Pesonen, M., et al., *Chloropicrin induces endoplasmic reticulum stress in human retinal pigment epithelial cells.* Toxicology Letters, 2012. **211**(3): p. 239-245.
193. Calviello, G., et al., *DNA damage and apoptosis induction by the pesticide Mancozeb in rat cells: Involvement of the oxidative mechanism.* Toxicology and Applied Pharmacology, 2006. **211**(2): p. 87-96.
194. Kumar, K., A. Sabarwal, and R.P. Singh, *Mancozeb selectively induces mitochondrial-mediated apoptosis in human gastric carcinoma cells through ROS generation.* Mitochondrion, 2019. **48**: p. 1-10.

195. Mohammadi-Sardoo, M., et al., *Mancozeb induces testicular dysfunction through oxidative stress and apoptosis: Protective role of N-acetylcysteine antioxidant.* Toxicology and Industrial Health, 2018. **34**(11): p. 798-811.
196. Singh, N., et al., *Organophosphate pesticide chlorpyrifos impairs STAT1 signaling to induce dopaminergic neurotoxicity: Implications for mitochondria mediated oxidative stress signaling events.* Neurobiology of Disease, 2018. **117**: p. 82-113.
197. Lee, J.E., et al., *Rosiglitazone inhibits chlorpyrifos-induced apoptosis via modulation of the oxidative stress and inflammatory response in SH-SY5Y cells.* Toxicology and Applied Pharmacology, 2014. **278**(2): p. 159-171.
198. Lee, J.E., et al., *Reactive oxygen species regulated mitochondria-mediated apoptosis in PC12 cells exposed to chlorpyrifos.* Toxicology and Applied Pharmacology, 2012. **263**(2): p. 148-162.
199. Zhao, Y., et al., *Walnut Polyphenol Extract Protects against Malathion- and Chlorpyrifos-Induced Immunotoxicity by Modulating TLRx-NOX-ROS.* Nutrients, 2020. **12**(3): p. 616.
200. Rodriguez-Rocha, H., et al., *Compartmentalized oxidative stress in dopaminergic cell death induced by pesticides and complex I inhibitors: Distinct roles of superoxide anion and superoxide dismutases.* Free Radical Biology and Medicine, 2013. **61**: p. 370-383.
201. Chang, X., et al., *Paraquat inhibits cell viability via enhanced oxidative stress and apoptosis in human neural progenitor cells.* Chemico-Biological Interactions, 2013. **206**(2): p. 248-255.
202. Chinta, S.J., et al., *Coupling endoplasmic reticulum stress to the cell death program in dopaminergic cells: effect of paraquat.* Neuromolecular medicine, 2008. **10**(4): p. 333-342.
203. Yang, Z., et al., *Endoplasmic reticulum stress-related neuroinflammation and neural stem cells decrease in mice exposure to paraquat.* Scientific Reports, 2020. **10**(1): p. 17757.
204. Yang, W., et al., *Paraquat activates the IRE1/ASK1/JNK cascade associated with apoptosis in human neuroblastoma SH-SY5Y cells.* Toxicology Letters, 2009. **191**(2): p. 203-210.
205. Song, C.Q., et al., *Effect of endoplasmic reticulum calcium on paraquat-induced apoptosis of human lung type II alveolar epithelial A549 cells.* Mol Med Rep, 2019. **20**(3): p. 2419-2425.
206. Niso-Santano, M., et al., *ASK1 Overexpression Accelerates Paraquat-Induced Autophagy via Endoplasmic Reticulum Stress.* Toxicological Sciences, 2010. **119**(1): p. 156-168.
207. Meng, X., et al., *Endoplasmic reticulum stress promotes epithelial-mesenchymal transition via the PERK signaling pathway in paraquat-induced pulmonary fibrosis.* Mol Med Rep, 2021. **24**(1): p. 525.
208. Dou, T., et al., *Nrf2/ARE Pathway Involved in Oxidative Stress Induced by Paraquat in Human Neural Progenitor Cells.* Oxidative Medicine and Cellular Longevity, 2016. **2016**: p. 8923860.
209. Wills, J., et al., *Paraquat, but Not Maneb, Induces Synucleinopathy and Tauopathy in Striata of Mice through Inhibition of Proteasomal and Autophagic Pathways.* PLOS ONE, 2012. **7**(1): p. e30745.
210. Carloni, M., et al., *Early life permethrin exposure induces long-term brain changes in Nurr1, NF-kB and Nrf-2.* Brain Research, 2013. **1515**: p. 19-28.

211. Fedeli, D., et al., *Leukocyte Nurr1 as peripheral biomarker of early-life environmental exposure to permethrin insecticide.* Biomarkers, 2012. **17**(7): p. 604-9.
212. Dhivya Vadhana, M.S., et al., *Early life permethrin treatment leads to long-term cardiotoxicity.* Chemosphere, 2013. **93**(6): p. 1029-34.
213. Li, H.Y., et al., *The pesticide deltamethrin increases free radical production and promotes nuclear translocation of the stress response transcription factor Nrf2 in rat brain.* Toxicol Ind Health, 2011. **27**(7): p. 579-90.
214. Bouaziz, C., et al., *Influence of bifentrin, a pyrethriod pesticide, on human colorectal HCT-116 cells attributed to alterations in oxidative stress involving mitochondrial apoptotic processes.* Journal of Toxicology and Environmental Health, Part A, 2020. **83**(9): p. 331-340.
215. Park, S., et al., *Bifenthrin induces developmental immunotoxicity and vascular malformation during zebrafish embryogenesis.* Comp Biochem Physiol C Toxicol Pharmacol, 2020. **228**: p. 108671.
216. Kašuba, V., et al., *Effects of low doses of glyphosate on DNA damage, cell proliferation and oxidative stress in the HepG2 cell line.* Environmental Science and Pollution Research, 2017. **24**(23): p. 19267-19281.
217. El-Shenawy, N.S., *Oxidative stress responses of rats exposed to Roundup and its active ingredient glyphosate.* Environmental Toxicology and Pharmacology, 2009. **28**(3): p. 379-385.
218. Bailey, D.C., et al., *Chronic exposure to a glyphosate-containing pesticide leads to mitochondrial dysfunction and increased reactive oxygen species production in Caenorhabditis elegans.* Environmental Toxicology and Pharmacology, 2018. **57**: p. 46-52.
219. Romero, D.M., M.C. Ríos de Molina, and Á.B. Juárez, *Oxidative stress induced by a commercial glyphosate formulation in a tolerant strain of Chlorella kessleri.* Ecotoxicology and Environmental Safety, 2011. **74**(4): p. 741-747.
220. El-Shenawy, N., *Mitigating effect of ginger against oxidative stress induced by atrazine herbicides in mice liver and kidney.* Journal of Biofertilizers & Biopesticides. 2 (1): 107, 2011.
221. Singh, M., R. Sandhir, and R. Kiran, *Oxidative stress induced by atrazine in rat erythrocytes: Mitigating effect of vitamin E.* Toxicology Mechanisms and Methods, 2010. **20**(3): p. 119-126.
222. Abarikwu, S.O., et al., *Changes in Sperm Characteristics and Induction of Oxidative Stress in the Testis and Epididymis of Experimental Rats by a Herbicide, Atrazine.* Archives of Environmental Contamination and Toxicology, 2010. **58**(3): p. 874-882.
223. Adedosu, O., et al., *708 - Telfairia occidentalis seed extract protects against oxidative stress, inflammation and some haematological disorders associated with atrazine-induced prostate cancer in rats.* European Journal of Cancer, 2017. **72**: p. S94.
224. Keshk, W.A., et al., *Modulatory effects of curcumin on redox status, mitochondrial function, and caspace-3 expression during atrazin-induced toxicity.* J Biochem Mol Toxicol, 2014. **28**(8): p. 378-85.
225. Liu, H., et al., *Enantioselective oxidative stress and oxidative damage caused by Rac- and S-metolachlor to Scenedesmus obliquus.* Chemosphere, 2017. **173**: p. 22-30.

226. Špoljarić Maronić, D., et al., *S-metolachlor promotes oxidative stress in green microalga Parachlorella kessleri - A potential environmental and health risk for higher organisms.* Science of The Total Environment, 2018. **637-638**: p. 41-49.
227. Qu, Q., et al., *Enantioselective Oxidative Stress Induced by S- and Rac-metolachlor in Wheat (Triticum aestivum L.) Seedlings.* Bulletin of Environmental Contamination and Toxicology, 2019. **102**(3): p. 439-445.
228. Bernat, P., et al., *2,4-dichlorophenoxyacetic acid-induced oxidative stress: Metabolome and membrane modifications in Umbelopsis isabellina, a herbicide degrader.* PLOS ONE, 2018. **13**(6): p. e0199677.
229. ROMERO-PUERTAS, M.C., et al., *Reactive oxygen species-mediated enzymatic systems involved in the oxidative action of 2,4-dichlorophenoxyacetic acid*.* Plant, Cell & Environment, 2004. **27**(9): p. 1135-1148.
230. Peixoto, F.P., et al., *Comparative effects of the herbicides dicamba, 2,4-D and paraquat on non-green potato tuber calli.* Journal of Plant Physiology, 2008. **165**(11): p. 1125-1133.
231. Atamaniuk, T.M., et al., *Oxidative stress as a mechanism for toxicity of 2,4-dichlorophenoxyacetic acid (2,4-D): studies with goldfish gills.* Ecotoxicology, 2013. **22**(10): p. 1498-1508.
232. es Ruiz de Arcaute, C., et al., *Auxinic herbicides induce oxidative stress on Cnesterodon decemmaculatus (Pisces: Poeciliidae).* Environmental Science and Pollution Research, 2019. **26**(20): p. 20485-20498.
233. Liu, Z., et al., *Fluazifop-P-butyl induced ROS generation with IAA (indole-3-acetic acid) oxidation in Acanthospermum hispidum D.C.* Pesticide Biochemistry and Physiology, 2017. **143**: p. 312-318.
234. Bongiovanni, B., et al., *Melatonin decreases the oxidative stress produced by 2,4-dichlorophenoxyacetic acid in rat cerebellar granule cells.* Neurotoxicity Research, 2007. **11**(2): p. 93.
235. Troudi, A., et al., *Oxidative stress induced by 2,4-phenoxyacetic acid in liver of female rats and their progeny: biochemical and histopathological studies.* Environ Toxicol, 2012. **27**(3): p. 137-45.
236. Tichati, L., F. Trea, and K. Ouali, *Potential Role of Selenium Against Hepatotoxicity Induced by 2,4-Dichlorophenoxyacetic Acid in Albino Wistar Rats.* Biological Trace Element Research, 2020. **194**(1): p. 228-236.
237. Tayeb, W., et al., *Alteration of lipid status and lipid metabolism, induction of oxidative stress and lipid peroxidation by 2,4-dichlorophenoxyacetic herbicide in rat liver.* Toxicology Mechanisms and Methods, 2013. **23**(6): p. 449-458.
238. Tayeb, W., et al., *Biochemical and histological evaluation of kidney damage after sub-acute exposure to 2,4-dichlorophenoxyacetic herbicide in rats: involvement of oxidative stress.* Toxicology Mechanisms and Methods, 2012. **22**(9): p. 696-704.
239. Tayeb, W., et al., *Hepatotoxicity induced by sub-acute exposure of rats to 2,4-Dichlorophenoxyacetic acid based herbicide "Désormone lourd".* Journal of Hazardous Materials, 2010. **180**(1): p. 225-233.
240. Pruett, S.B., et al., *Oxidative stress and sodium methyldithiocarbamate-induced modulation of the macrophage response to lipopolysaccharide in vivo.* Toxicological sciences : an official journal of the Society of Toxicology, 2009. **109**(2): p. 237-246.

241. Martins, P.F., et al., *Effects of the herbicides acetochlor and metolachlor on antioxidant enzymes in soil bacteria.* Process Biochemistry, 2011. **46**(5): p. 1186-1195.
242. Liu, Y., et al., *The role of reactive oxygen species in the herbicide acetochlor-induced DNA damage on Bufo raddei tadpole liver.* Aquatic Toxicology, 2006. **78**(1): p. 21-26.
243. Zhang, Y., et al., *Acetochlor affects zebrafish ovarian development by producing estrogen effects and inducing oxidative stress.* Environmental Science and Pollution Research, 2020. **27**(22): p. 27688-27696.
244. Huang, T., et al., *Toxicity assessment of the herbicide acetochlor in the human liver carcinoma (HepG2) cell line.* Chemosphere, 2020. **243**: p. 125345.
245. Haque, M.N., et al., *Chlorothalonil induces oxidative stress and reduces enzymatic activities of Na+/K+-ATPase and acetylcholinesterase in gill tissues of marine bivalves.* PLOS ONE, 2019. **14**(4): p. e0214236.
246. da Silva Barreto, J., et al., *Induction of oxidative stress by chlorothalonil in the estuarine polychaete Laeonereis acuta.* Aquatic Toxicology, 2018. **196**: p. 1-8.
247. da Silva Barreto, J., F. de Melo Tarouco, and C.E. da Rosa, *Chlorothalonil causes redox state change leading to oxidative stress generation in Danio rerio.* Aquatic Toxicology, 2020. **225**: p. 105527.
248. Suzuki, T., et al., *Oxidative damages in isolated rat hepatocytes treated with the organochlorine fungicides captan, dichlofluanid and chlorothalonil.* Toxicology, 2004. **204**(2): p. 97-107.
249. Cima, F., M. Bragadin, and L. Ballarin, *Toxic effects of new antifouling compounds on tunicate haemocytes: I. Sea-Nine 211™ and chlorothalonil.* Aquatic Toxicology, 2008. **86**(2): p. 299-312.
250. Lodovici, M., et al., *Oxidative liver DNA damage in rats treated with pesticide mixtures.* Toxicology, 1997. **117**(1): p. 55-60.
251. Ahmad, I. and M. Ahmad, *Fresh water fish, Channa punctatus, as a model for pendimethalin genotoxicity testing: A new approach toward aquatic environmental contaminants.* Environmental Toxicology, 2016. **31**(11): p. 1520-1529.
252. Ansari, S.M., et al., *Pendimethalin induces oxidative stress, DNA damage, and mitochondrial dysfunction to trigger apoptosis in human lymphocytes and rat bone-marrow cells.* Histochemistry and Cell Biology, 2018. **149**(2): p. 127-141.
253. Gupta, P. and S.K. Verma, *Evaluation of genotoxicity induced by herbicide pendimethalin in fresh water fish Clarias batrachus (linn.) and possible role of oxidative stress in induced DNA damage.* Drug and Chemical Toxicology, 2020: p. 1-10.
254. Tabassum, H., et al., *Neurotoxicological assessment of pendimethalin in freshwater fish Channa punctata Bloch.* Ecological Indicators, 2015. **58**: p. 411-417.
255. Tabassum, H., et al., *Short term exposure of pendimethalin induces biochemical and histological perturbations in liver, kidney and gill of freshwater fish.* Ecological Indicators, 2016. **63**: p. 29-36.
256. Sarıgöl Kılıç, Z., et al., *In vitro genotoxicity assessment of dinitroaniline herbicides pendimethalin and trifluralin.* Food and Chemical Toxicology, 2018. **113**: p. 90-98.
257. Abouzeid, S., et al., *Ameliorating Effects of Green Tea on Ethephon-Induced Immunotoxicity and Oxidative Stress In Mice.* International Journal of Pharma Sciences and Scientific Research, 2018. **4**.
258. Hodjat, M., et al., *On the mechanism of genotoxicity of ethephon on embryonic fibroblast cells.* Toxicology Mechanisms and Methods, 2017. **27**(3): p. 173-180.

259. Öztürk, L. and Y. Demir, *Effects of putrescine and ethephon on some oxidative stress enzyme activities and proline content in salt stressed spinach leaves.* Plant Growth Regulation, 2003. **40**(1): p. 89-95.
260. Sun, Z.-Y., et al., *A novel role of ethephon in controlling the noxious weed Ipomoea cairica (Linn.) Sweet.* Scientific Reports, 2015. **5**(1): p. 11372.
261. Xiang-Cai, M., et al., *Effects of H₂O₂, paraquat, and ethephon on herbal drug quality of <i>Schiandra chinensis</i> based on reactive oxygen species system.* Pharmacognosy Magazine, 2012. **8**(29): p. 54-59.
262. Kubrak, O.I., et al., *Oxidative stress responses in blood and gills of Carassius auratus exposed to the mancozeb-containing carbamate fungicide Tattoo.* Ecotoxicology and Environmental Safety, 2012. **85**: p. 37-43.
263. Atamaniuk, T.M., et al., *The mancozeb-containing carbamate fungicide tattoo induces mild oxidative stress in goldfish brain, liver, and kidney.* Environmental Toxicology, 2014. **29**(11): p. 1227-1235.
264. Majid, U., et al., *Antioxidant response of Cassia angustifolia Vahl. to oxidative stress caused by Mancozeb, a pyrethroid fungicide.* Acta Physiologiae Plantarum, 2014. **36**(2): p. 307-314.
265. Bailey, D.C., et al., *Caenorhabditis elegans chronically exposed to a Mn/Zn ethylene-bis-dithiocarbamate fungicide show mitochondrial Complex I inhibition and increased reactive oxygen species.* NeuroToxicology, 2016. **56**: p. 170-179.
266. Todt, C.E., et al., *Acute exposure to a Mn/Zn ethylene-bis-dithiocarbamate fungicide leads to mitochondrial dysfunction and increased reactive oxygen species production in Caenorhabditis elegans.* NeuroToxicology, 2016. **57**: p. 112-120.
267. Saraiva, M.A., et al., *Exposure of <i>Drosophila melanogaster</i> to Mancozeb Induces Oxidative Damage and Modulates Nrf2 and HSP70/83.* Oxidative Medicine and Cellular Longevity, 2018. **2018**: p. 5456928.
268. Pavlovic, V., et al., *The Effect of Ascorbic Acid on Mancozeb-Induced Toxicity in Rat Thymocytes.* Folia Biol (Praha), 2015. **61**(3): p. 116-23.
269. Mansour, S.A. and A.-T.H. Mossa, *Lipid peroxidation and oxidative stress in rat erythrocytes induced by chlorpyrifos and the protective effect of zinc.* Pesticide Biochemistry and Physiology, 2009. **93**(1): p. 34-39.
270. Uchendu, C., et al., *Chronic co-exposure to chlorpyrifos and deltamethrin pesticides induces alterations in serum lipids and oxidative stress in Wistar rats: mitigating role of alpha-lipoic acid.* Environ Sci Pollut Res Int, 2018. **25**(20): p. 19605-19611.
271. Alvarez, A.A., E. Ramírez-San Juan, and A. Canizales-Román, *Chlorpyrifos induces oxidative stress in rats.* Toxicological & Environmental Chemistry, 2008. **90**(5): p. 1019-1025.
272. Mandal, T.K. and N.S. Das, *Correlation of testicular toxicity and oxidative stress induced by chlorpyrifos in rats.* Hum Exp Toxicol, 2011. **30**(10): p. 1529-39.
273. Imam, A., et al., *Chlorpyrifos- and Dichlorvos-Induced Oxidative and Neurogenic Damage Elicits Neuro-Cognitive Deficits and Increases Anxiety-Like Behavior in Wild-Type Rats.* Toxics, 2018. **6**(4): p. 71.
274. BaŞ, H. and Y. Kalender, *Chlorpyrifos induced cardiotoxicity in rats and the protective role of quercetin and catechin.* Gazi University Journal of Science, 2011. **24**: p. 385-395.
275. Štajner, D., M. Popović, and M. Štajner, *Herbicide Induced Oxidative Stress in Lettuce, Beans, Pea Seeds and Leaves.* Biologia plantarum, 2003. **46**(4): p. 575-579.

276. Otuechere, C., et al., *Oral Supplementation of Coconut Oil Attenuates Propanil-induced Oxidative Stress in the Testes of Rats.* Asian Journal of Biological Sciences, 2020. **13**.
277. Otuechere, C., et al., *Protective effects of vitamin C against propanil-induced hepatotoxicity in wistar rats.* Asian Pacific Journal of Tropical Disease, 2012. **2**: p. S212–S217.
278. Rehman, M., et al., *Propanil-induced neurotoxicity in freshwater common carp (Cyprinus carpio).* Applied Biological Research, 2016. **18**: p. 312.
279. Peixoto, F., J.A. Vicente, and V.M. Madeira, *The herbicide dicamba (2-methoxy-3,6-dichlorobenzoic acid) interacts with mitochondrial bioenergetic functions.* Arch Toxicol, 2003. **77**(7): p. 403-9.
280. Gleason, C., R.C. Foley, and K.B. Singh, *Mutant Analysis in Arabidopsis Provides Insight into the Molecular Mode of Action of the Auxinic Herbicide Dicamba.* PLOS ONE, 2011. **6**(3): p. e17245.
281. Kumar, M.S., et al., *Insecticides induced biochemical changes in freshwater microalga Chlamydomonas mexicana.* Environmental science and pollution research international, 2016. **23**(2): p. 1091-1099.
282. Rajak, P., et al., *Chronic exposure to acephate triggers ROS-mediated injuries at organismal and sub-organismal levels of Drosophila melanogaster.* Toxicol Res (Camb), 2018. **7**(5): p. 874-887.
283. Datta, S., et al., *Influence of polyphenolic extracts from Enydra fluctuans on oxidative stress induced by acephate in rats.* Food and Chemical Toxicology, 2010. **48**(10): p. 2766-2771.
284. Thapar, A., R. Sandhir, and R. Kiran, *Acephate induced oxidative damage in erythrocytes.* Indian J Exp Biol, 2002. **40**(8): p. 963-6.
285. Sharma, P., et al., *Organophosphorous compounds and oxidative stress: a review.* Toxicological & Environmental Chemistry, 2014. **96**(5): p. 681-698.
286. Dhanushka, M.A.T. and L.D.C. Peiris, *Cytotoxic and Genotoxic Effects of Acephate on Human Sperm.* Journal of Toxicology, 2017. **2017**: p. 3874817.
287. Al-Sarar, A.S., et al., *Cytotoxic effects of acephate, ethoprophos, and monocrotophos in CHO-K1 cells.* CyTA - Journal of Food, 2015. **13**(3): p. 427-433.
288. Cochemé, H.M. and M.P. Murphy, *Complex I is the major site of mitochondrial superoxide production by paraquat.* J Biol Chem, 2008. **283**(4): p. 1786-98.
289. Shimizu, K., et al., *Paraquat induces long-lasting dopamine overflow through the excitotoxic pathway in the striatum of freely moving rats.* Brain Research, 2003. **976**(2): p. 243-252.
290. Wu, D.-C., et al., *NADPH oxidase mediates oxidative stress in the 1-methyl-4-phenyl-1,2,3,6-tetrahydropyridine model of Parkinson's disease.* Proceedings of the National Academy of Sciences of the United States of America, 2003. **100**(10): p. 6145-6150.
291. Castello, P.R., D.A. Drechsel, and M. Patel, *Mitochondria are a major source of paraquat-induced reactive oxygen species production in the brain.* J Biol Chem, 2007. **282**(19): p. 14186-93.
292. Ranjbar, A., et al., *Induction of oxidative stress in paraquat formulating workers.* Toxicology Letters, 2002. **131**(3): p. 191-194.
293. Castello, P.R., D.A. Drechsel, and M. Patel, *Mitochondria are a major source of paraquat-induced reactive oxygen species production in the brain.* The Journal of biological chemistry, 2007. **282**(19): p. 14186-14193.

294. Takano, H.K., et al., *A novel insight into the mode of action of glufosinate: how reactive oxygen species are formed.* Photosynth Res, 2020. **144**(3): p. 361-372.
295. Takano, H.K., et al., *Reactive oxygen species trigger the fast action of glufosinate.* Planta, 2019. **249**(6): p. 1837-1849.
296. Qian, H., et al., *Effects of glufosinate on antioxidant enzymes, subcellular structure, and gene expression in the unicellular green alga Chlorella vulgaris.* Aquatic Toxicology, 2008. **88**(4): p. 301-307.
297. Saquib, Q., et al., *Phorate-induced oxidative stress, DNA damage and transcriptional activation of p53 and caspase genes in male Wistar rats.* Toxicology and Applied Pharmacology, 2012. **259**(1): p. 54-65.
298. Saquib, Q., et al., *Preferential binding of insecticide phorate with sub-domain IIA of human serum albumin induces protein damage and its toxicological significance.* Food and Chemical Toxicology, 2011. **49**(8): p. 1787-1795.
299. Saquib, Q., et al., *Phorate triggers oxidative stress and mitochondrial dysfunction to enhance micronuclei generation and DNA damage in human lymphocytes.* Saudi Journal of Biological Sciences, 2019. **26**(7): p. 1411-1417.
300. Poovala, V.S., H. Huang, and A.K. Salahudeen, *Role of reactive oxygen metabolites in organophosphate-bidrin-induced renal tubular cytotoxicity.* J Am Soc Nephrol, 1999. **10**(8): p. 1746-52.
301. Wu, J.-C., et al., *Genotoxicity of dicrotophos, an organophosphorous pesticide, assessed with different assays in vitro.* Environmental Toxicology, 2012. **27**(5): p. 307-315.
302. Hseu, Y.-C., et al., *Molecular mechanisms of discrotophos-induced toxicity in HepG2 cells: The role of CSA in oxidative stress.* Food and Chemical Toxicology, 2017. **103**: p. 253-260.
303. Liu, X., et al., *Photocatalytic degradation of dimethoate in Bok choy using cerium-doped nano titanium dioxide.* PLOS ONE, 2018. **13**(5): p. e0197560.
304. Nazam, N., et al., *Combined in silico and in vivo studies shed insights into the acute acetylcholinesterase response in rat and human brain.* Biotechnology and Applied Biochemistry, 2015. **62**(3): p. 407-415.
305. Nazam, N., et al., *Dimethoate Induces DNA Damage and Mitochondrial Dysfunction Triggering Apoptosis in Rat Bone-Marrow and Peripheral Blood Cells.* Toxics, 2020. **8**(4).
306. Gargouri, B., et al., *Protective effect of quercetin against oxidative stress caused by dimethoate in human peripheral blood lymphocytes.* Lipids Health Dis, 2011. **10**: p. 149.
307. Serdar, O., *The effect of dimethoate pesticide on some biochemical biomarkers in Gammarus pulex.* Environ Sci Pollut Res Int, 2019. **26**(21): p. 21905-21914.
308. Ben Amara, I., et al., *Antioxidant effect of vitamin E and selenium on hepatotoxicity induced by dimethoate in female adult rats.* Ecotoxicology and Environmental Safety, 2011. **74**(4): p. 811-819.
309. Amara, I.B., et al., *Dimethoate-induced oxidative damage in erythrocytes of female adult rats:possible protective effect of vitamin E and selenium supplemented to diet.* Toxicology and Industrial Health, 2012. **28**(3): p. 222-237.
310. John, S., et al., *Protective effect of vitamin E in dimethoate and malathion induced oxidative stress in rat erythrocytes.* The Journal of Nutritional Biochemistry, 2001. **12**(9): p. 500-504.

311. Ali, D., et al., *Evaluation of genotoxic and oxidative stress response to dimethoate in freshwater fish Channa punctatus (Bloch).* Chemical Speciation & Bioavailability, 2014. **26**(2): p. 111-118.
312. Dogan, D., et al., *Dimethoate-induced oxidative stress and DNA damage in Oncorhynchus mykiss.* Chemosphere, 2011. **84**(1): p. 39-46.
313. Shadegan, M.R. and M. Banaee, *Effects of dimethoate alone and in combination with Bacilar fertilizer on oxidative stress in common carp, Cyprinus carpio.* Chemosphere, 2018. **208**: p. 101-107.
314. Hung, J.-H., et al., *Reactive oxygen species mediate Terbufos-induced apoptosis in mouse testicular cell lines via the modulation of cell cycle and pro-apoptotic proteins.* Environmental Toxicology, 2016. **31**(12): p. 1888-1898.
315. Olgun, S. and H.P. Misra, *Pesticides induced oxidative stress in thymocytes.* Molecular and Cellular Biochemistry, 2006. **290**(1): p. 137.
316. Jin, X., et al., *Protective Efficacy of Vitamins C and E on p,p'-DDT-Induced Cytotoxicity via the ROS-Mediated Mitochondrial Pathway and NF-κB/FasL Pathway.* PLOS ONE, 2014. **9**(12): p. e113257.
317. Barlow, B.K., et al., *Modulation of Antioxidant Defense Systems by the Environmental Pesticide Maneb in Dopaminergic Cells.* NeuroToxicology, 2005. **26**(1): p. 63-75.
318. Domico, L.M., et al., *Reactive oxygen species generation by the ethylene-bis-dithiocarbamate (EBDC) fungicide mancozeb and its contribution to neuronal toxicity in mesencephalic cells.* NeuroToxicology, 2007. **28**(6): p. 1079-1091.
319. Kitazawa, M., V. Anantharam, and A.G. Kanthasamy, *Dieldrin-induced oxidative stress and neurochemical changes contribute to apoptopic cell death in dopaminergic cells.* Free Radical Biology and Medicine, 2001. **31**(11): p. 1473-1485.
320. Stedeford, T., et al., *Comparison of base-excision repair capacity in proliferating and differentiated PC 12 cells following acute challenge with dieldrin.* Free Radical Biology and Medicine, 2001. **31**(10): p. 1272-1278.
321. Kitazawa, M., V. Anantharam, and A.G. Kanthasamy, *Dieldrin induces apoptosis by promoting caspase-3-dependent proteolytic cleavage of protein kinase Cδ in dopaminergic cells: relevance to oxidative stress and dopaminergic degeneration.* Neuroscience, 2003. **119**(4): p. 945-964.
322. . Chun, H.S., et al., *Dopaminergic cell death induced by MPP+, oxidant and specific neurotoxicants shares the common molecular mechanism.* Journal of Neurochemistry, 2001. **76**(4): p. 1010-1021.
323. KANTHASAMY, A.G., et al., *Proteolytic Activation of Proapoptotic Kinase PKCδ Is Regulated by Overexpression of Bcl-2.* Annals of the New York Academy of Sciences, 2003. **1010**(1): p. 683-686.
324. Sun, F., et al., *Dieldrin Induces Ubiquitin-Proteasome Dysfunction in α-Synuclein Overexpressing Dopaminergic Neuronal Cells and Enhances Susceptibility to Apoptotic Cell Death.* Journal of Pharmacology and Experimental Therapeutics, 2005. **315**(1): p. 69-79.
325. Mao, H., et al., *Induction of microglial reactive oxygen species production by the organochlorinated pesticide dieldrin.* Brain Research, 2007. **1186**: p. 267-274.
326. Mangum, L.C., et al., *Organochlorine insecticides induce NADPH oxidase-dependent reactive oxygen species in human monocytic cells via phospholipase A2/arachidonic acid.* Chemical research in toxicology, 2015. **28**(4): p. 570-584.

327. Sava, V., et al., *Dieldrin elicits a widespread DNA repair and antioxidative response in mouse brain.* Journal of Biochemical and Molecular Toxicology, 2007. **21**(3): p. 125-135.
328. Hatcher, J.M., et al., *Dieldrin exposure induces oxidative damage in the mouse nigrostriatal dopamine system.* Experimental Neurology, 2007. **204**(2): p. 619-630.
329. Bal, R., et al., *Effects of the neonicotinoid insecticide, clothianidin, on the reproductive organ system in adult male rats.* Drug Chem Toxicol, 2013. **36**(4): p. 421-9.
330. Bal, R., et al., *Effects of clothianidin exposure on sperm quality, testicular apoptosis and fatty acid composition in developing male rats.* Cell Biol Toxicol, 2012. **28**(3): p. 187-200.
331. Chen, H.-M., et al., *The Pentachlorophenol Metabolite Tetrachlorohydroquinone Induces Massive ROS and Prolonged p-ERK Expression in Splenocytes, Leading to Inhibition of Apoptosis and Necrotic Cell Death.* PLOS ONE, 2014. **9**(2): p. e89483.
332. Umemura, T., et al., *Oxidative DNA Damage and Cell Proliferation in the Livers of B6C3F1 Mice Exposed to Pentachlorophenol in Their Diet.* Fundamental and Applied Toxicology, 1996. **30**(2): p. 285-289.
333. Téllez-Bañuelos, M.C., et al., *Low-dose endosulfan inhibits proliferation and induces senescence and pro-inflammatory cytokine production in human lymphocytes, preferentially impacting cytotoxic cells.* Journal of Immunotoxicology, 2019. **16**(1): p. 173-181.
334. Jia, Z. and H.P. Misra, *Exposure to mixtures of endosulfan and zineb induces apoptotic and necrotic cell death in SH-SY5Y neuroblastoma cells, in vitro.* Journal of Applied Toxicology, 2007. **27**(5): p. 434-446.
335. Zawada, W.M., et al., *Generation of reactive oxygen species in 1-methyl-4-phenylpyridinium (MPP+) treated dopaminergic neurons occurs as an NADPH oxidase-dependent two-wave cascade.* J Neuroinflammation, 2011. **8**: p. 129.
336. Gao, H.M., et al., *Distinct role for microglia in rotenone-induced degeneration of dopaminergic neurons.* J Neurosci, 2002. **22**(3): p. 782-90.
337. Li, Q., M. Kobayashi, and T. Kawada, *Mechanism of ziram-induced apoptosis in human natural killer cells.* Int J Immunopathol Pharmacol, 2012. **25**(4): p. 883-91.
338. Xie, L., et al., *Delayed Puberty by Ziram Is Associated with Down Regulation of Testicular Phosphorylated AKT1 and SIRT1/PGC-1α Signaling.* Chem Res Toxicol, 2018. **31**(12): p. 1315-1322.
339. Ramirez-Vargas, M.A., et al., *Methamidophos induces cytotoxicity and oxidative stress in human peripheral blood mononuclear cells.* Environ Toxicol, 2017. **32**(1): p. 147-155.
340. Imam, A., et al., *Chlorpyrifos- and Dichlorvos-Induced Oxidative and Neurogenic Damage Elicits Neuro-Cognitive Deficits and Increases Anxiety-Like Behavior in Wild-Type Rats.* Toxics, 2018. **6**(4).
341. Eroğlu, S., et al., *Protective role of vitamins C and E in dichlorvos-induced oxidative stress in human erythrocytes in vitro.* Biol Res, 2013. **46**(1): p. 33-8.
342. Ben Salem, I., et al., *Dichlorvos-induced toxicity in HCT116 cells: Involvement of oxidative stress and apoptosis.* Pesticide Biochemistry and Physiology, 2015. **119**: p. 62-66.
343. Ben Salem, I., et al., *Quercetin protects HCT116 cells from Dichlorvos-induced oxidative stress and apoptosis.* Cell Stress Chaperones, 2016. **21**(1): p. 179-186.
344. Cankayali, I., et al., *The effects of N-acetylcysteine on oxidative stress in organophosphate poisoning model.* Adv Ther, 2005. **22**(2): p. 107-16.

345. Wani, W.Y., et al., *Protective efficacy of mitochondrial targeted antioxidant MitoQ against dichlorvos induced oxidative stress and cell death in rat brain.* Neuropharmacology, 2011. **61**(8): p. 1193-201.
346. Ben Othmène, Y., et al., *Tebuconazole induces ROS-dependent cardiac cell toxicity by activating DNA damage and mitochondrial apoptotic pathway.* Ecotoxicol Environ Saf, 2020. **204**: p. 111040.
347. Timoumi, R., et al., *Triflumuron induces genotoxicity in both mice bone marrow cells and human Colon cancer cell line.* Toxicol Mech Methods, 2020. **30**(6): p. 438-449.
348. Timoumi, R., et al., *Protective effects of fennel essential oil against oxidative stress and genotoxicity induced by the insecticide triflumuron in human colon carcinoma cells.* Environ Sci Pollut Res Int, 2020. **27**(8): p. 7957-7966.
349. Timoumi, R., et al., *Triflumuron induces cytotoxic effects on hepatic and renal human cell lines.* J Biochem Mol Toxicol, 2020. **34**(8): p. e22504.
350. Annabi, E., I. Ben Salem, and S. Abid-Essefi, *Acetamiprid, a neonicotinoid insecticide, induced cytotoxicity and genotoxicity in PC12 cells.* Toxicol Mech Methods, 2019. **29**(8): p. 580-586.
351. Kara, M., E. ÖztaŞ, and G. Özhan, *Acetamiprid-induced Cyto- and Genotoxicity in the AR42J Pancreatic Cell Line.* Turk J Pharm Sci, 2020. **17**(5): p. 474-479.
352. Devan, R.K., et al., *Sub-chronic oral toxicity of acetamiprid in Wistar rats.* Toxicological & Environmental Chemistry, 2015. **97**: p. 1236-1252.
353. Zhang, J.-j., et al., *Oxidative Stress: Role in Acetamiprid-Induced Impairment of the Male Mice Reproductive System.* Agricultural Sciences in China, 2011. **10**(5): p. 786-796.
354. Yang, Y., et al., *Biological response of zebrafish embryos after short-term exposure to thifluzamide.* Sci Rep, 2016. **6**: p. 38485.
355. Maran, E., et al., *Effects of aldicarb and propoxur on cytotoxicity and lipid peroxidation in CHO-K1 cells.* Food Chem Toxicol, 2010. **48**(6): p. 1592-6.
356. Yavuz, T., et al., *Vascular wall damage in rats induced by methidathion and ameliorating effect of vitamins E and C.* Arch Toxicol, 2004. **78**(11): p. 655-9.
357. Abdou, H.M. and R.H. ElMazoudy, *Oxidative damage, hyperlipidemia and histological alterations of cardiac and skeletal muscles induced by different doses of diazinon in female rats.* J Hazard Mater, 2010. **182**(1-3): p. 273-8.
358. Abdel-Daim, M.M., et al., *Synergistic ameliorative effects of sesame oil and alpha-lipoic acid against subacute diazinon toxicity in rats: hematological, biochemical, and antioxidant studies.* Can J Physiol Pharmacol, 2016. **94**(1): p. 81-8.
359. El-Shenawy, N.S., et al., *Amelioratory effect of vitamin E on organophosphorus insecticide diazinon-induced oxidative stress in mice liver.* Pesticide Biochemistry and Physiology, 2010. **96**(2): p. 101-107.
360. Hariri, A.T., et al., *Sub-acute effects of diazinon on biochemical indices and specific biomarkers in rats: protective effects of crocin and safranal.* Food Chem Toxicol, 2010. **48**(10): p. 2803-8.
361. Gokalp, O., et al., *The effects of diazinon on pancreatic damage and ameliorating role of vitamin E and vitamin C.* Pesticide Biochemistry and Physiology, 2005. **81**(2): p. 123-128.
362. Yaduvanshi, S.K., et al., *Monocrotophos induced lipid peroxidation and oxidative DNA damage in rat tissues.* Pesticide Biochemistry and Physiology, 2010. **97**(3): p. 214-222.

363. Baghaei, A., et al., *Molecular and biochemical evidence on the protection of cardiomyocytes from phosphine-induced oxidative stress, mitochondrial dysfunction and apoptosis by acetyl-L-carnitine.* Environ Toxicol Pharmacol, 2016. **42**: p. 30-7.
364. Solgi, R., et al., *Electrophysiological and molecular mechanisms of protection by iron sucrose against phosphine-induced cardiotoxicity: a time course study.* Toxicol Mech Methods, 2015. **25**(4): p. 249-57.
365. Al-Sarar, A.S., et al., *Cytotoxic and genotoxic effects of abamectin, chlorfenapyr, and imidacloprid on CHOK1 cells.* Environ Sci Pollut Res Int, 2015. **22**(21): p. 17041-52.
366. Annabi, A., et al., *Recovery by N-acetylcysteine from subchronic exposure to Imidacloprid-induced hypothalamic-pituitary-adrenal (HPA) axis tissues injury in male rats.* Toxicol Mech Methods, 2015. **25**(7): p. 524-31.
367. Lonare, M., et al., *Evaluation of ameliorative effect of curcumin on imidacloprid-induced male reproductive toxicity in wistar rats.* Environ Toxicol, 2016. **31**(10): p. 1250-63.
368. Lonare, M., et al., *Evaluation of imidacloprid-induced neurotoxicity in male rats: a protective effect of curcumin.* Neurochem Int, 2014. **78**: p. 122-9.
369. Bal, R., et al., *Insecticide imidacloprid induces morphological and DNA damage through oxidative toxicity on the reproductive organs of developing male rats.* Cell Biochem Funct, 2012. **30**(6): p. 492-9.
370. Duzguner, V. and S. Erdogan, *Chronic exposure to imidacloprid induces inflammation and oxidative stress in the liver & central nervous system of rats.* Pesticide Biochemistry and Physiology, 2012. **104**(1): p. 58-64.
371. Kapoor, U., M.K. Srivastava, and L.P. Srivastava, *Toxicological impact of technical imidacloprid on ovarian morphology, hormones and antioxidant enzymes in female rats.* Food Chem Toxicol, 2011. **49**(12): p. 3086-9.
372. Duzguner, V. and S. Erdogan, *Acute oxidant and inflammatory effects of imidacloprid on the mammalian central nervous system and liver in rats.* Pesticide Biochemistry and Physiology, 2010. **97**(1): p. 13-18.
373. Kapoor, U., et al., *Effect of imidacloprid on antioxidant enzymes and lipid peroxidation in female rats to derive its No Observed Effect Level (NOEL).* J Toxicol Sci, 2010. **35**(4): p. 577-81.
374. El-Gendy, K.S., et al., *The role of vitamin C as antioxidant in protection of oxidative stress induced by imidacloprid.* Food Chem Toxicol, 2010. **48**(1): p. 215-21.
375. Zhang, Q., B. Zhang, and C. Wang, *Ecotoxicological effects on the earthworm Eisenia fetida following exposure to soil contaminated with imidacloprid.* Environ Sci Pollut Res Int, 2014. **21**(21): p. 12345-53.
376. Balieira, K.V.B., et al., *Imidacloprid-induced oxidative stress in honey bees and the antioxidant action of caffeine.* Apidologie, 2018. **49**(5): p. 562-572.
377. Liu, Z., et al., *Fluazifop-P-butyl induced ROS generation with IAA (indole-3-acetic acid) oxidation in Acanthospermum hispidum D.C.* Pestic Biochem Physiol, 2017. **143**: p. 312-318.
378. Luo, X., et al., *Involvement of H(2)O(2) in fluazifop-P-butyl-induced cell death in bristly starbur seedlings.* Pestic Biochem Physiol, 2017. **143**: p. 258-264.
379. Luo, X.-Y., Y. Sunohara, and H. Matsumoto, *Fluazifop-butyl causes membrane peroxidation in the herbicide-susceptible broad leaf weed bristly starbur (Acanthospermum hispidum).* Pesticide Biochemistry and Physiology, 2004. **78**(2): p. 93-102.

380. Aydin, B., *Effects of thiacloprid, deltamethrin and their combination on oxidative stress in lymphoid organs, polymorphonuclear leukocytes and plasma of rats.* Pesticide Biochemistry and Physiology, 2011. **100**(2): p. 165-171.
381. Feng, L., et al., *Inhibition and recovery of biomarkers of earthworm Eisenia fetida after exposure to thiacloprid.* Environ Sci Pollut Res Int, 2015. **22**(12): p. 9475-82.
382. Peixoto, F., *Comparative effects of the Roundup and glyphosate on mitochondrial oxidative phosphorylation.* Chemosphere, 2005. **61**(8): p. 1115-1122.
383. Jin, Y., et al., *cis-Bifenthrin enantioselectively induces hepatic oxidative stress in mice.* Pesticide Biochemistry and Physiology, 2013. **107**(1): p. 61-67.
384. Moore, P.D., C.G. Yedjou, and P.B. Tchounwou, *Malathion-induced oxidative stress, cytotoxicity, and genotoxicity in human liver carcinoma (HepG2) cells.* Environmental Toxicology, 2010. **25**(3): p. 221-226.
